# Quinone reductase 2 *reads* H3 serotonylation to support neuronal maturation

**DOI:** 10.64898/2026.03.17.712426

**Published:** 2026-03-19

**Authors:** Min Chen, Celi Yang, Xin Li, Lingchun Kong, Benjamin H. Weekley, Xiaoran Wei, Jennifer C O’Chan, David A. Vinson, Bulent Cetin, Aarthi Ramakrishnan, Li Shen, Rongsheng Zeng, Zheng Liu, Juner Zhang, Kaylee M. Cappuccio, Joshua R. Sokol, Erdene Baljinnyam, Ruiqi Hu, Kobi Rosenblum, Henrik Molina, Qingfei Zheng, Yael David, Samuele G. Marro, Tom W. Muir, Xiang David Li, Haitao Li, Ian Maze

**Affiliations:** 1 Nash Family Department of Neuroscience, Friedman Brain Institute, Icahn School of Medicine at Mount Sinai, New York, New York 10029, USA; 2 Beijing Advanced Innovation Center for Structural Biology, MOE Key Laboratory of Protein Sciences, Department of Basic Medical Sciences, School of Medicine, Tsinghua University, Beijing 100084, China; 3 Institute of Chemical Biology, Shenzhen Bay Laboratory, Shenzhen 518132, China; 4 Department of Chemistry, University of Hong Kong, Hong Kong SAR, China; 5 Department of Chemistry, Princeton, New Jersey 08544, USA; 6 Black Family Stem Cell Institute, Icahn School of Medicine at Mount Sinai, New York, New York 10029, USA; 7 Department of Stem Cell Biology and Regenerative Medicine, Icahn School of Medicine at Mount Sinai, New York, New York 10029, USA; 8 Sagol Department of Neurobiology, Natural Science Faculty, Center for Gene Manipulation in the Brain, University of Haifa, 3498838, Haifa, Israel; 9 The Rockefeller University Proteomics Resource Center, The Rockefeller University, New York, NY 10065, USA; 10 Department of Medicinal Chemistry and Molecular Pharmacology, College of Pharmacy, Purdue University, West Lafayette, Indiana 47907, USA; 11 Institute for Cancer Research, Purdue University, West Lafayette, Indiana 47907, USA; 12 Chemical Biology Program, Memorial Sloan Kettering Cancer Center, New York, New York 10065, USA; 13 Tri-Institutional PhD Program in Chemical Biology, New York, New York 10065, USA; 14 Department of Physiology, Biophysics and Systems Biology, Weill Cornell Medicine, New York, New York 10065, USA; 15 Department of Pharmacology, Weill Cornell Medicine, New York, New York 10065, USA; 16 State Key Laboratory of Synthetic Chemistry, University of Hong Kong, Hong Kong SAR, China; 17 Department of Pharmacological Sciences, Icahn School of Medicine at Mount Sinai, New York, New York 10029, USA; 18 Howard Hughes Medical Institute, Icahn School of Medicine at Mount Sinai, New York, New York 10029, USA

## Abstract

Histone H3 Gln5 serotonylation (H3Q5ser) is a recently described posttranslational modification^1^ that plays important roles in guiding transcriptional permissiveness in brain and peripheral systems^2–5^. H3Q5ser has been implicated in diverse physiological and pathological processes ranging from neural differentiation^1^ to sensory processing^6^, circadian rhythmicity^7^, stress responsivity^8^, placental gene regulation^9^, and tumorigenesis^10–19^. Since H3Q5ser can occur in combination with H3 Lys4 trimethylation (H3K4me3), most mechanistic studies to date have focused on H3Q5ser’s roles in modulating H3K4me3 *reader* interactions, where it has been shown to potentiate TAF3/TFIID binding to H3K4me3^1,20,21^ and inhibit the recruitment of K4me3 demethylases^21^; however, whether H3 serotonylation functions as an autonomous chromatin signaling mark through dedicated *reader* proteins has remained unknown. Here, using a combination of proteomic-, structural-, molecular-, epigenomic-, and cellular-based approaches, we demonstrate that the Quinone reductase 2 (QR2) enzyme *reads* H3Q5ser independently of H3K4me3. CRISPR-Cas9-mediated disruption of H3 serotonylation or QR2’s binding to the mark in human induced pluripotent stem cell-derived neurons impairs the establishment of neuronal transcriptional programs, alters synaptic connectivity, and disrupts electrophysiological maturation. These findings thus uncover an H3 serotonylation-dependent chromatin signaling axis that is essential for human neurodevelopment.

## INTRODUCTION

Biogenic amines, such as serotonin (5-hydroxytryptamine or 5-HT), are a large class of evolutionarily ancient signaling molecules that contain one or more amino groups. These metabolites are found in diverse tissues in animals and can elicit biological effects locally at the site of production or remotely following transmission through the circulating blood system^2,22^. 5-HT plays critical and broad roles in normal animal physiology, including embryonic development, inflammation, cardiovascular, and gastrointestinal regulation, as well as the stimulation of a broad spectrum of behaviors^23–26^. By extension, aberrant levels of 5-HT, and/or disruption of its signaling pathways, are associated with many diseases, including digestive, cardiac, respiratory, and neuronal disorders^24,26,27^. In brain, 5-HT regulates neural activity via vesicular release from extended axonal projections originating in the dorsal raphe nucleus (DRN), where it acts on multiple G protein-coupled receptor families to regulate behavioral plasticity^23–26^.

In addition to receptor-mediated signaling, 5-HT can be conjugated to Gln residues within certain protein substrates via transamidation by the Ca^2+^-dependent transglutaminase 2 (TG2) enzyme^28,29^. While initially described in the context of cytosolic substrates, we discovered that these monoaminylation reactions additionally occur in the nucleus. For example, 5-HT (as well as dopamine^30–32^ and histamine^7^) can be covalently bound to histone H3 at Gln 5 (H3Q5) to establish H3Q5 serotonylation (H3Q5ser) on nucleosomes^1,21,33^. H3Q5ser can occur independently of neighboring posttranslational modifications (PTMs); however, this PTM often co-occurs with H3 Lys4 (K4) trimethylation (me3), establishing the dual H3K4me3Q5ser mark at transcriptional start sites (TSSs). The presence of H3K4me3Q5ser serves to potentiate transcription via: 1) increased binding of TAF3, a TFIID subunit, which aids in RNA Pol II-mediated transcription^1,20,21^; and 2) inhibition of H3K4 demethylases (KDM5 family and LSD1)^21^. Moreover, H3Q5ser has been shown to engage WDR5, a member of MLL and SETD1 H3K4 methyltransferase complexes, where it functions to stimulate deposition of H3K4 methylation^7,34^. H3 serotonylation levels have been observed to increase following post-mitotic differentiation of human induced pluripotent stem cells (hiPSCs) to 5-HTergic neurons, as well as in rat neuronal precursor cells from the 5-HTergic DRN (RN46A-B14 cells)^1^. Mutating H3Q5 to alanine (H3Q5A) in RN46A-B14 cells prevents H3 serotonylation, thereby reversing gene expression programs important for neural differentiation. In addition to its documented actions in 5-HTergic cells, the H3K4me3Q5ser mark has been detected *in vivo* in other monoaminergic and non-monoaminergic cells (both neurons and glia) of the brain^1,6^, with its non-monoaminergic distribution hypothesized to be regulated, at least in part, by the ubiquitously expressed high-capacity, low-affinity monoamine transporter OCT3/SLC22A3. In addition to its roles in neural development, H3Q5ser has been found to have important functions in regulating gene expression and cellular outputs in the context of sensory processing^6^, stress- and depression-related phenotypes^8^, placental gene regulation^9^, cancer progression^10–19^, and circadian rhythms^7^.

While H3Q5ser’s functions in the context of neighboring H3K4me3 are now well documented, it remains unclear whether H3Q5ser itself engages with dedicated – non-H3K4me3-associated – *reader* proteins to facilitate its functions in mammalian cells. Here, employing a series of proteomic-, structural-, molecular-, epigenomic-, and cellular-based assays, we now demonstrate that H3Q5ser is *read* by the Quinone reductase 2 (QR2) enzyme, both *in vitro* and *in vivo*, and that this interaction can occur in the context or absence of adjacent H3K4me3. Importantly, implementing CRISPR-Cas9-mediated gene editing to specifically disrupt QR2-H3Q5ser interactions without perturbing QR2’s endogenous enzymatic functions, we show that QR2 binding to H3Q5ser is critical for establishing appropriate gene expression programs in human neurons, which contribute to cellular maturation. These findings thus establish QR2 as a *bona fide reader* of H3Q5ser, an interaction that plays essential roles during human neurodevelopment, and one that may contribute to pathological states associated with aberrant 5-HTergic signaling in the brain and beyond.

## RESULTS

### Identification of QR2 as an H3Q5ser binding protein

**Extended Data Figure 1.**
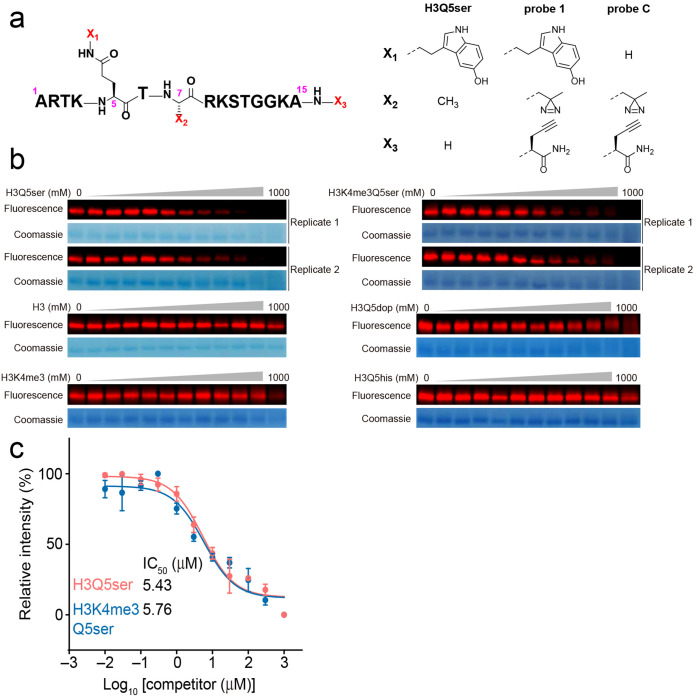
QR2 selectively binds to H3Q5ser (**a**) Chemical structures of H3Q5ser, probe 1, and probe C. (**b**) In-gel fluorescence scanning results of different H3 peptides competing with probe 1-induced labeling of recombinant QR2. (**c**) Determination of IC_50_ for inhibition of probe 1-induced labeling of QR2 by H3Q5ser and H3K4me3Q5ser peptides. The fluorescence intensity of each band was quantified by ImageJ. All curves were normalized between 100 and 0% at the highest and lowest fluorescence intensities, respectively. Data are reported as mean ± SD (*n* = 2). All immunoblotting experiments repeated 3X. See Supplementary Figure 1 for uncropped blots.

**Figure 1.**
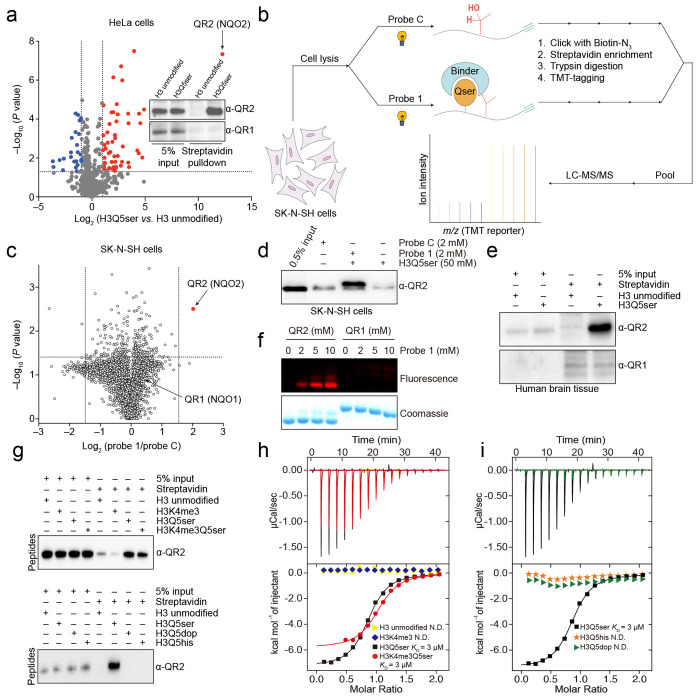
Identification and characterization of QR2 as an H3Q5ser binding proteins (**a**) Volcano plots of H3_1-18_Q5ser *vs.* H3_1-18_ unmodified binders from HeLa nuclear extracts identified by Streptavidin capture and LC-MS/MS (*n* = 4; −log_10_ *P* value < 0.05; Log_2_ fold difference > 1). Inset: immunoblotting validation of QR2, but not QR1, binding to H3Q5ser in HeLa cells. (**b**) Workflow of TMT-based chemical proteomic profiling of H3Q5ser binding proteins. (_c_) Volcano plot of proteins captured by serotonylated probe 1 *vs.* unmodified probe C from SK-N-SH cell lysates. Statistical analysis was conducted using an unpaired two-tailed Student’s *t*-test. Cutoffs: significance *P* < 0.05; TMT ratio Log**2** (probe 1/probe C) > 1.5 (*n* = 5). (**d**) Photo-crosslinking pulldown validation of QR2 binding to H3Q5ser in SK-N-SH cell lysates. (**e**) H3_1-18_Q5ser *vs.* H3_1-18_ unmodified peptide pulldowns from human brain nuclear extracts, followed by immunoblotting for QR2 or QR1. (f) Recombinant QR2, but not its paralog QR1, could be labeled by probe 1. (**g**) Peptide pulldowns with recombinant QR2, followed by immunoblotting for QR2. (**h**-**i**) Titration and fitting curves of peptides titrated into QR2. K_D_ values are provided. See Extended Data Table 1 for ITC statistics. All immunoblotting experiments repeated 3X. See Supplementary Figure 1 for uncropped blots.

To initially assess binding interactions between the H3Q5ser modification and nuclear proteins, we performed H3 N-terminal peptide pulldown assays using biotinylated H3_1-18_Q5ser *vs.* H3_1-18_ unmodified, followed by Streptavidin capture and mass spectrometry, to enrich for/identify proteins from HeLa nuclear extracts that display potentiated binding to the serotonyl mark. Following LC-MS/MS analysis, we observed that numerous proteins displayed either modestly attenuated or potentiated binding to H3Q5ser *vs.* H3 unmodified, some of which were consistent with previous studies from our group and others (e.g., attenuated – KDM5A/B^21^; potentiated – WDR5^7,34^). However, one protein – the Quinone reductase 2 enzyme (QR2; aka NQO2 or N-ribosyldihydronicotinamide: quinone dehydrogenase 2) – was found to display a considerably stronger interaction with the H3Q5ser peptide *vs*. other proteins identified in our analysis (Fig. 1a; Supplementary Data 1); note that QR2’s paralog, QR1, was not found to interact with the H3Q5ser peptide (Fig. 1a, **inset**), indicating that these two evolutionarily related proteins may have diverged to carry out distinct functions in cells. Next, given previously observed roles for H3Q5ser in guiding gene expression programs within neural cells specifically^1,8^, we developed an orthogonal photoaffinity-based chemical probe approach (probe 1; derived from an H3_1-15_Q5ser peptide in which the Ala7 residue was replaced by a diazirine-containing photoreactive amino acid; Extended Data Fig. 1a), to covalently capture H3Q5ser binding proteins upon UV irradiation in SK-N-SH cell lysates (a neuroblastoma cell line). This probe also introduced an alkyne-containing amino acid at its C-terminus to enable bioorthogonal conjugation of fluorescence tags (for visualization) or biotin (for isolation) to target proteins. An unmodified probe C (Extended Data Fig. 1a) was developed in tandem as a negative control. To further profile potential binding proteins of H3Q5ser, whole-cell lysates were extracted from SK-N-SH cells and were photo-crosslinked with probe 1 and probe C. Proteins captured by the probes were conjugated to biotin via click chemistry, followed by affinity purification and trypsin digestion. The resulting peptides were labeled with Tandem Mass Tag (TMT) reagents and pooled for subsequent quantitative mass spectrometry analysis (Fig. 1b). Similar to our results from HeLa cells, the QR2 enzyme, but not QR1, exclusively stood out with a high TMT ratio (Fig. 1c; Supplementary Data 2; validated via immunoblotting in Fig. 1d). Importantly, QR2’s binding to H3Q5ser was also validated via peptide pulldowns from human brain nuclear extracts (Fig. 1e), indicating that such interactions are preserved in endogenous brain tissues. We next verified that interactions between QR2 and H3Q5ser are specific, demonstrating that recombinant full-length QR2, but not QR1, can be robustly labeled by serotonylated probe 1, but not the unmodified probe C (Fig. 1f). We subsequently performed biotinylated peptide pulldown assays, followed by immunoblotting, to compare recombinant QR2’s binding to an unmodified H3 peptide *vs.* those containing H3K4me3, H3Q5ser, or H3K4me3Q5ser (all H3_1-18_). These results indicated that while QR2 does not bind to H3 unmodified or H3K4me3, it does bind to both H3Q5ser and H3K4me3Q5ser peptides with roughly equal affinity (Fig. 1g, **top**). Given that additional H3 monomainylation marks have also recently been identified, such as H3Q5 dopaminylation (dop)^30–32^ and histaminylation (his)^7^, we next compared QR2 binding to H3 peptides containing H3Q5ser *vs.* H3Q5dop *vs.* H3Q5his *vs.* H3 unmodified, which revealed that QR2 selectively interacts with H3Q5ser (Fig. 1g, **bottom**). To confirm the strength of QR2’s selective binding to H3Q5ser (in the presence or absence of adjacent H3K4me3), we performed isothermal titration calorimetry (ITC) to determine the K_D_ of these interactions. Consistent with our *in cellulo* and *in vitro* peptide pulldown assays, we observed that QR2 binds to H3Q5ser with moderately high affinity (3 μM), an interaction that was unaffected by the presence of H3K4me3 (H3K4me3Q5ser; 3 μM) (Fig. 1h; Extended Data Table 1). Importantly, our ITC results similarly indicated that QR2 does not bind H3 unmodified or H3K4me3 peptides (Fig. 1h), nor does it bind H3Q5dop or H3Q5his PTMs (Fig. 1i; Extended Data Table 1). Finally, using our orthogonal cross-linking approach, we observed that probe 1-induced labeling could be effectively competed off by H3Q5ser and H3K4me3Q5ser peptides, but not by H3 unmodified, H3K4me3, H3Q5dop, or H3Q5his (Extended Data Fig. 1b–c).

To date, QR2 has primarily been characterized as a cytosolic flavoprotein (with flavin adenine dinucleotide (FAD) as its coenzyme), which functions as a putative detoxification enzyme involved in the chemical reduction of reactive quinones^35^. Interestingly, QR2 has already been shown to bind free 5-HT (as well as melatonin), and these interactions appear to inhibit its reductase activities^36,37^. QR2 exists as a homodimer^38^, and while it is expressed across sub-cellular compartments, it currently has no known nuclear functions. Genetic variants of *NQO2* have been identified in humans, linking dysregulation of its function to tumorigenesis and neurodegenerative disorders^39^. Additionally, studies in *Nqo2* deficient mice have indicated roles for this protein in the regulation of cognitive behaviors^40^. While our data indicate that QR2 binds to H3Q5ser in neural cells, including in human brain tissues, whether such interactions contribute to previously observed neural phenotypes remains unknown.

### Molecular recognition of H3Q5ser by QR2

**Extended Data Figure 2.**
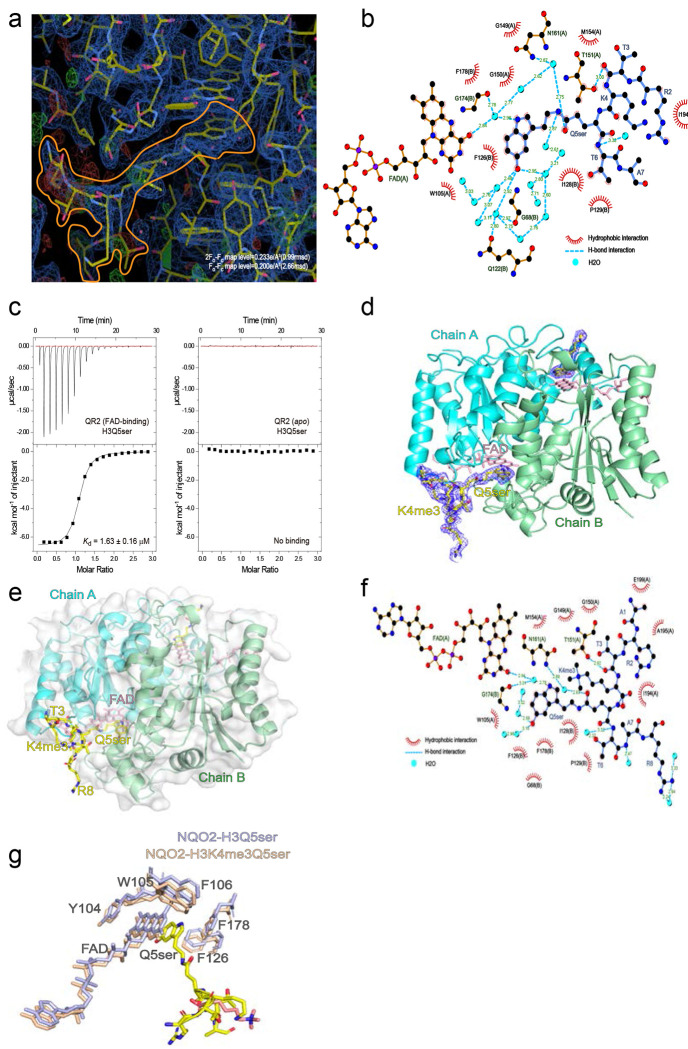
Common molecular recognition of H3Q5ser and H3K4me3Q5ser by QR2 (**a**) Corrected difference electron density map of the QR2-H3Q5ser crystal structure. The continuous density marked by the orange line corresponds to the H3Q5ser peptide, while the remaining regions represent the density of QR2. The electron density from the H3R2 to the H3A7 is visible. (**b**) LIGPLOT diagram listing critical contacts between the H3Q5ser peptide and QR2. The main chain of the peptide is shown in blue, surrounded by multiple hydrophobic interactions (red arcs) and several pairs of hydrogen bond interactions (blue lines). The distances of the hydrogen bond interactions are labeled in green text. Water molecules are indicated by cyan balls, and the FAD molecule is also displayed in the diagram. The letters A and B following the amino acid residues denote Chain A and Chain B in the QR2 dimer, respectively. Numbering of residues excludes the initiating methionine of QR2. (**c**) Titration and fitting curves of peptides titrated into wildtype and *apo* (depleted of FAD) QR2. KD values are provided. See Extended Data Table 1 for ITC statistics. (**d**) Structure of the QR2-H3K4me3Q5ser complex. The blue mesh shows the electron density of the H3K4me3Q5ser peptide. The Q5ser residue inserts into the binding pocket of QR2, while the K4me3 residue extends out from the QR2 surface and does not participate in recognition. (**e**) The QR2-H3K4me3Q5ser co-crystal structure reveals that Q5ser inserts into the interior of the molecule, while T3 and K4me3 are oriented toward the exterior and do not participate in recognition. (**f**) LIGPLOT diagram listing critical contacts between the H3K4me3Q5ser peptide and QR2. The main chain of the peptide is shown in blue, and K4me3 is visible in the diagram. Multiple hydrophobic interactions (red arcs) surround Q5ser, which are highly consistent with those observed in the NQO2-H3Q5ser complex. Numbering of residues excludes the initiating methionine of QR2. (**g**) Comparison of the conformations of key amino acids involved in recognition between the QR2-H3K4me3Q5ser co-crystal structure (pink) and the QR2-H3Q5ser co-crystal structure (purple). Their conformations are completely identical. The unmodified K4 is shown in yellow, while the K4me3 modification is shown in red. See Extended Data Table 2 for x-ray crystallography data collection and refinement statistics.

**Figure 2.**
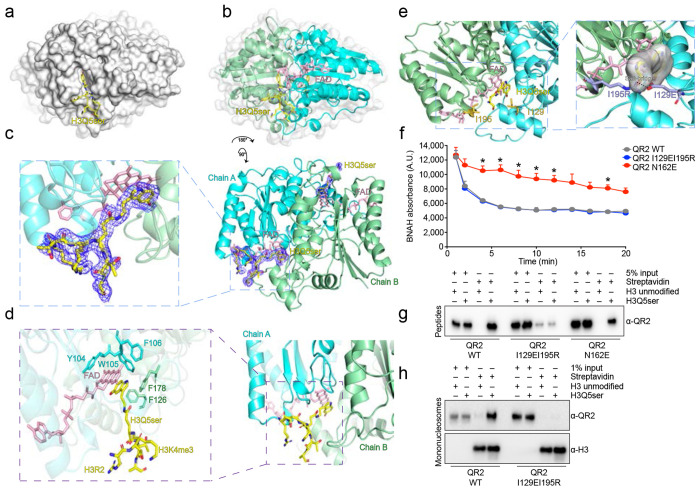
Molecular recognition of H3 Gln5 serotonylation by QR2 (**a**) Structure of the QR2-H3Q5ser complex. H3 residues are shown as yellow sticks, QR2 is shown as a white surface. The H3Q5ser residue inserts into the binding pocket of QR2. (**b**) Ribbon structure of the QR2-H3Q5ser complex. Ribbon shows detailed construction of QR2, H3 residues are shown as yellow sticks, coenzyme FAD is shown as pink sticks. (**c**) After rotating by a certain angle, as indicated in the diagram, the electron density of the H3Q5ser peptide is displayed, shown as a blue mesh. Chain A and Chain B in the QR2 dimer are respectively labeled. (**d**) Details of the QR2-H3Q5ser complex structure are shown. H3 residues are shown as yellow sticks, coenzyme FAD is shown as pink sticks. The key amino acid residues in Chain A (cyan) and Chain B (green) are labeled. Chain A Y104, FAD, and the serotonin ring form π-π stacking interactions, while Chain A W105, F106, Chain B F126, and F178 collectively form a hydrophobic aromatic cage, constituting the specific pocket for recognizing H3Q5ser. See Extended Data Table 2 for x-ray crystallography data collection and refinement statistics. (**e**) Molecular design of mutants to disrupt binding to H3Q5ser. The key amino acid residues I129 and I195 are labeled in orange. The mutants I129E and I195R are displayed as purple sticks. I129E and I195R are predicted to form a salt bridge via structural modeling, preventing the H3Q5ser peptide from inserting into the internal pocket of QR2. (**f**) BNAH enzymatic assay comparing wildtype *vs*. I129EI129R (H3Q5ser binding deficient) *vs*. N162E (catalytically deficient) QR2. *n* = 3. Two-way RM ANOVA [main effects: time (F_1.908_, _11.45_ = 107.2, *P*<0.001), QR2 mutation (F_2,6_ = 21.20, *P*=0.0019), and QR2 mutation x time (F_3.816_, _11.45_ = 4.884, *P*=0.0161); post hoc: Dunnett’s MC test (*p<0.05; QR2 N162E *vs*. wildtype)]. Data shown as mean ± SEM. (**g**) Peptide pulldown validations of mutant *vs*. WT QR2 abrogation of binding on H3Q5ser. (**h**) Mononucleosome pulldown validations of mutant *vs*. WT QR2 abrogation of binding on H3Q5ser. All immunoblotting experiments repeated 3X. See Supplementary Figure 1 for uncropped blots.

Given that QR2 binds to H3Q5ser directly, we next wished to elucidate the manner in which this molecular recognition occurs. To do so, we performed x-ray crystallography to capture the QR2-H3Q5ser complex containing QR2’s coenzyme FAD. These analyses demonstrated that H3Q5ser inserts within a binding pocket near the homodimerization interface of QR2’s chain A and chain B (Fig. 2a–b; Extended Data Table 2), with rotation of the complex revealing full electron density tracing of the H3Q5ser peptide from H3 Arg (R) 2 to H3 Ala (A) 7 (Fig. 2c; Extended Data Fig. 2a). Further zooming into this binding pocket, we were able to determine that Tyr (Y) 104 of QR2’s chain A, the indole ring of 5-HT, and FAD form π-π stacking interactions, while QR2’s chain A Trp (W) 105 and Phe (F) 106, along with QR2’s chain B F126 and F178, collectively form a hydrophobic aromatic cage to facilitate the specific binding pocket for H3Q5ser recognition (Fig. 2d). This binding requires the formation of critical contacts between the H3Q5ser peptide and QR2, with the main chain of the H3Q5ser peptide being surrounded by multiple hydrophobic interactions and several pairs of hydrogen bonds (Extended Data Fig. 2b). Of note, the *apo* form of QR2 lacking the co-factor FAD was observed to completely lose its H3Q5ser binding activity (Extended Data Fig. 2c), highlighting the FAD-dependent nature of H3Q5ser recognition by QR2. Importantly, given that QR2 is able to bind to H3Q5ser in both the absence and presence of H3K4me3, we next performed x-ray crystallography to capture the QR2-H3K4me3Q5ser complex, again containing QR2’s coenzyme FAD. These data demonstrated that QR2’s molecular recognition of the dual PTM follows a similar logic to its binding of H3Q5ser alone, with H3K4me3 projecting outward from the QR2-H3Q5ser binding pocket (Extended Data Fig. 2d–g; Extended Data Table 2); such findings provide further support to our previous observations that the presence of H3K4me3 in the context of H3Q5ser does not impact the strength of QR2’s interaction with the serotonyl mark.

**Extended Data Figure 3.**
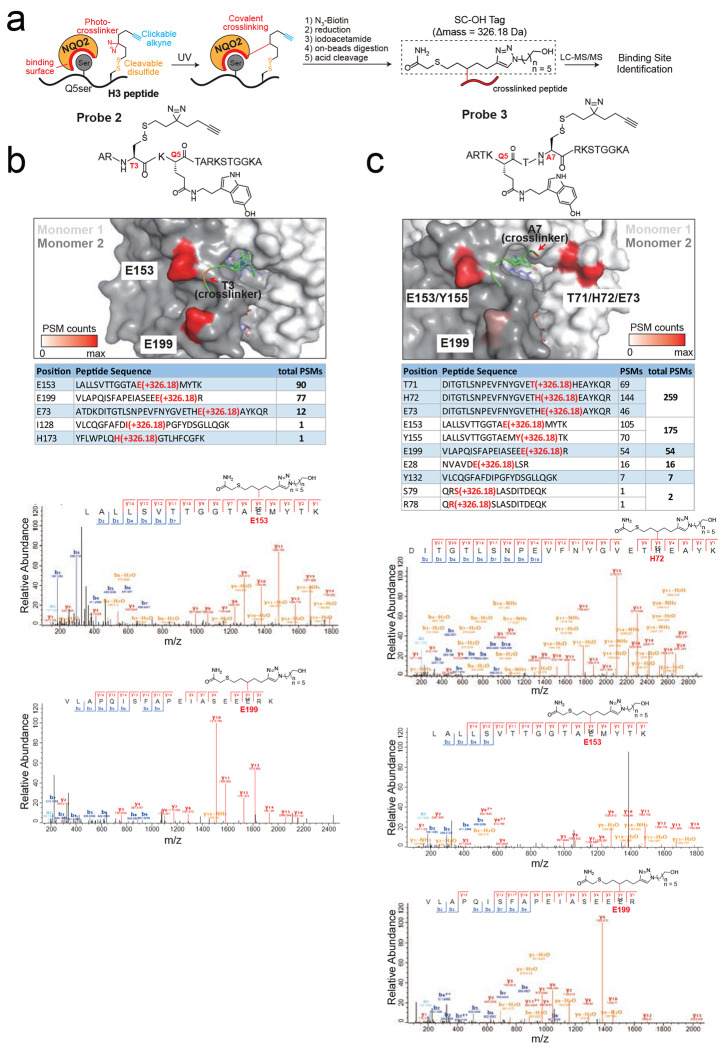
Photoaffinity probe-based mapping of the binding sites between QR2 and the H3Q5ser peptide (**a**) Schematic workflow for binding sites mapping between recombinant QR2 and H3Q5ser peptide. QR2 was incubated with photoaffinity probes carrying serotonylation at Q5 and a tri-functional amino acid ADdis-Cys that contains a diazirine, an alkyne, and a reductively cleavable disulfide bond. Following photo-crosslinking, proteins captured by the probe will be conjugated with biotin-azide carrying an acid-labile dialkoxydiphenylsilane (DADPS) linker and then immobilized by streptavidincoated beads. After reductive cleavage, iodoacetamide-mediated thiol capping, on-bead digestion, and washing steps, the peptides remaining on the beads should be those directly cross-linked to ADdis-Cys with binding sites information. A final acid treatment released the peptides with SC-OH tags (+326.18 Da) installed at the cross-linked residues that will be identifiable by LC-MS/MS analysis. (**b**) Crosslinking sites identified with probe 2 (ADdis-Cys incorporated at T3). Upper: chemical structure of probe 2. Middle: QR2 surface representation highlighting major crosslinked residues (E153, E199) color-coded by peptide–spectrum match (PSM) counts. Lower: summary table listing crosslinked positions, corresponding peptide sequences, and total PSMs; representative MS/MS spectra for E153 and E199 with annotated band y-type ions and marked modification sites. (**c**) Crosslinking sites identified with probe 3 (ADdis-Cys incorporated at A7). Upper: chemical structure of Probe 3. Middle: QR2 surface representation highlighting major crosslinked residues (T71, H72, E73, E153, Y155, E199) color-coded by peptide–spectrum match (PSM) counts. Lower: summary table listing crosslinked positions, corresponding peptide sequences, and total PSMs; representative MS/MS spectra for for T71/H72/E73, E153, and E199 with annotated b- and y-type ions and marked modification sites.

**Extended Data Figure 4.**
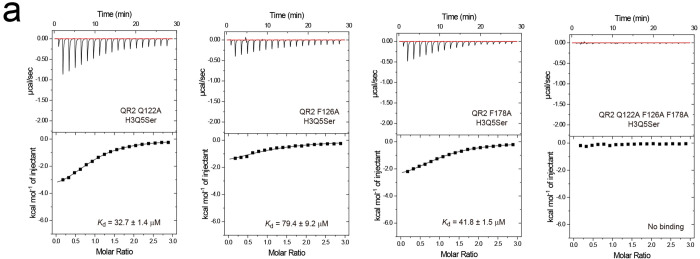
Mutational ITC validations of QR2’s molecular recognition of H3Q5ser (**a**) Titration and fitting curves of peptides titrated into mutant QR2. KD values are provided. See Extended Data Table 1 for ITC statistics.

**Extended Data Figure 5.**
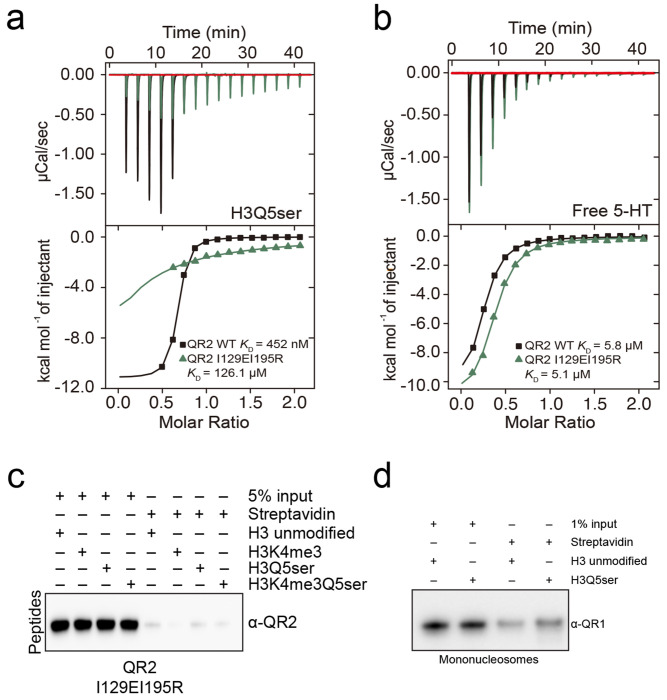
Validations of disruption of H3(K4me3)Q5ser recognition by QR2 I129EI195R mutations (**a**) Titration and fitting curves of H3Q5ser peptides titrated into QR2 WT *vs.* mutant. *K*_D_ values are provided. The mutants I129EI195R disrupts the recognition of QR2 to H3Q5ser peptides; (**b**) however, these mutations do not impact QR2’s binding to free 5-HT. See Extended Data Table 1 for ITC stats. (**c**) Peptide pulldowns with mutant recombinant QR2, followed by immunoblotting for QR2. (**d**) Mononucleosome pulldown validations of a lack of binding between QR1 and H3Q5ser. All immunoblotting experiments repeated 3X. See Supplementary Figure 1 for uncropped blots.

To provide orthogonal evidence for how QR2 interacts with the H3Q5ser peptide, we additionally mapped peptide binding sites on the protein surface using our photo-crosslinking strategy^41^ (Extended Data Fig. 3a–c), which revealed major crosslinks at residues located around the Q5ser-binding pocket of QR2 when mapped back to our co-crystal structure (see Extended Data Fig. 4a and Extended Data Table 1 for further ITC validations of QR2’s molecular recognition of H3Q5ser). Given that such photo-crosslinking was performed in solution, where both the protein and peptide exist in a freely moving state, these binding site mapping results complement and further support our crystallographic study,

Finally, to determine whether unique interactions observed in the QR2-H3Q5ser binding pocket are indeed critical for QR2’s molecular recognition of the mark, we focused our attention on two specific residues that contribute to hydrophobic interactions when QR2 is bound to H3Q5ser, Ile (I) 129 and I195. To disrupt QR2 binding to H3Q5ser, we next generated mutations in QR2 (I129EI195R) that were predicted – via structural modeling – to abrogate binding through the introduction of a salt bridge (Fig. 2e). These mutations were found to have no impact on QR2’s endogenous enzymatic functions (*vs.* QR2 Asn (N) 162 to E); Fig. 2f), while fully attenuating QR2’s binding to H3Q5ser, both in the context of peptides (Fig. 2g; Extended Data Fig. 5a,c; Extended Data Table 1) and mononucleosomes (Fig. 2h; Extended Data Fig. 5d). Given that QR2 has also been reported to bind free 5-HT, we additionally performed ITC assessments to explore the impact of QR2 I129EI915R mutations on its binding to free 5-HT, where we observed negligible effects (Extended Data Fig. 5b).

### QR2 binding to H3Q5ser contributes to cellular gene expression across species and cell-types

**Extended Data Figure 6.**
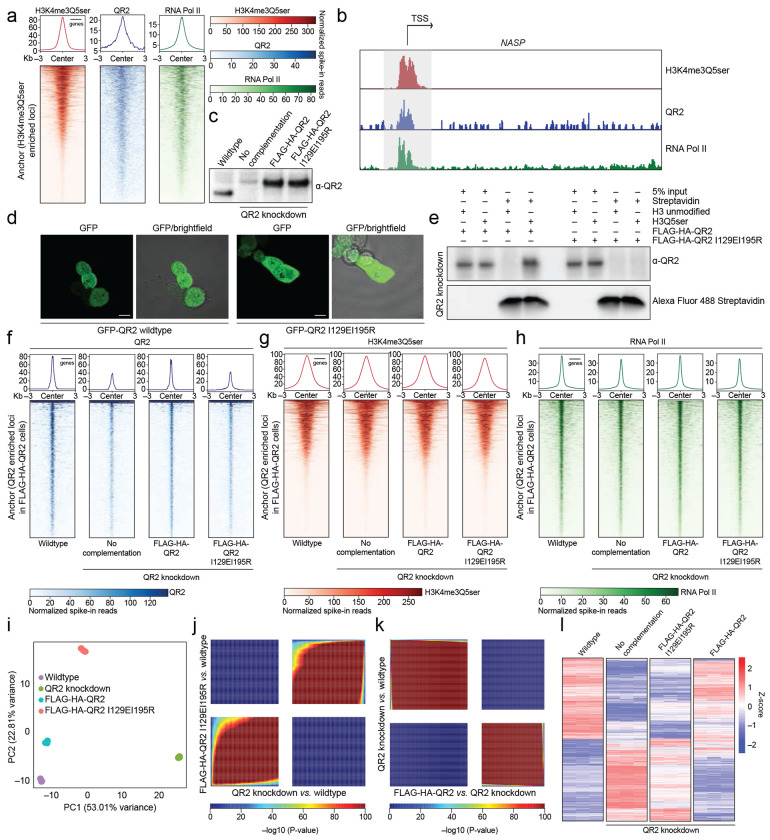
QR2 and H3Q5ser genomically co-enrich in HeLa cells to regulate gene expression. CUT&RUN-seq heatmaps of H3K4me3Q5ser, QR2, and RNA Pol II enrichment in (**a**) wildtype HeLa cells. (**b**) IGV tracks for H3K4me3Q5ser, QR2, and RNA Pol II at the *NASP* locus in wildtype HeLa cells. (**c**) Immunoblotting validation of endogenous QR2 knockdown in HeLa cells −/+ complementation with FLAG-HA-tagged wildtype *vs.* I129I195R QR2. (**d**) ICC/IF validation of QR2 subcellular localization in FLAG-HA-tagged wildtype *vs.* I129I195R QR2 complementation HeLa cells (*NQO2* knockdown background). Scale = 10 μm. (**e**) H3_1-18_Q5ser *vs.* H3_1-18_ unmodified peptide pulldowns from FLAG-HA-tagged wildtype *vs.* I129I195R QR2 complementation HeLa cells (*NQO2* knockdown background). CUT&RUN-seq heatmaps of (**f**) QR2, (**g**) H3K4me3Q5ser, and (**h**) RNA Pol II enrichment in wildtype, QR2 knockdown (no complementation), and FLAG-HA-tagged wildtype *vs.* I129I195R QR2 complementation HeLa cells (*NQO2* knockdown background). *n* = 3 for all. (**i**) RNA-seq-based PCA analysis of wildtype, QR2 knockdown (no complementation), and FLAG-HA-tagged wildtype *vs.* I129I195R QR2 complementation HeLa cells (*n* = 3 for all). RRHO analyses comparing gene expression patterns (RNA-seq) between (**j**) QR2 knockdown *vs.* wildtype and I129I195R QR2 complementation HeLa cells (*NQO2* knockdown background), as well as (**k**) QR2 knockdown *vs.* wildtype and QR2 wildtype complementation HeLa cells (*NQO2* knockdown background). (**l**) Heatmap of DEGs identified in QR2 knockdown *vs.* wildtype HeLa cells (anchor) across all four HeLa cell lines. All immunoblotting experiments repeated 3X. See Supplementary Figure 1 for uncropped blots.

Since our structural analyses indicated that specific hydrophobic interactions within the QR2-H3Q5ser binding pocket can be perturbed to prevent QR2-H3Q5ser recognition without impacting other aspects of QR2’s functions, we next aimed to exploit these QR2 I129EI195R mutations in cells to explore potential roles for QR2-H3Q5ser binding in the regulation of mammalian gene expression. To do so, we first examined whether QR2 co-enriches with H3(K4me3)Q5ser genome-wide in HeLa cells, a simple genetically modifiable tissue culture system, which has previously been shown to contain the H3 serotonyl mark owing to the presence of 5-HT supplemented to the media via serum^33^. We performed CUT&RUN-seq^42^ using antibodies recognizing H3K4me3Q5ser (validated previously^1^), QR2, and RNA Pol II, which demonstrated that QR2 co-enriches with H3K4me3Q5ser genome-wide, largely at permissive genes displaying co-enrichment with RNA Pol II (Extended Data Fig. 6a–b; Supplementary Data 3). Given such co-enrichment, we next performed CRISPR-Cas9 editing to knockdown endogenous QR2 in HeLa cells, followed by genetic complementation (under the endogenous *NQO2* promoter) with either FLAG-HA-tagged QR2 wildtype or QR2 I129EI195R (Extended Data Fig. 6c); note that complementation with the mutant form of QR2 did not have any impact on QR2’s subcellular localization, which is both cytoplasmic and nuclear (Extended Data Fig. 6d). Importantly, employing H3_1-18_Q5ser *vs.* H3_1-18_ unmodified peptide pulldowns in HeLa nuclear extracts from wildtype *vs.* mutant complemented cells, we first verified that our approach worked, demonstrating that biotinylated H3Q5ser peptides efficiently pulldown wildtype, but not mutant, QR2 in knockdown/complemented cells (Extended Data Fig. 6e). Next, we performed CUT&RUN-seq for H3K4me3Q5ser, QR2, and RNA Pol II in wildtype *vs.* QR2 knockdown (no complementation) *vs.* FLAG-HA-QR2 *vs.* FLAG-HA-QR2 I129EI195R complemented cells, demonstrating that QR2 knockdown results in reduced co-enrichment of QR2 at H3K4me3Q5ser/RNA Pol II positive loci, an effect that was fully rescued with wildtype, but not mutant, QR2 complementation (Extended Data Fig. 6f–h; Supplementary Data 4). Finally, to assess whether QR2’s binding to H3Q5ser plays functional roles in guiding gene expression in HeLa cells, we performed RNA-seq in wildtype *vs.* QR2 knockdown (no complementation) *vs.* QR2 (wildtype or mutant) complementation cells, which demonstrated that the vast majority of genes displaying differential expression between QR2 knockdown *vs.* wildtype cells were efficiently rescued by complementation with wildtype, but not mutant, QR2 (Extended Data Fig; 6i–l, Supplementary Data 5).

**Extended Data Figure 7.**
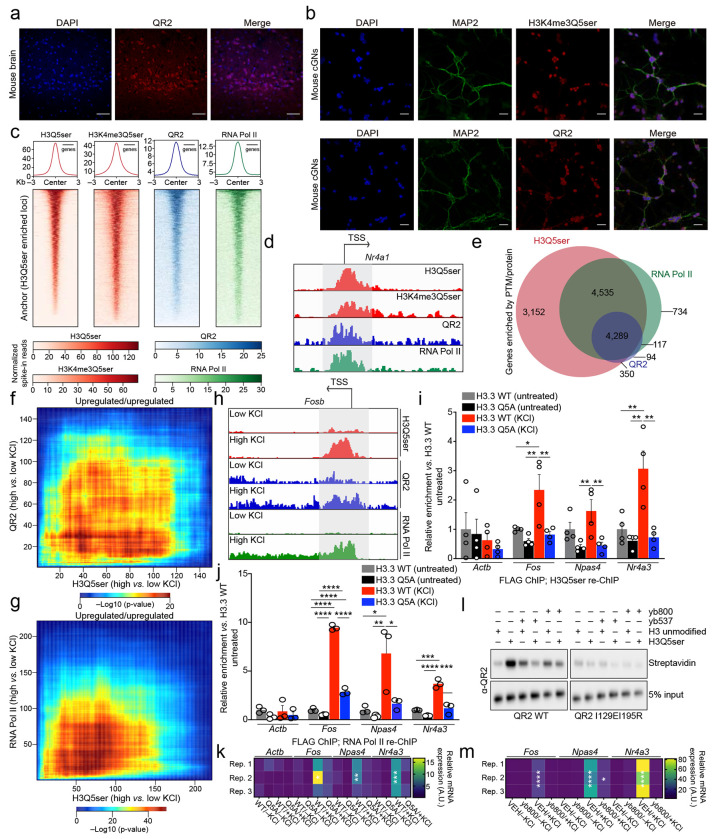
QR2 and H3Q5ser genomically co-enrich in rodent neurons and correlate with genes involved in synaptic plasticity. (**a**) IHC/IF validation of QR2 subcellular localization in wildtype mouse brain. Scale = 50 μm. (**b**) ICC/IF validation of QR2 subcellular localization in wildtype mouse cGNs. Scale = 20 μm. ChIP-seq heatmaps of H3(K4me3)Q5ser, QR2, and RNA Pol II enrichment in (**c**) wildtype mouse cGNs. (**d**) IGV tracks for H3Q5ser, QR2, and RNA Pol II at the *Nr4a1* locus in wildtype mouse cGNs. (**e**) Venn diagram of gene displaying overlapping enrichment for H3Q5ser, QR2, and RNA Pol II in wildtype mouse cGNs. RRHO analyses comparing (**f**) H3Q5ser enrichment in wildtype mouse cGNs −/+ KCl *vs.* QR2 enrichment in wildtype mouse cGNs −/+ KCl, and (**g**) H3Q5ser enrichment in wildtype mouse cGNs −/+ KCl *vs.* RNA Pol II enrichment in wildtype mouse cGNs −/+ KCl. (**h**) IGV tracks for H3Q5ser, QR2, and RNA Pol II at the *Fosb* locus in wildtype mouse cGNs −/+ KCl. (**i**) FLAG ChIP-/H3Q5ser re-ChIP-qPCRs following transduction of wildtype mouse cGNs −/+ KCl with H3.3 WT-FLAG *vs.* H3.3Q5A-FLAG vectors (*n* = 4; Two-way ANOVA, *P*<0.05 for effect of KCl, virus, or KCl x virus; post hoc Tukey’s MC tests – ** *P*<0.01, * *P*<0.05). (**j**) FLAG ChIP-/RNA Pol II re-ChIP-qPCRs following transduction of wildtype mouse cGNs −/+ KCl with H3.3 WT-FLAG *vs.* H3.3Q5A-FLAG vectors (*n* = 3; Two-way ANOVA, *P*<0.05 for effect of KCl, virus, or KCl x virus; post hoc Tukey’s MC tests – **** *P*<0.0001, *** *P*<0.001, ** *P*<0.01, * *P*<0.05). (**k**) qPCRs following transduction of wildtype mouse cGNs −/+ KCl with H3.3 WT-FLAG *vs.* H3.3Q5A-FLAG vectors [*n* = 3; Two-way ANOVA, *P*<0.05 for effect of KCl, virus, or KCl x virus; post hoc Dunnett’s MC tests (compared to –KCl H3.3 WT) – *** *P*<0.001, ** *P*<0.01). (**l**) H3_1-18_Q5ser *vs.* H3_1-18_ unmodified peptide pulldowns against recombinant QR2 in the presence or absence of QR2 inhibitors yb800 and yb537. (**m**) qPCRs following treatment of wildtype mouse cGNs −/+ KCl with vehicle *vs.* the QR2i yb800 [*n* = 3; Two-way ANOVA, *P*<0.05 for effect of KCl, QR2i, or KCl x QR2i; post hoc Uncorrected Fisher’s LSD (compared to –KCl vehicle) – **** *P*<0.0001, * *P*<0.05). Data shown as mean ± SEM. All immunoblotting experiments repeated 3X. See Supplementary Figure 1 for uncropped blots.

To ensure that QR2-H3Q5ser interactions are indeed conserved across species and/or cell-types, we next assessed QR2’s subcellular localization in mouse brain (Extended Data Fig. 7a) and primary neurons (mouse cerebellar granule neurons (cGNs); Extended Data Fig. 7b), where we found that – as in HeLa cells – QR2 is distributed throughout both cytoplasmic and nuclear compartments. CUT&RUN-seq for H3(K4me3)Q5ser, QR2, and RNA Pol II in cGNs further revealed that QR2 largely co-localizes with H3(K4me3)Q5ser genome-wide at permissive genes co-enriched by RNA Pol II (Extended Data Fig. 7c–e; Supplementary Data 6). We additionally found that loci determined to be activity-dependent in response to KCl-mediated neuronal depolarization displayed concordant regulation (primarily induction) of H3Q5ser, QR2, and RNA Pol II enrichment (Extended Data Fig. 7f–h; Supplementary Data 6). To further assess whether activity-dependent regulation of H3Q5ser-QR2 co-enrichment plays causal roles in the regulation of depolarization-induced gene expression (focused here on immediate early genes (IEGs)), we performed two complementary sets of analyses. (1) following transduction of cGNs with lentiviruses expressing either FLAG-HA-tagged H3.3 WT or H3.3 Q5A ± KCl (note that since neurons can only actively incorporate replication-independent H3.3 into postmitotic chromatin^43^, expression of H3.3Q5A effectively functions as a dominant negative to reduce levels of H3Q5 monoaminylations^1,7,8,30^), we performed chromatin immunoprecipitations (ChIPs) to enrich for exogenously expressed H3.3 (wildtype or mutant), followed by re-ChIPs for either H3Q5ser or RNA Pol II. In doing so, we found that while depolarization resulted in significantly increased enrichment of both H3Q5ser and RNA Pol II at IEG promoters in H3.3 wildtype-expressing cells, such enrichment was lost following transduction with the H3.3Q5A mutant (Extended Data Fig. 7i–j); these reductions in H3Q5ser and RNA Pol II in the mutant expressing cells similarly corresponded to a loss of IEG expression (Extended Data Fig. 7k). (2) To examine whether disrupting QR2 binding to H3Q5ser similarly results in a loss of KCl-mediated IEG induction, we employed recently described QR2 inhibitors, yb800 and yb537, which have been shown to bind QR2 within the QR2-H3Q5ser binding pocket near QR2 I129^44^. Both QR2i inhibitors were found to reduce QR2 binding to H3Q5ser *in vitro* (Extended Data Fig. 7l), and yb800 was found to significantly reduce the induction of IEGs in cGNs following depolarization (Extended Data Fig. 7m).

### QR2-H3Q5ser binding in human neurons is required for maintaining gene expression programs that guide cellular differentiation and maturation

**Extended Data Figure 8.**
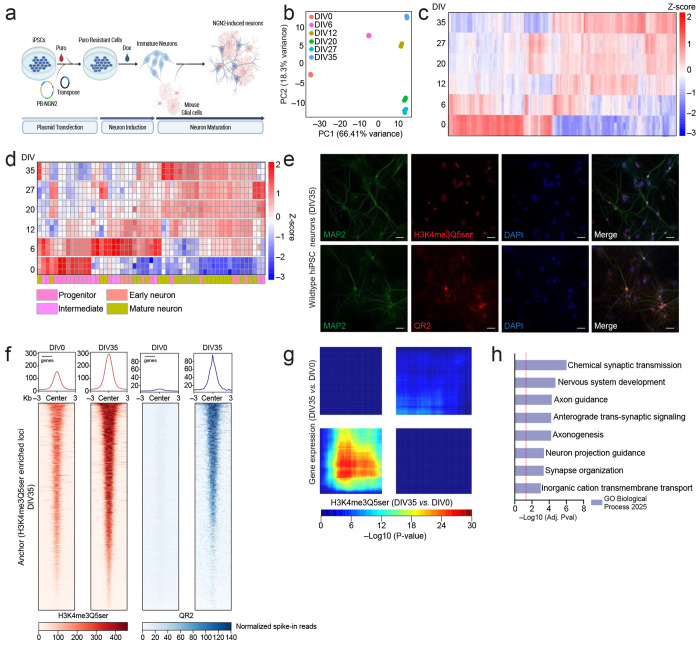
QR2-H3Q5ser dynamics in response to human neuronal differentiation and maturation (**a**) Cartoon of NGN2-mediated induction of hiPSC neurons (images generated using Biorender). (**b**) RNA-seq-based PCA analysis of hiPSC neurons during differentiation and maturation from DIV0 to DIV 35. *n* = 3 for all. (**c**) Heatmaps comparing differential gene expression (anchor DIV35 *vs.* DIV0) across hiPSC neuronal differentiation and maturation. (**d**) Heatmaps comparing differential expression of marker genes (columns) for neural progenitors, early neurons, intermediate neurons, and fully matured neurons (anchor DIV35 *vs.* DIV0) across hiPSC neuronal differentiation and maturation. (**e**) ICC/IF validation of QR2 subcellular localization in wildtype hiPSC neurons at DIV35. Scale = 50 μm. (**f**) CUT&RUN-seq heatmaps of H3K4me3Q5ser and QR2 enrichment in hiPSC neurons at DIV0 *vs.* DIV35. *n* = 3 for all. (**g**) RRHO analysis comparing DEGs at DIV35 *vs.* DIV0 to H3K4me3Q5ser enrichment at DIV35 *vs.* DIV0 in hiPSC neurons. (**h**) Go Biological Process 2025 enrichment for genes displaying concordant induction of H3K4me3Q5ser and gene expression across hiPSC neuronal differentiation (FDR<0.05).

Based on our findings that QR2 binding to H3Q5ser appears critical to cellular gene expression programs in both HeLa cells and cGNs (including in the context of neuronal activity-dependent transcription), we next wished to explore whether these interactions play causal roles in guiding gene expression programs in human neurons contributing to their maturation. We had shown previously that H3Q5ser is robustly induced in response to human 5-HTergic neuronal differentiation, and that disrupting H3Q5ser levels in immortalized 5-HTergic rat cells results in differentiation deficts^1^; however, we had yet to conclusively demonstrate whether the serotonyl mark, or its *reader* interactions, causally contribute to human neurodevelopmental processes. As such, and given that H3Q5ser has been found to be widely distributed across neuronal cell-types in brain^1^, we turned to a rapid single-step human neuronal induction system in which hiPSCs expressing the transcription factor Neurogenin-2 (NGN2) are efficiently differentiated into excitatory neurons^45^ (Extended Data Fig. 8a). NGN2-induced glutamatergic neurons can be co-cultured with mouse astrocytes, which allows for induction of robust neuronal differentiation/maturation gene expression programs over the course of only 35 days *in vitro* (DIV) (Extended Data Fig. 8b–d; Supplementary Data 7). Supplementation with 5-HT-containing serum was sufficient to sustain H3 serotonylation, and this maturation trajectory was accompanied by progressive co-enrichment for H3K4me3Q5ser and QR2 from DIV0 to DIV35 (Extended Data Fig. 8e–h; Supplementary Data 8–9).

**Extended Data Figure 9.**
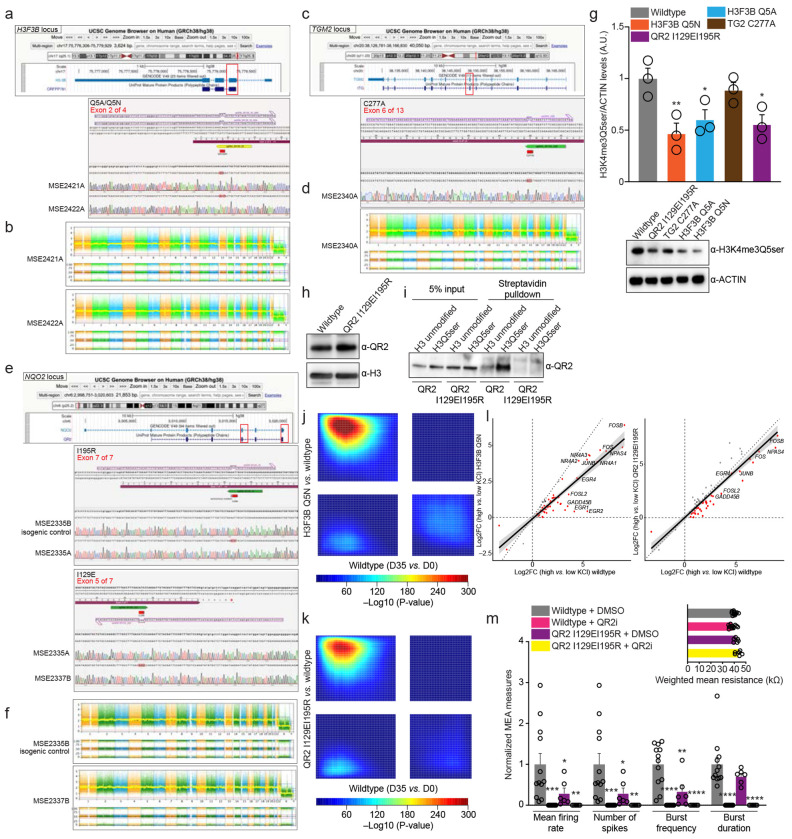
Generation and validation of mutant hiPSC lines (**a**) Overview of the *H3F3B* genomic locus and Sanger sequencing confirming precise introduction of the Q5A and Q5N missense substitutions. Sequences of the sgRNA and the ssODN used to mediate homology-directed repair (HDR) are shown. Traces from the edited lines (MSE2421A and MSE2422A) are shown together with their isogenic controls, with edited nucleotides highlighted. (**b**) Whole-genome copy-number and allele-frequency plots indicating that both knock-in lines (MSE2421A and MSE2422A) maintain normal karyotypic profiles following genome engineering. (**c**) Overview of the *TGM2* genomic locus and Sanger sequencing confirming precise introduction of the C277A missense substitution in MSE2340A. The edited nucleotide is highlighted within the exon 6 sequence. (**d**) Whole-genome copy-number and allele-frequency plots indicating that the knock-in line (MSE2340A) maintains a normal karyotypic profile following genome engineering. (**e**) Overview of the *NQO2* genomic locus and Sanger sequencing confirming precise introduction of the I195R and I129E missense substitutions. Traces from edited lines are shown together with their isogenic controls, with edited nucleotides highlighted. (**f**) Whole-genome copy-number and allele-frequency plots indicating that both the knock-in line (MSE2337B) and its control maintain normal karyotypic profiles following genome engineering. (**g**) Immunoblotting validation of the impact of hiPSC mutations *vs.* wildtype on H3K4me3Q5ser levels in mature neurons (DIV35). *n* = 3/line. One-way ANOVA (F_4,10_ = 6.145, p=0.0092); post hoc Dunnett’s MC test – H3F3B Q5N (**P=0.0074), H3F3B Q5A (*P=0.0393), and QR2 I129EI195R (*P=0.0220). ACTIN was used as a loading control. (**h**) Immunoblotting validation that the QR2 I129EI195R mutations do not impact QR2 expression. (**i**) H3_1-18_Q5ser *vs.* H3_1-18_ unmodified peptide pulldowns from wildtype *vs.* QR2 I129EI195R hiPSC neurons at DIV35. RRHO analyses (RNA-seq) comparing the transcriptional impact of (**j**) H3F3B Q5N or (**k**) QR2 I129EI195R mutations in hiPSC neurons to normal patterns of gene expression during neuronal maturation from DIV0 to DIV35. (**l**) Plots of activity-dependent (KCl-induced) gene expression in wildtype hiPSC neurons (dashed line) *vs.* activity-dependent (KCl-induced) gene expression in H3F3B Q5N or QR2 I129EI195R lines, indicating attenuated responses in the mutants. (**m**) MEA-based quantification of: normalized mean firing rate [two-way ANOVA, main effect of QR2i: F_1,32_ = 10.79, *P*=0.0025; Dunnett’s MC test – *** *P*<0.001, ** *P*<0.01, * *P*<0.05]; normalized spike number [two-way ANOVA, main effect of QR2i: F_1,32_ = 10.79, *P*=0.0025; post hoc: Dunnett’s MC test – *** *P*<0.001, ** *P*<0.01, * *P*<0.05]; normalized burst frequency [two-way ANOVA, main effects of QR2i (F_1,32_ = 31.01, *P*<0.0001), mutation (F_1_,32 = 7.615, *P*=0.0095) and QR2i x mutation (F1,32 = 7.615, *P*=0.0095); post hoc: Dunnett’s MC test – **** *P*<0.0001, ** *P*<0.01]; and normalized burst duration [two-way ANOVA, main effect of QR2i: F_1,32_ = 46.96, *P*<0.0001; post hoc: Dunnett’s MC test – **** *P*<0.0001] in hiPSC wildtype (*n* = 12) or QR2 I129EI195R (*n* = 6) neurons −/+ the QR2i yb800. Inset: weighted mean resistance for each condition indicating no impact of genotype/treatment on neuronal viability.

Using CRISPR-Cas9-mediated gene editing in the parental hiPSC line WTC11^46^, we generated multiple homozygous knock-in clones targeting the *NQO2* (QR2 I129EI195R; disrupting H3Q5ser binding while preserving enzymatic functions), *TGM2* (C277A; catalytically inactivating its monoaminylase functions), and *H3F3B* (Q5A and Q5N; rendering ~50% of H3 in post-mitotic neurons incapable of serotonylation). These lines enabled us to directly assess roles for H3Q5ser and QR2-H3Q5ser interactions during human neuronal development. Although both *H3F3A* and *H3F3B* encode the H3.3 variant in humans note that while two genes – *H3F3A* and *H3F3B* – encode for the H3.3 variant in humans, we selectively targeted *H3F3B* to achieve partial disruption of serotonylation while preserving neuronal viability^47^. Correct targeting was confirmed by Sanger sequencing (Extended Data Fig. 9a–f). In differentiated neurons at DIV35, H3.3 Q5A and Q5N mutations resulted in significantly reduced levels of H3K4me3Q5ser relative to wildtype isogenic controls, with similar attenuation observed in QR2 I129EI195R mutant neurons (Extended Data Fig. 9g), suggesting that QR2 binding to H3Q5ser may help to stabilize the mark against TG2-mediated erasure. Notably, the QR2 mutation did not alter overall protein expression levels in differentiated neurons (Extended Data Fig. 9h). Peptide pulldown assays using H3_1-18_Q5ser *vs.* H3_1-18_ unmodified peptides further demonstrated robust binding of wildtype, but not mutant, QR2 to the H3 serotonylation mark (Extended Data Fig. 9i).

**Figure 3.**
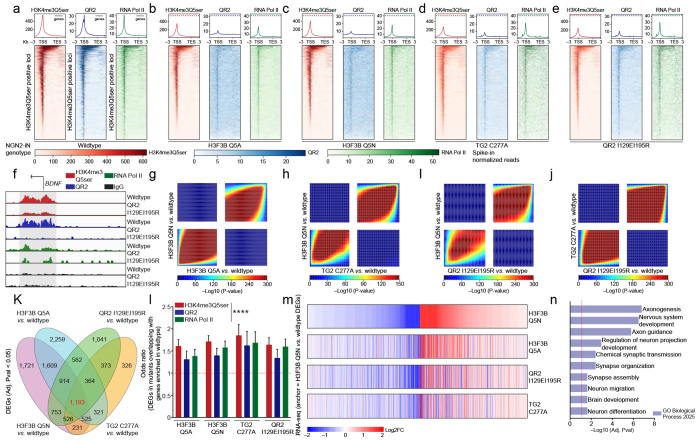
QR2-H3Q5ser interactions contribute to human neurodevelopmental gene expression CUT&RUN-seq heatmaps of H3K4me3Q5ser, QR2, and RNA Pol II enrichment in (**a**) wildtype, (**b**) H3F3BQ5A, (**c**) H3F3BQ5N, (**d**) TG2 C277A, and (**e**) QR2 I129RI195R hiPSC neurons at DIV 35 (*n* = 3 for all; peak enrichment (spike-in normalized read coverage) anchored on wildtype signal for each line). For **b**-**e**, dashed lines represent wildtype signal. (**f**) IGV tracks for H3K4me3Q5ser, QR2, and RNA Pol II at the *BDNF* locus in wildtype *vs*. QR2 I129EI195R hiPSC neurons. For each antibody, scales are consistent between wildtype and QR2 mutants. RRHO analyses comparing gene expression patterns (RNA-seq) between (**g**) H3F3B Q5A *vs*. wildtype and H3F3B Q5N *vs*. wildtype, (**h**) H3F3B Q5N *vs*. wildtype and TG2 C277A *vs*. wildtype, (**i**) H3F3B Q5N *vs*. wildtype and QR2 I129EI195R *vs*. wildtype, and (**j**) TG2 C277A *vs*. wildtype and QR2 I129EI195R *vs*. wildtype demonstrating concordance across mutant lines (*n* = 3 for all). (**k**) Venn diagram displaying the number of overlapping differentially expressed genes (DEGs) between wildtype and mutant hiPSC neurons (FDR < 0.05). (**l**) Bar plot of odds ratio analysis (Fisher’s exact text) examining the overlap of DEGs across mutant lines *vs*. wildtype and enrichment of H3K4me3Q5ser, QR2, and RNA Pol II in wildtype neurons at these same genes (****P<0.0001). (**m**) Heatmaps of genes identified as being differentially expressed in H3F3B Q5N *vs*. wildtype neurons (anchor; FDR < 0.05) across all mutant lines. (**n**) Go Biological Process 2025 enrichment for the 1,193 overlapping DEGs identified in panel **k** (FDR<0.05).

Employing CUT&RUN-seq, we then profiled enrichment patterns for H3K4me3Q5ser, QR2, and RNA Pol II across wildtype *vs.* mutant NGN2-induced neurons at DIV35. Similar to that observed in HeLa cells and mouse cGNs, we found that QR2 displays robust co-enrichment with H3K4me3Q5ser at permissive loci (co-enriched for RNA Pol II) in wildtype human neurons (Fig. 3a, f; Supplementary Data 7); however, this co-enrichment was severely attenuated across all four mutant lines (Fig. 3b–f; Supplementary Data 10), indicating that both direct (Q5A and Q5N) and indirect manipulations of the mark or its binding interactions with QR2 (QR2 I129EI195R and TG2 C277A) are sufficient to disrupt H3Q5ser-QR2 co-enrichment genome-wide. In addition, consistent with our findings in mouse cGNs, we observed that such perturbations are also sufficient to reduce RNA Pol II localization at H3K4me3Q5ser-marked loci. Corresponding RNA-seq analyses of wildtype *vs.* mutant lines revealed that both direct and indirect manipulations of the mark or its binding interactions with QR2 similarly result in dysregulated patterns of gene expression in maturing neurons (Fig. 3g–j; Extended Data Fig. 9j–k; Supplementary Data 7, 11). Indeed, differential expression analysis identified 1,193 protein-coding genes displaying overlapping and consistent dysregulation across the four mutant lines (Fig. 3k–m; Supplementary Data 12), with these overlapping genes found to be enriched for gene ontologies related to neuronal maturation (e.g., axonogenesis, nervous system development, synapse organization, and so on; Fig. 3n; Supplementary Data 13). Finally, since both disruption of H3Q5ser levels (H3.3 Q5A *vs.* WT transduction) and inhibition of QR2 binding to H3Q5ser (yb800 *vs.* vehicle) were found to disrupt activity-dependent gene expression in mouse cGNs, we next assessed the potential impact of perturbing H3Q5ser (Q5N) or QR2 binding to H3Q5ser (QR2 I129EI195R) in differentiated human neurons in response to KCl-mediated depolarization. Consistent with our studies in mouse cGNs, we observed that both manipulations were sufficient to attenuate activity-dependent gene regulation in NGN-induced neurons (Extended Data Fig. 9l).

### QR2-H3Q5ser interactions are required for human neuronal physiological maturation

**Figure 4.**
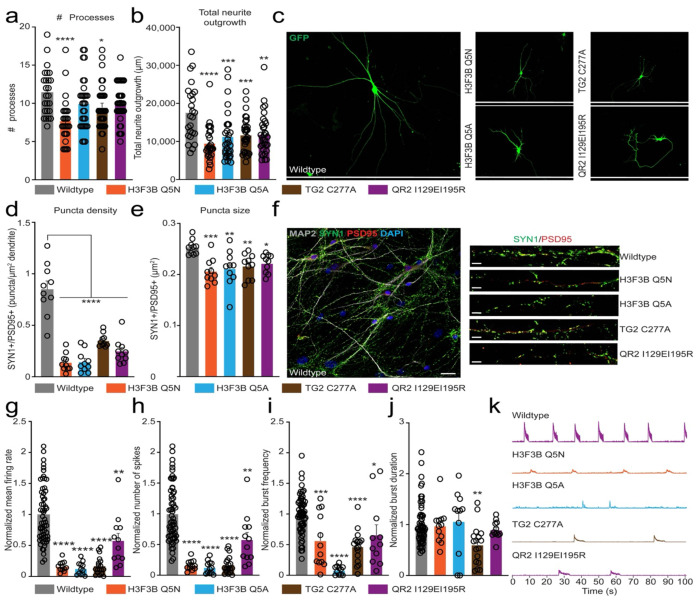
QR2-H3Q5ser interactions are required for human neuronal physiological maturation (**a**) Quantification of the number of neuronal processes in wildtype *vs*. mutant hiPSC neurons [*n* = 25 wildtype cells, 26 H3F3B Q5N cells, 30 H3F3B Q5A cells, 31 TG2 C277A cells, and 32 QR2I129EI195R cells; one-way ANOVA, F_4,139_ = 5.105, *P*=0.0007; post hoc: Dunnett’s MC test – H3F3B Q5N (****P<0.0001) and TG2 C277A (*P=0.0497)]. (**b**) Quantification of total neurite outgrowth in wildtype *vs*. mutant hiPSC neurons [*n* = 25 wildtype cells, 26 H3F3B Q5N cells, 30 H3F3B Q5A cells, 31 TG2 C277A cells, and 32 QR2I129EI195R cells; one-way ANOVA, F_4,139_ = 7.222, *P*<0.0001; post hoc: Dunnett’s MC test – H3F3B Q5N (****P<0.0001), H3F3B Q5A (***P=0.0003), TG2 C277A (***P=0.0008), and QR2 I129EI195R (**P=0.0011). (**c**) Representative images of GFP labeled neurons from wildtype *vs*. mutant hiPSC neurons (scale = 625 μm). (**d**) Quantification of SYN1+/PSD95+ co-puncta density in wildtype *vs*. mutant hiPSC neurons [*n* = 10; one-way ANOVA, F_4,45_ = 43.69, *P*<0.0001; post hoc: Dunnett’s MC test ****P<0.0001. (**e**) Quantification of SYN1+/PSD95+ co-puncta size in wildtype *vs*. mutant hiPSC neurons [*n* = 10; one-way ANOVA, F_4,45_ = 5.723, *P*=0.0008; post hoc: Dunnett’s MC test – H3F3B Q5N (***P=0.0003), H3F3B Q5A (**P=0.0015), TG2 C277A (**P=0.0056), and QR2 I129EI195R (*P=0.0208). (**f**) Representative images of co-puncta labeling from wildtype *vs*. mutant hiPSC neurons (scale = left panel 25 μm; right panel 15 μm ). MEA-based quantification of: (**g**) normalized mean firing rate [*n* = 60 wildtype cells, 12 H3F3B Q5N cells, 12 H3F3B Q5A cells, 18 TG2 C277A cells, and 12 QR2I129EI195R cells; one-way ANOVA, F_4,109_ = 32.19, *P*<0.0001; post hoc: Dunnett’s MC test – **** *P*<0.0001, ** *P*=0.0020]; (**h**) normalized spike number [*n* = 60 wildtype cells, 12 H3F3B Q5N cells, 12 H3F3B Q5A cells, 18 TG2 C277A cells, and 12 QR2I129EI195R cells; one-way ANOVA, F_4,109_ = 32.19, *P*<0.0001; post hoc: Dunnett’s MC test – **** *P*<0.0001, ** *P*=0.0020]; (**i**) normalized burst frequency [*n* = 60 wildtype cells, 12 H3F3B Q5N cells, 12 H3F3B Q5A cells, 18 TG2 C277A cells, and 12 QR2I129EI195R cells; one-way ANOVA, F_4,106_ = 22.28, *P*<0.0001; post hoc: Dunnett’s MC test – **** *P*<0.0001, *** *P*=0.0004, * *P*=0.0121]; and (**j**) normalized burst duration [*n* = 60 wildtype cells, 12 H3F3B Q5N cells, 12 H3F3B Q5A cells, 18 TG2 C277A cells, and 12 QR2I129EI195R cells; one-way ANOVA, F_4,107_ = 3.323, *P*=0.0132; post hoc: Dunnett’s MC test – ** *P*=0.0032]. (**k**) Representative MEA traces from wildtype *vs*. mutant hiPSC neurons. Data shown as mean ± SEM.

Given our observations that disruption of either H3Q5ser itself or its interactions with QR2 produces similar alterations in gene expression programs associated with human neuronal maturation, we next investigated the functional and physiological consequences of these perturbations in mature NGN2-induced neurons. To determine whether QR2-H3Q5ser disruption affects structural maturation, we quantified neuronal morphology at DIV35 using sparse GFP labeling of wildtype and mutant neurons. While the total number of neurites were found to be reduced only in select lines *vs.* wildtype (H3.3Q5N, TG2 C277A; Fig. 4a,c), all four mutants displayed significantly reduced neurite outgrowth (Fig; 4b–c), suggestive of deficits in dendritic maturation. We next examined synaptic architecture to determine whether disruption of QR2-H3Q5ser signaling impairs synaptic connectivity. Synapses were quantified by co-localization of the presynaptic marker SYN1 and the postsynaptic marker PSD95 (Fig. 4d,f). All four mutant lines exhibited a significant reduction in synaptic density, measured as double-positive puncta relative to wildtype neurons. In addition, puncta size was significantly decreased across all mutants (Fig. 4e–f), suggesting that disruption of H3Q5ser or its interaction with QR2 impairs synaptic maturation. Finally, to explore the impact of these perturbations, we performed multielectrode array (MEA) recordings in mature wildtype *vs.* mutant neurons. Consistent with structural and synaptic deficits observed above, all four mutant lines exhibited significant reductions in firing rates (Fig. 4g,k), spiking activity (Fig. 4h,k), and burst frequency (Fig. 4i,k), with comparatively modest effects on burst duration (Fig. 4j–k). Similar impairments were observed following pharmacological inhibition of QR2 in wildtype NGN2-induced neurons (Extended Data Fig. 9m), supporting a specific role for QR2 activity in neuronal network maturation. Together, these findings demonstrate that the H3Q5ser-dependent recruitment of QR2 to active chromatin is required for proper neuronal gene expression, as well as structural and functional maturation of human neurons.

### QR2 binding to H3Q5ser co-recruits POU2F1 to loci important for neuronal maturation

**Figure 5.**
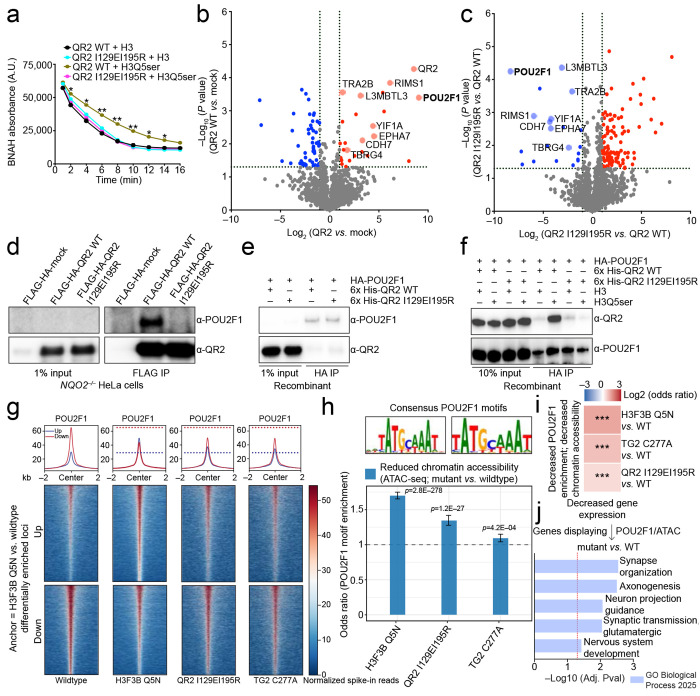
QR2 binding to H3Q5ser co-recruits POU2F1 to loci associated with neuronal maturation (**a**) BNAH enzymatic assay comparing wildtype *vs*. I129EI129R QR2 following spike-in with either H3 unmodified or H3Q5ser peptides. *n* = 3. Two-way RM ANOVA [main effects: time (F_2.088,16.70_ = 22145, *P*<0.0001), treatment (F_3,8_ = 28.33, *P*=0.0001), and treatment x time (F_6.264,16.70_ = 84.04, *P*<0.0001); post hoc: Dunnett’s MC test (*P<0.05, ** *P*<0.01; QR2 wildtype H3Q5ser *vs.* QR2 wildtype H3 unmodified)]. Volcano plots of (**b**) FLAG-HA-tagged QR2 wildtype binders (*vs.* mock) from HeLa nuclear extracts (*NQO2* knockdown background) identified by immunoprecipitation and LC-MS/MS (*n* = 3; −log_10_ *P* value < 0.05; student’s t-test difference > 1), and (**c**) FLAG-HA-tagged QR2 I129EI195R binders (*vs.* FLAG-HA-tagged QR2) from HeLa nuclear extracts (*NQO2* knockdown background) identified by immunoprecipitation and LC-MS/MS (*n* = 3; −log_10_ *P* value < 0.05; student’s t-test difference > 1). (**d**) Immunoblotting validation of QR2-POU2F1 co-immunoprecipitation by FLAG-HA-tagged QR2 wildtype, but not FLAG-HA-tagged QR2 I129EI195R, from HeLa nuclear extracts (*NQO2* knockdown background). (**e**) *in vitro* QR2-POU2F1 co-immunoprecipitation experiment in the absence of H3 peptides. (**f**) *in vitro* QR2-POU2F1 co-immunoprecipitation experiment in the presence of H3Q5ser *vs.* H3 unmodified peptides. (**g**) CUT&RUN-seq heatmaps of POU2F1 enrichment at sites of differential enrichment for POU2F1 in mutant *vs.* wildtype hiPSC neurons (*n* = 3 for all; anchor: H3F3B Q5N *vs.* wildtype). Dashed lines represent wildtype signal. (**h**) Odds ratio analysis (Fisher’s exact test) comparing overlap between POU2F1 consensus motifs displaying loss of enrichment in mutant *vs.* wildtype hiPSC neurons and loss of chromatin accessibility (via ATAC-seq) at these same loci. P values are indicated. (**i**) Odds ratio analysis (Fisher’s exact test) comparing overlap between genes displaying loss of POU2F1 enrichment, reduced chromatin accessibility, and decreased gene expression in mutant *vs.* wildtype hiPSC neurons. *** *P*< 0.001. (**j**) Go Biological Process 2025 enrichment for genes identified in panel **i** (FDR<0.05). Data shown as mean ± SEM. All immunoblotting experiments repeated 3X. See Supplementary Figure 1 for uncropped blots.

Although our data indicate that QR2-H3Q5ser interactions are required for neuronal gene expression and maturation, the chromatin mechanisms underlying this effect remained unclear. We therefore next investigated how QR2 binding to H3Q5ser promotes transcriptional activation. Previous studies had shown that QR2 binds to free 5-HT, thereby inhibiting its endogenous reductase activities^36,37^. Therefore, we assessed whether QR2 binding to H3Q5ser results in similar disruptions to its enzymatic capacity. We performed QR2 enzymatic activity assays *in vitro* (again using BNAH as an electron donor) with purified wildtype QR2 *vs.* QR2 I129EI195R following pre-incubation with non-biotinylated H3_1-18_Q5ser *vs.* H3_1-18_ unmodified peptides. Consistent with its inhibition by free 5-HT, we found that wildtype QR2 displayed significantly attenuated quinone reductase activity in the presence of H3Q5ser *vs.* H3 unmodified peptides; note that such pre-incubation with H3Q5ser peptides had no effect on the QR2 mutant, which cannot bind to the mark (Fig. 5a).

Given the impact of QR2 binding to H3Q5ser on its enzymatic functions, we next hypothesized that QR2 may act to recruit additional co-factors to chromatin when bound to the serotonyl mark, which may, in turn, facilitate the PTM’s impact on permissive gene expression. For this, we revisited our established QR2 knockdown/complementation HeLa cell lines, where we next performed FLAG immunoprecipitations to enrich for QR2 (complemented wildtype QR2 *vs.* QR2 I129EI195R) in chromatin fractions, followed by LC-MS/MS analysis to identify QR2 binding partners whose interactions are dependent on the protein’s ability to engage with H3Q5ser. Following mass spectrometry, we identified numerous proteins that displayed significantly enriched binding to wildtype QR2 in the context of chromatin (Fig. 5b, Supplementary Data 14), with a number of these interactions found to be significantly attenuated in the QR2-H3Q5ser binding-dead mutant line (Fig. 5c, Supplementary Data 14). Among these candidate factors was POU2F1 (aka OCT1; Fig. 5b–d), a broadly expressed transcription factor that has recently been shown to be repurposed in excitatory neurons to regulate chromatin programs important for neurodevelopment^48,49^. To assess whether QR2 binds POU2F1 directly in the absence of a chromatin context in which H3Q5ser is present, we purified both QR2 (wildtype and mutant, 6X-His-tagged) and full length POU2F1 (HA-tagged), and performed co-immunoprecipitation assays *in vitro*. Interestingly, while QR2 and POU2F1 did not appear to interact directly in the absence of H3Q5ser (Fig. 5e), they were found to interact following spike-in of an H3Q5ser peptide (*vs.* unmodified; Fig. 5f), suggestive of a potential allosteric mode of recognition through which QR2 binding to the mark may facilitate structural re-organization of the complex to allow for POU2F1 binding/recruitment to chromatin.

**Extended Data Figure 10.**
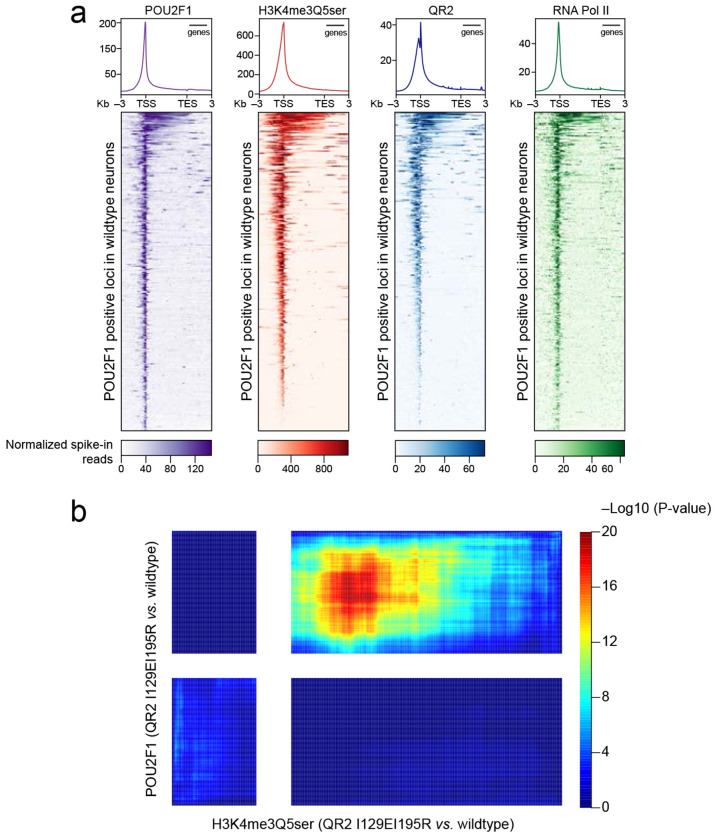
POU2F1 co-enriches with H3K4me3Q5ser and QR2 in hiPSC neurons (**a**) CUT&RUN-seq heatmaps of POU2F1, H3K4me3Q5ser, QR2, and RNA Pol II enrichment (anchored on POU2F1 enriched genes) in wildtype hiPSC neurons at DIV35. *n* = 3 for all. (**b**) RRHO analyses (CUT&RUN-seq) comparing the impact of QR2 I129EI195R mutations on POU2F1 *vs.* H3K4me3Q5ser enrichment.

To examine this possibility in NGN2-induced neurons, we then performed CUT&RUN-seq for POU2F1 in wildtype *vs.* mutant cells. In addition to demonstrating that POU2F1indeed co-enriches with H3K4me3Q5ser and QR2 at TSSs of permissive, RNA Pol II-marked genes (Extended Data Fig. 10a; Supplementary Data 15), these results identified a large number of loci that were enriched for POU2F1 in wildtype neurons but displayed differential enrichment following H3Q5ser depletion or in response to disrupting QR2’s recognition of H3Q5ser (Fig. 5g; Extended Data Fig. 10b; Supplementary Data 15). Importantly, for those loci displaying loss of POU2F1 enrichment in mutant neurons – sites that largely mapped to consensus POU2F1 motifs – we observed that loss of chromatin accessibility at these sites (via ATAC-seq) was strongly correlated (Fig. 5h; Supplementary Data 16), along with a decrease in expression of corresponding genes (Fig. 5i). Finally, for those loci displaying concomitant reductions in POU2F1 enrichment, attenuated chromatin accessibility, and decreased expression in the mutant lines, we found that these genes were highly enriched for gene ontologies related to human neuronal development and maturation, including pathways related to synapse organization, axonogenesis, glutamatergic synaptic transmission, etc. (Fig. 5j; Supplementary Data 17). In sum, our data suggest that during human neuronal differentiation and maturation, QR2 binds to H3Q5ser, leading to the recruitment of important lineage specific transcription factors, such as POU2F1, which, in turn, results in altered chromatin accessibility and activation of genes important for neuronal development.

## DISCUSSION

Here, we explored the possibility that in addition to its previously described roles as a modifier of H3K4me3-mediated *reader* interactions and permissive gene expression, H3Q5ser may recruit additional dedicated binding proteins that facilitate its impact on transcription within mammalian cells. Employing proteomic-, structural-, molecular-, epigenomic-, and cellular-based approaches, we found that the quinone reductase enzyme, QR2, is a *bona fide reader* of H3Q5ser, both *in vitro* and *in cellulo*, and that this interaction can occur in the absence or presence of adjacent H3K4me3. Using X-ray crystallography to determine the structure of QR2 in complex with H3(K4me3)Q5ser, we identified a specific pair of mutations that fully abrogate QR2’s binding to H3Q5ser without impacting QR2’s expression, subcellular localization, or enzymatic functions. Introducing these mutations to human cells, including neurons, resulted in dysregulated gene expression, including those that play critical roles in neuronal maturation. Consistent with the impact of disrupting QR2-H3Q5ser interactions in human neurons, we found that these manipulations promote deficits in human neuronal morphology, synaptic connectivity, and electrophysiology. While QR2 has well documented roles as a quinone reductase in the cytoplasm, its binding to H3Q5ser within the nucleus inhibits its enzymatic activities; however, such binding then allows for the recruitment of additional transcription factors, such as POU2F1, which play key roles in promoting gene expression programs that are required for appropriate patterns of neuronal differentiation and maturation.

While it is now clear from our studies that QR2’s interactions with H3Q5ser in chromatin play important roles in permissive gene transcription, particularly within neurons, it remains unclear to what extent these interactions can explain QR2’s previously documented roles within cells. Physiologically, quinones are reactive molecules that act as oxidizing agents, easily forming reactive oxygen species (ROS). While ROS have essential roles in a variety of cellular functions, including innate immunity^50^, they can also cause significant oxidative damage, which, at high levels, can impair physiological functions through DNA damage and disrupted protein function, resulting in human pathologies such as disorders of aging, cancer, and neurodegenerative disease^51,52^. As mentioned above, QR2 is a flavoprotein closely related to the enzyme QR1^53^, and while a plethora of data is available regarding QR1 and its functions – such as its co-factor, substrate, and inhibitor profile – very little remains known about QR2^35^. Evolutionary research has additionally shown that both QR1 and QR2 evolved from the same common ancestor, with QR1 becoming selectively more efficient in utilizing NADH as a co-factor, and QR2 less efficient in utilizing NADH, resulting in two similar enzymes with different cellular functions^54^. For example, it remains unknown which endogenous quinones, if any, QR2 is capable of enzymatically reducing, and a recent study even demonstrated that QR2 inhibition paradoxically increases ROS^55^. In addition, it was also recently suggested that QR2 may have simply lost its reductase functions throughout evolution in the absence of extremely high levels of ROS^56^. Also, while our data demonstrated that QR2’s enzymatic functions are significantly inhibited when bound to H3Q5ser, such attenuation was not complete, leaving open the possibility that QR2 may still function as a chromatin redox sensing protein when bound to the mark. However, given such evolutionary divergence from QR1, and in light of our findings suggesting a prominent role for QR2-H3Q5ser interactions in mammalian gene expression, this begs the question as to whether previous interpretations of QR2’s functions within cells – which have been solely anchored on the premise that it functions as a quinone reductase – truly encapsulate its primary (or perhaps most important) mechanistic roles in brain or beyond.

In addition, the interplay between QR2 and learning and memory (which require appropriate patterns of neuronal maturation and plasticity) has been previously studied, where an inverse relationship between QR2 expression and memory formation has been observed. In other words, increased expression of QR2 in the hippocampus was found to be associated with impairments in novel memory formation and learning, particularly in Alzheimer’s Disease (AD) models and in aged animals^57–59^. Reducing the expression of QR2 via genetic knockdown or using lentiviruses harboring short hairpin RNAs (shRNAs)^60^ all result in improved novel memory. Importantly, activation of acetylcholine in the cortex or dopamine in the hippocampus repress QR2 expression, resulting in enhanced memory formation during novel experiences. In addition, vital elements that lie upstream of QR2 are often dysregulated with age^61^. Prolonged metabolic stress is another major contributing factor to the sensitivity of novel memory formation in age-related neurodegenerative disease and pathologies^62–64^. While investigating roles for QR2 in memory and learning, QR2 was identified as a removable memory constraint in brain that contributes to memory decline and metabolic stress^58,65^. For example, it has been shown that following acquisition of novel information, activation of miR-182 in the CA1 region of the hippocampus suppresses QR2 expression and reduces the activation of ROS, leading to a decrease in the activity of specific inhibitory interneurons^58,65,66^. In addition, within the cortex, not only does the removal of QR2 enhance memory consolidation, but QR2 inhibition reduces excitability in certain neuronal populations^66^. Importantly, all the aforementioned studies relied on global QR2 knockdown/knockout/overexpression models or QR2 inhibition (using inhibitors, such as yb800, which also abrogate QR2 binding to H3Q5ser), so it is impossible at this time to decipher precisely which resulting phenotypes are the result of QR2’s enzymatic functions *vs.* its roles in mediating H3Q5ser-mediated gene expression (or a combination of both). Although our findings establish a role for QR2-H3Q5ser signaling in neuronal maturation, the present study does not definitively distinguish whether the observed synaptic impairments arise during early neurogenesis or during later stages of synaptogenesis. Future work will be required to dissect how QR2-H3Q5ser signaling contributes to these processes and whether they ultimately relate to QR2’s previously described roles in learning and memory.

Importantly, in humans, QR2 is predominantly expressed in the liver (where QR1 levels are low), as well as in heart, brain, lung, liver, kidney, and skeletal muscle. As such, while our initial studies of QR2-H3Q5ser interactions were largely focused on human neurons given H3Q5ser’s previously established roles in brain, it stands to reason that such interactions may also play equally important roles in other organ systems and/or cell-types, especially given H3 serotonylation’s broad distribution across tissues. Consistent with this possibility, prior constitutive knockout studies in mice have revealed important roles for QR2 in preventing myeloid hyperplasia^67,68^ and other malignancies^69^, yet whether such effects are induced via QR2’s enzymatic *vs.* chromatin functions (or a combination of both) remains unclear. Indeed, future research will be necessary to fully elucidate QR2’s diverse functions in cells; however, we posit that its binding to H3Q5ser likely plays a prominent, and perhaps even evolutionarily conserved, role across organismal systems.

Finally, while our data identified QR2 as a *bona fide reader* of H3Q5ser in mammalian cells, the fact that QR2 was not observed to bind other H3 monoaminylation marks, such as H3Q5dop or H3Q5his, suggests that additional dedicated *readers* may exist for these PTMs, which have yet to be identified. Such exciting possibilities will indeed require further efforts; however, it is our hope that the approaches presented here will serve as a valuable framework for the future identification and functional characterization of additional H3 monoaminylation interacting proteins.

## MATERIALS AND METHODS

### General reagents

#### Antibodies

All primary antibodies used in this study were validated, where appropriate, for use in immunoblotting, immunofluorescence, chromatin immunoprecipitation (ChIP), and CUT&RUN experiments. Histone H3 (Abcam, ab1791, rabbit, 1:5,000), H3Q5ser (Millipore, ABE1791, rabbit, 1:500), H3K4me3Q5ser (Millipore, ABE2580, rabbit, 1:500), NQO2 (Biorbyt, orb400516, rabbit, 1:500), NQO2 (Santa Cruz, mouse, sc-271665, 1:1,000), NQO2 (Abcam, ab181049, rabbit, 1:1,000), RNA polymerase II (Active Motif, 39097, mouse, 1:1,000), NQO1 (Santa Cruz, mouse, sc-32793, 1:1,000), Rabbit IgG (Epicypher, 13-0042), POU2F1/OCT1 (Cell Signaling technology, 8157S, rabbit, 1:500), MAP2 (Abcam, AB5392; chicken, 1:20,000), Synapsin I/II (Synaptic Systems, 106002, rabbit, 1:500), PSD95 (Synaptic Systems, 124308, guinea pig, 1:500), Alexa Fluor 488-conjugated anti-chicken IgY (H+L) (Invitrogen, A-11039, 1: 1,000), Alexa Fluor 555-conjugated anti-rabbit IgG (H+L) (Invitrogen, A-21428, 1:1,000), Alexa Fluor 647-conjugated anti-guinea pig IgG (H+L) (Invitrogen, A-21450, 1:1000).

#### Cell culture materials

DMEM (Gibco, 10569044); fetal bovine serum (FBS; Gibco, A5670801); penicillin–streptomycin (Gibco, 15140122); Basal Medium Eagle (Gibco, 21010046); HyClone bovine growth serum (Cytiva, SH30541.03); GlutaMAX^™^ supplement (Gibco, 35050061); potassium chloride (KCl; Invitrogen, AM9640G); tetrodotoxin citrate (TTX; Tocris, 1069); DL-2-amino-5-phosphonopentanoic acid (DL-AP5; Tocris, 0105); Geltrex^™^ matrix (Gibco, A1413302); DMEM/F-12 (Gibco, 11320033); StemFlex^™^ medium (Gibco, A3349401); EDTA (Invitrogen, AM9260G); Dulbecco’s phosphate-buffered saline (DPBS; Gibco, 14190144); Chroman 1 (Sigma, SML3272); doxycycline hyclate (Sigma, D3447); Opti-MEM^™^ I reduced-serum medium (Gibco, 31985070); Lipofectamine^™^ Stem transfection reagent (Invitrogen, STEM00015); Lipofectamine^™^ 3000 transfection reagent (Invitrogen, L3000008); puromycin dihydrochloride (Sigma, P9620); Accutase (STEMCELL Technologies, 07920); Neurobasal^™^ medium (Gibco, 21103049); Neurobasal^™^-A medium (Gibco, 10888022); B-27^™^ supplement (Gibco, 17504044); cytosine β-D-arabinofuranoside (Ara-C; Sigma, 251010); calcium chloride (CaCl_2_; Sigma, 21115); magnesium chloride (MgCl_2_; Sigma, 63069); HEPES (Gibco, 15630080); N2 supplement (Gibco, 17502048); insulin (Sigma, I0516); Hank’s balanced salt solution (HBSS; Gibco, 14175103); papain (Worthington Biochemical Corporation, NC9597281); HyClone^™^ Cosmic Calf Serum (Cytiva, SH30087.04); sodium pyruvate (Gibco, 11360070); MEM non-essential amino acids solution (NEAA; Gibco, 11140050); 2-mercaptoethanol (Sigma, M3148); Bambanker cryopreservation medium (FUJIFILM Wako, 302-14681); 0.25% trypsin–EDTA (Gibco, 25200056); polyethylenimine (PEI, 25 kDa; Polysciences, 23966); poly-L-ornithine hydrobromide (Sigma, P4957); polybrene (Sigma, TR-1003); mTeSR^™^ Plus medium (STEMCELL Technologies, 100-0276); CloneR^™^ supplement (STEMCELL Technologies, 05888).

### HEK 293T and HeLa cell culture

HEK 293T and HeLa cell lines were obtained from the American Type Culture Collection (ATCC) and grown in DMEM supplemented with 10% FBS with 100 μg ml^−1^ streptomycin and 100U ml^−1^ penicillin. Cells were maintained at 37 °C in a 5% CO_2_, 95% humidified incubator.

### HeLa cell H3 peptide pulldowns and mass spectrometry

#### Preparation of HeLa nuclear extracts

HeLa cells were cultured in DMEM to confluence, harvested, and washed with ice-cold DPBS. Nuclei were isolated by hypotonic lysis using a Dounce homogenizer (20 strokes) in hypotonic buffer containing: 10 mM HEPES (pH 7.5), 1.5 mM MgCl_2_, 10 mM KCl, 0.5 mM DTT, 1 mM PMSF, and a protease inhibitor cocktail. Nuclei were collected by centrifugation and lysed in high-salt buffer containing: 50 mM HEPES (pH 7.5), 10% (v/v) glycerol, 500 mM KCl, 5 mM MgCl_2_, 0.1% NP-40, 0.5 mM DTT, 1 mM PMSF, and protease inhibitor cocktail for 30 min at 4 °C with rotation. Lysates were clarified by centrifugation at 13,000 rpm for 10 min at 4 °C, and the resulting supernatant was dialyzed overnight at 4 °C against 2 L of dialysis buffer containing: 50 mM Tris-HCl (pH 8.0), 5% (v/v) glycerol, 150 mM KCl, 2 mM MgCl_2_, 0.1% NP-40, and 1 mM DTT. Following dialysis, extracts were transferred to fresh tubes and further clarified by centrifugation at 13,000 rpm for 20 min at 4 °C. The final supernatant was used for peptide pulldown assays.

#### H3 peptide pulldown assay

For each pulldown assay, 100 μg of biotinylated histone peptide (H3_1-18_Q5ser *vs.* H3_1-18_ unmodified) was incubated overnight at 4 °C with rotation with 100 μL of prewashed immobilized streptavidin beads (Dynabeads^™^ M-280 Streptavidin, Invitrogen, 11206D) in DPBS containing 0.01% Triton X-100. For each immunoprecipitation, 40 μL of a 50% peptide-bead slurry was incubated with dialyzed HeLa nuclear extract for 4 h at 4 °C with rotation. Peptide-protein complexes were collected by centrifugation at 1,000 rpm for 1 min, and beads were washed four times with dialysis buffer followed by four washes with detergent-free dialysis buffer (50 mM Tris-HCl (pH 8.0), 150 mM KCl, 2 mM MgCl_2_, and 1 mM DTT). For western blot analysis, beads were boiled for 8 min in 30 μL of denaturing sample buffer and proteins were resolved by SDS-PAGE.

#### Sample preparation for mass spectrometry

For *liquid chromatography tandem mass spectrometry analysis (LCMS)* , proteins bound to histone peptides were reduced (10mM Dithiothreitol) and alkylated (30mM iodoacetamide) followed by partial trypsinization (10ng/uL, Promega). elution . After 4h at room temperature, the supernatant was transferred to new vial and re-trypsinized overnight. Digestion was halted by addition of 10% trifluoroacetic acid (TFA; Thermo Scientific, 85183 and peptides were solid phase extracted prior to being analyzed by direct loading reversed-phase nano–LC–MS/MS using a Fusion Lumos mass spectrometer operated in high/high acquisition mode. Petptides were separated using a packed-in-emitter 12cm/75um ID/3um C18 column (Nikkyo Technos Co., Ltd. Japan). Peptides were eluted using a gradient delivered at 300nL/min increasing from 2% Buffer B (0.1% formic acid in 80% acetonitrile) / 98% Buffer A (0.1% formic acid) to 35% Buffer B / 65% Buffer A, over 70 minutes (EasyLC 1200, Thermo Scientific). All solvents were LCMS grade (Optima, Fisher Scientific).

#### Mass spectrometry data processing and analysis

Mass spectrometry data were searched against the UniProt human protein database using MaxQuant (v. 2.0.3.0 or v. 2.4.2.0 )^70^ using full tryptic constraints. Oxidation of methionine residues and protein N-terminal acetylation were specified as variable modifications, and carbamidomethylation of cysteine residues was set as a fixed modification. False Discover Rate (FDR) were set at 1% for both peptides and proteins. The experiment comprised two peptide conditions (H3_1-18_Q5ser *vs.* H3_1-18_ unmodified), each analyzed with four biological replicates. Quantitative data were processed using Perseus v. 2.0.10.0^70^. Reverse database hits and potential contaminants were removed prior to analysis. Log_2_-transformed intensity-based absolute quantification (iBAQ) values were median-normalized on a per-sample basis. Proteins were required to be quantified in at least three replicates in at least one condition to be included in downstream analyses. Missing values were imputed using a normal distribution (width = 0.3; downshift = 1.8). Statistical comparisons between unmodified H3 and H3Q5ser conditions were performed using a permutation-based two-sided t-test with an FDR threshold of 0.05.

### Western blotting

Proteins were electrophoresed on 4–12% NuPAGE Bis-Tris protein gels (Invitrogen) and transferred to nitrocellulose membranes. Efficient transfer was always confirmed with direct blue staining (0.1% stock aqueous solution in milliQ water) before being incubated with primary antibodies overnight at 4 °C (all membranes were blocked in 5% milk or 5% BSA depending on the primary antibody used). Membranes were then washed and incubated with peroxidase-labelled secondary antibodies (1:2,000–1:10,000, depending on the primary antibody used). Bands were detected using SuperSignal^™^ West Femto Maximum Sensitivity Substrate (Thermo Scientific, 34095) and imaged on a ChemiDoc Imaging System (Bio-Rad) under chemiluminescent detection. Molecular weight markers were visualized using the colorimetric (Ponceau S) imaging setting of the ChemiDoc system. Chemiluminescent images were acquired using fixed exposure settings and are presented in the main figures. Composite images merging chemiluminescent signals and molecular weight marker images were generated for the supplementary materials to display molecular weight references.

### Chemical proteomics profiling and validations

#### Culturing of SK-N-SH cell

SK-N-SH cells (American Type Culture Collection, HTB-11) were cultured in DMEM supplemented with 10% fetal bovine serum (FBS), 100 U/mL penicillin, and 100 μg/mL streptomycin. Cells were maintained in a humidified 37 °C incubator with 5% CO_2_.

#### Protein expression and purification

Plasmids for human QR1 and QR2 proteins, containing an N-terminal hexahistidine (6×His) tag in a pProEX-HTa expression vector, were generous gift from R. M. Vabulas. Point mutations in QR2 were introduced using the Mut Express Universal Fast Mutagenesis Kit (Vazyme) and verified by Sanger sequencing. Full-length QR1/QR2 proteins were expressed in *Escherichia coli* Rosetta (DE3) cells. Protein expression was induced with 0.2 mM isopropyl β-D-1-thiogalactopyranoside (IPTG) at 16 °C overnight in LB medium. Cells were lysed by sonication or French press homogenization in lysis buffer (1 × PBS, pH 7.4, 20 mM imidazole, 1 mM PMSF, 0.5× Roche Complete EDTA-free protease inhibitor cocktail). Clarified lysates were obtained by centrifugation at 25,000 × *g* for 30 min at 4 °C. The supernatant was incubated with Ni–NTA resin for 2 h at 4 °C. Following target protein loading, the resin was washed extensively with 1 × PBS and wash buffer (1× PBS, pH 7.4; 20 mM imidazole). For removal of bound flavin cofactors, the resin was washed with denaturing buffer (1 × PBS, pH 7.4; 2 M urea; 2 M KBr) and incubated in this buffer. This process was repeated until fluorescence spectroscopy (Ex 450 nm/Em 530 nm) detected no FAD signal in the supernatant. The resin was then incubated overnight in 1× PBS (pH 7.4) containing 20 mM FAD to reload the cofactor. Proteins were eluted with elution buffer (1 × PBS, pH 7.4, 500 mM imidazole) and further purified by size-exclusion chromatography (SEC) using a Superdex 75 column (Cytiva) equilibrated with gel filtration buffer (50 mM HEPES, pH 7.4, 150 mM NaCl). An identical purification protocol was applied to all QR2 mutant proteins.

#### Preparation of SK-N-SH lysates

Harvested SK-N-SH cells were resuspended in hypotonic buffer (10 mM HEPES, pH 7.5, 2 mM MgCl_2_, 0.1% Tween-20, 20% glycerol, 2 mM PMSF, and Roche Complete EDTA-free protease inhibitors) and lysed on ice using a glass Dounce homogenizer (pestle B, 30 strokes). The homogenate was centrifuged at 16,000 × *g* for 15 min at 4 °C. The supernatant was collected and the pellet was resuspended in high-salt buffer (50 mM HEPES, pH 7.5, 420 mM NaCl, 2 mM MgCl_2_, 0.1% Tween-20, 20% glycerol, 2 mM PMSF, and Roche Complete EDTA-free protease inhibitors) and homogenized with a Dounce homogenizer (pestle B, 3 × 30 strokes). The suspension was centrifuged at 16,000 × *g* for 15 min at 4 °C. Supernatants from two portions were pooled and clarified again at 21,000 × *g* for 15 min at 4 °C. Protein concentration was determined using the Bradford assay (Bio-Rad).

#### Photo-crosslinking experiments

Photoaffinity probes in the absence or presence of different concentrations of competitors were incubated with recombinant proteins (1 μM) or cell lysate (1.0-1.5 mg/mL) in binding buffer (150 mM NaCl, 50 mM HEPES, pH 7.5, 0.05% Tween 20, 20% glycerol) for 10 min at 4 °C. Then, the samples were irradiated at 365 nm (Spectrolinker XL-1000) for 20 min in 96-well (recombinant proteins, 75 μL each well) or 6-well plate (cell lysate, 1-2 mL each well) on ice.

#### Copper(I)-catalyzed azide–alkyne cycloaddition (CuAAC)

To the prepared photo-cross-linking samples, 100 μM rhodamine-N_3_ for in-gel fluorescence (10 mM stock in DMSO) or diazo-biotin-N_3_ for pulldown (5 mM stock in DMSO) was added, followed by 1 mM TCEP (freshly prepared 50 mM stock in H_2_O), 100 μM TBTA (10 mM stock in DMSO), and finally the reactions were initiated by the addition of 1 mM CuSO_4_ (freshly prepared 50 mM stock in H_2_O). The reactions were incubated for 1 h at room temperature with regular vortexing. The reactions were quenched by adding 5 volumes of ice-cold acetone and placed at −20 °C overnight to precipitate proteins.

#### In-gel fluorescence scanning

For in-gel fluorescence, acetone-precipitated protein pellets were collected by centrifugation at 6,000 × *g* for 5 min at 4 °C, washed twice with ice-cold methanol, and air-dried for 10 min. Pellets were resuspended in 1 × LDS sample buffer (Invitrogen) containing 50 mM DTT and heated at 95 °C for 10 min before separation by SDS-PAGE. Fluorescently labeled proteins were visualized using an Amersham ImageQuant 800 (excitation 535 nm, emission 580 nm).

#### Enrichment of biotinylated proteins

For enrichment of biotinylated proteins, acetone-precipitated pellets were centrifuged at 3,500 × *g* for 5 min at 4 °C, washed twice with ice-cold methanol, and air-dried for 10 min. Protein pellets were dissolved in PBS containing 4% SDS, 20 mM EDTA, and 10% glycerol by vortexing and heating, then diluted with 1 × PBS to a final SDS concentration of 0.5%. High-capacity streptavidin agarose beads (Thermo Fisher Scientific) were added, and samples were rotated for 1.5 h at room temperature to capture biotinylated proteins. The beads were stepwise washed by 1 × PBS with 0.2% SDS (5 times), 6 M urea in 1 × PBS with 0.1% SDS (5 times) and 250 mM NH_4_HCO_3_ with 0.05% SDS (5 times). The enriched proteins were eluted by incubating the beads with 25 mM Na_2_S_2_O_4_, 250 mM NH_4_HCO_3_, and 0.05% SDS for 1 h. The eluted proteins were dried with SpeedVac.

For mass spectrometry samples, before washing by 250 mM NH_4_HCO_3_ with 0.05% SDS, beads were incubated in 10 mM DTT, 6 M urea in 1 × PBS with 0.1% SDS at 37 °C for 1 h and then were added 30 mM iodoacetamide at room temperature for another 30 min in dark to reduce and alkylate cysteines.

#### TMT-based quantitative proteomics

SpeedVac samples were redissolved in 100 μL water. Proteins were precipitated by sequential addition of 400 μL methanol, 100 μL chloroform, and 300 μL water, followed by centrifugation at 16,000 × *g* for 2 min; the upper aqueous phase was removed, and the protein interphase was washed with 600 μL pre-chilled (−20 °C) methanol and centrifuged again under the same conditions. The resulting protein pellets were air-dried and reconstituted in 50 mM triethylammonium bicarbonate (TEAB, pH 8.0), then digested with 0.2 μg MS-grade trypsin at 37 °C for 12 h with shaking, followed by an addition of 0.1 μg trypsin for 2 h to ensure complete digestion. Peptide concentrations were measured using the Pierce Quantitative Fluorometric Peptide Assay (Thermo Fisher Scientific), and peptides in 80 μL (~4 mg) of 50 mM TEAB were labeled with TMT reagents (Thermo Fisher Scientific) dissolved in 20 μL acetonitrile for 1 h at room temperature. Labeling reactions were quenched with 4 μL of 5% hydroxylamine for 15 min at room temperature, after which labeled peptides were combined and dried in SpeedVac, followed by desalting using Sep-Pak Vac tC18 cartridges (Waters), and dried again. Peptides were fractionated on an XBridge Peptide BEH C18 column (300 Å, 3.5 μm, 2.1 mm × 250 mm) using a 2–80% acetonitrile gradient in 10 mM ammonium bicarbonate (pH 10.0) over 50 min, dried, and reconstituted in 0.1% formic acid for LC-MS/MS.

#### Binding-site mapping sample preparation for QR2

For binding-site mapping, recombinant QR2 (1 μM) was incubated with probes 2 and 3 (5 μM each) in 3 mL of binding buffer (50 mM HEPES, 150 mM NaCl, 2 mM MgCl_2_, 0.1% Tween-20, 20% glycerol, pH 7.5) for 10 min at 4 °C, followed by UV irradiation at 365 nm for 20 min. After crosslinking, acid-cleavable DADPS biotin-azide (100 μM) was added together with TCEP (1 mM) and TBTA (100 μM), and CuAAC was initiated by adding 1 mM CuSO_4_ and incubating for 1.5 h at room temperature before quenching with four volumes of ice-cold acetone and overnight precipitation at −20 °C. Pellets were collected by centrifugation (3,500 × *g*, 5 min, 4 °C), washed twice with ice-cold methanol, air-dried, and dissolved in 1 × PBS containing 4% SDS, 20 mM EDTA, and 10% glycerol, then diluted to 0.5% SDS and incubated with high-capacity streptavidin agarose beads (Thermo Fisher Scientific) for 1.5 h at room temperature. Beads were washed sequentially with PBS + 0.2% SDS, PBS + 0.1% SDS + 6 M urea, and resuspended in 6 M urea in 100 mM NH_4_HCO_3_. Then, the samples were reduced with DTT (10 mM, 1 h, room temperature), alkylated with iodoacetamide (30 mM, 30 min, dark, room temperature), followed by washed and resuspended in 1 M urea in 100 mM NH_4_HCO_3_ for on-bead digestion with trypsin (1 μg, 16 h, 37 °C). SC-OH-labeled peptides were eluted twice with 100 μL 2% formic acid (30 min each), followed by two washes with 50% acetonitrile + 1% formic acid. All eluates and washes were pooled, dried in a SpeedVac, resuspended in 200 μL 0.5% acetic acid, and desalted using StageTips for LC-MS/MS.

#### LC–MS/MS analysis and data analysis

The TMT samples were analyzed on an Orbitrap Ascend Tribrid mass spectrometer coupled to a Vanquish^™^ Neo UHPLC system (Thermo Fisher Scientific). Peptides were first loaded onto a PepMap 100 C18 trapping cartridge (5 μm, 300 μm × 5 mm) and subsequently separated on a PepMap RSLC C18 analytical column (2 μm, 75 μm × 50 cm). Data acquisition was performed using Thermo Xcalibur 4.3 software. Electrospray ionization was achieved by applying a voltage of 1,800 V through the PEEK junction at the inlet of the microcapillary column. Peptides were separated with a 4–40% acetonitrile gradient in 0.1% formic acid over 140 min at a flow rate of 250 nL/min. Data were acquired in data-dependent acquisition (DDA) mode optimized for TMT-based quantitation. Full MS1 scans were collected in the Orbitrap at a resolution of 120,000 over an m/z range of 400–1,600 Th, with an automatic gain control (AGC) target of 4E5 and a maximum injection time (IT) of 50 ms. The most intense precursor ions above the defined intensity threshold were sequentially selected for MS2 fragmentation using collision-induced dissociation (CID) with a collision energy of 30%, activation time of 10 ms, and an isolation width of 0.7 m/z. MS2 spectra were acquired in the ion trap at turbo scan rate, with an AGC target of 1E4 and IT of 35 ms. For TMT reporter ion quantitation, synchronous precursor selection (SPS) MS3 scans were performed on up to 20 MS2 fragment ions using higher-energy collisional dissociation (HCD) with a normalized collision energy (NCE) of 55%. MS3 spectra were acquired in the Orbitrap at a resolution of 30,000, with an AGC target of 1E5 and IT of 200 ms. Dynamic exclusion was set to 60 s to prevent repeated analysis of the same precursor ions, and charge states of 2–6 were considered for fragmentation. Proteome Discoverer (v3.0) was used to search the raw data against the UniProt *Homo sapiens* (UP000005640). Fixed modifications included cysteine carbamidomethylation and TMT-tag labeling on lysine side chains and peptide N-termini. Variable modifications included methionine oxidation, protein N-terminal acetylation, and methionine loss. Mass error tolerances were set to 20 ppm for MS1 spectra, 0.1 Da for MS2 spectra, and 20 ppm for MS3 spectra. A maximum of two missed cleavages was allowed. The false discovery rate (FDR) for both peptides and proteins was set at 0.01. Missing values were imputed using the low-abundance sampling method. Quantification was based on five biological replicates.

Binding-site mapping samples were analyzed on a Q Exactive PLUS mass spectrometer (Thermo Fisher Scientific) coupled to an Ultimate 3000 RSLCnano system. Peptides were first loaded onto a PepMap 100 C18 trapping cartridge (5 μm, 300 μm × 5 mm) and then separated on a PepMap RSLC C18 analytical column (2 μm, 75 μm × 25 cm). Data acquisition was performed using Thermo Xcalibur v4.3 software. Peptides were eluted with a 4–40% acetonitrile gradient in 0.1% formic acid over 90 min at a flow rate of 300 nL/min. Electrospray ionization was achieved by applying 1,800 V through a PEEK junction at the column inlet.

MS acquisition was conducted in data-dependent acquisition mode. Survey scans (m/z 300–1,800) were acquired in the Orbitrap at a resolution of 70,000, followed by HCD fragmentation of the top 20 most intense precursors (isolation window ±1.5 m/z, resolution 17,500). Automatic gain control (AGC) targets were set to 1 × 10^6^ ions for MS1 and 5 × 10^4^ ions for MS2, with maximum ion injection times of 30 ms and 50 ms, respectively. The normalized collision energy for HCD was 27%. Singly charged ions, ions with >8 charges, and unassigned charge states were excluded from MS2 selection.

Raw data were processed using Proteome Discoverer v3.0, with SC-OH (exact mass: 326.1777 Da) specified as a variable modification on any residue. The NQO2 single-protein FASTA file was used for database searching. Default parameters were applied for identification and quantification: parent MS tolerance of 20 ppm, MS/MS tolerance of 20 ppm, minimum peptide length of 6 amino acids, and up to 2 missed cleavages. Peptide quantification was based on peptide-spectrum match (PSM) counts.

### Vector construction and recombinant QR2 protein purification

Human *NQO2* cDNA was initially cloned into the pUC19 vector. Site-directed mutagenesis was performed to generate the I129E/I195R *NQO2* mutant. All constructs were verified by Sanger sequencing. Wildtype and mutant *NQO2* inserts were subsequently subcloned into the pET28a expression vector (Addgene #69864) to generate pET28a–*NQO2* and pET28a–*NQO2*(I129E/I195R), respectively. Recombinant wildtype and mutant QR2 proteins were expressed in *Escherichia coli* BL21 cells. Protein expression was induced with 0.5 mM isopropyl β-D-l-thiogalactopyranoside (IPTG) at 16 °C overnight in LB medium. Cells were harvested and resuspended in lysis buffer containing: 500 mM NaCl, 20 mM Tris-HCl (pH 7.5), and 10% (v/v) glycerol. Cells were lysed and clarified by centrifugation, and the supernatant was applied to a HisTrap affinity column (GE Healthcare). The column was washed with five column volumes of lysis buffer, and bound proteins were eluted with buffer containing: 150 mM NaCl, 20 mM Tris-HCl (pH 7.5), and 500 mM imidazole. Purified proteins were buffer-exchanged into storage buffer (150 mM NaCl, 20 mM Tris-HCl, pH 7.5), concentrated to approximately 10 mg mL^−1^, aliquoted, and stored at −80 °C.

### Protein preparation for X-ray crystallography and ITC

Human *NQO2* was subcloned into the pTrcHis2C expression vector (Thermo Fisher Scientific) to generate a construct encoding a C-terminal hexahistidine (6×His) tag. Point mutations in *NQO2* were introduced using QuikChange site-directed mutagenesis and verified by Sanger sequencing. Full-length QR2 proteins were expressed in *Escherichia coli* BL21 (DE3) cells. Protein expression was induced with 0.2 mM isopropyl β-D-l-thiogalactopyranoside (IPTG) at 16 °C overnight in LB medium. Cells were harvested by centrifugation, resuspended in buffer A (150 mM NaCl, 20 mM Tris-HCl, pH 7.5, 10 mM imidazole), and lysed using an EmulsiFlex-C3 high-pressure homogenizer (Avestin). Clarified lysates were incubated with Ni–NTA affinity resin for purification.

To remove bound flavin cofactors, the resin-bound protein was washed with buffer B (buffer A supplemented with 2 M KBr and 2 M urea), followed by overnight incubation in buffer A containing 10 mM flavin adenine dinucleotide (FAD) to reload the cofactor. Eluted protein was further purified by anion-exchange chromatography using a Q HP column (Cytiva) and size-exclusion chromatography on a Superdex 75 column (Cytiva). All QR2 mutant proteins were purified using the same procedure. H3 peptides were prepared as previously described^7^ or synthesized commercially (Beijing SciLight Biotechnology).

### Isothermal titration calorimetry

Isothermal titration calorimetry (ITC) experiments were performed at 25 °C using a MicroCal PEAQ-ITC instrument (Malvern Instruments). The sample cell was loaded with 200 μL of QR2 protein (100 μM), and titrated with 17 sequential injections of histone peptide or serotonin (5-HT; MedChemExpress) at a concentration of 1 mM. Titration data were analyzed using Origin 7.0 software and fitted to a single-site binding model. ITC statistics are provided in Extended Data Table 1.

### Crystallization, data collection, and structure determination

Crystallization trials were performed using the sitting-drop vapor diffusion method at 18 °C. Purified QR2 protein (10 mg mL^−4^) was preincubated with H3Q5ser or H3K4me3Q5ser peptides at a 1:10 molar ratio in buffer containing: 100 mM NaCl and 20 mM Tris-HCl (pH 7.5). Crystals of the QR2–H3Q5ser complex were obtained under multiple reservoir conditions, including mixtures containing polyols, imidazole/MES buffers, hexylene glycol, and polyethylene glycol (PEG) 1000 and PEG 3350 at pH 6.5. Crystals of the QR2-H3K4me3Q5ser complex were obtained under similar conditions, using alternative salt components.

Crystals were cryoprotected and flash-frozen in liquid nitrogen prior to data collection. X-ray diffraction data were collected at the Shanghai Synchrotron Radiation Facility. Diffraction images were indexed, integrated, and scaled using HKL2000. Structures were solved by molecular replacement using MOLREP^71^ from the CCP4 suite, with the QR2 structure (PDB ID: 1QR2) as the search model. Model building and refinement were carried out using COOT^72^ and PHENIX^73^, respectively. Data collection and refinement statistics are provided in Extended Data Table 2.

### Generation of unmodified H3 and H3Q5ser nucleosomes

#### Peptide synthesis

Synthetic peptide α-thioesters used for semisynthesis were manually synthesized on Trityl-OH resin (ChemMatrix). The resin was chlorinated in dry DCM with 12 equivalents of thionyl chloride and 24 equivalents of pyridine (2 x 2 h). Excess thionyl chloride was quenched and washed with a 10% DIPEA/DCM solution. Following the chlorination, hydrazine loading was performed in DMF using 10 equivalents of Fmoc-carbazate, and 10 equivalents of DIPEA (2 x 2 h). Unreacted trityl-chloride resin was then capped with 50 equivalents of methanol in DMF (30 min) and washed extensively. Subsequent peptide synthesis was carried out through iterative cycles of Fmoc deprotection (20% piperidine in DMF with 0.1 M HOBt; 1 x 1 min and 1 x 20 min) and amino acid coupling (5 equivalents of standard Fmoc-protected amino acid, 4.9 equivalents of HATU, and 10 equivalents of DIPEA for 2 × 30 min), with extensive DMF washes between steps. Double couplings were performed when necessary to ensure complete acylation. Serotonylated glutamine residues were introduced by incorporation of Fmoc-L-glutamic acid γ-allyl ester (Chem-Impex; Coupling condition: 2 equivalents of Fmoc-L-glutamic acid γ-allyl ester, 1.9 equivalents of HATU, and 5 equivalents of DIPEA for 2 x 2 h), followed by allyl deprotection and serotonin coupling as previously described^1^. Completed peptides were cleaved and deprotected using 92.5% trifluoroacetic acid (TFA), 2.5% ethanedithiol, 2.5% triisopropylsilane, and 2.5% water, precipitated with diethyl ether, and air-dried. Peptide hydrazides were converted to C-terminal thioesters via the peptide acyl pyrazole intermediates following established protocols^74^. Briefly, 1 mM crude peptide hydrazides were dissolved in 100 mM phosphate buffer (pH = 3) with 6 M guanidinium hydrochloride and 2.5 mM acetylacetone. Excess 4-mercaptophenylacetic acid was then added to the solution, and the suspensions were vigorously shaken overnight at room temperature. The reaction mixtures were centrifuged, and the supernatant containing the products were filtered. The peptide MPAA thioesters were purified on preparative reversed-phase HPLC using a 0–30% solvent B (10% water, 90% acetonitrile with 0.1% TFA) gradient over 60 min.

#### Preparation of recombinant histones

Recombinant human histones H2A, H2B, and H4 were expressed in *E. coli* BL21(DE3) cells and purified with minor modifications to established protocols. Cultures were grown in LB medium at 37 °C to an OD_600_ of ~0.6 and induced with 1 mM IPTG for 3–4h. Cells were harvested, resuspended in lysis buffer (20 mM Tris-HCl pH 7.5, 200 mM NaCl, 1 mM EDTA, 1 mM β-mercaptoethanol, 0.1% Triton X-100), and lysed by sonication.

Inclusion bodies were collected by centrifugation, washed twice in lysis buffer lacking detergent, and extracted in resuspension buffer (7 M urea, 10 mM Tris-HCl, 1 mM EDTA, 100 mM NaCl, 1 mM DTT). Clarified extracts were purified by cation-exchange chromatography using a HiTrap SP FF column with a linear NaCl gradient. Histone-containing fractions were pooled and further purified by preparative reversed-phase HPLC.

#### Preparation of truncated histone H3

N-terminally His_6_–SUMO–tagged human histone H3(14–135, K14C) was expressed in *E. coli* BL21(DE3). Cells were lysed under denaturing conditions (6 M guanidinium hydrochloride), and the tagged protein was purified by Ni–NTA affinity chromatography. Eluted protein was stepwise dialyzed to remove denaturant and refold the protein. Ulp1 protease was added to cleave the His_6_–SUMO tag. Following proteolysis, solubilized truncated histone was purified by preparative reversed-phase HPLC.

#### Semisynthesis of modified histone H3

Full-length histone H3 was generated by native chemical ligation between peptide α-thioesters corresponding to residues 1–13 and truncated H3(14–135, K14C). Ligation reactions were performed in a well-ventilated chemical hood in 200 mM sodium phosphate buffer (pH = 7.0–7.2) containing 6 M guanidinium hydrochloride, 50 mM TCEP, and 2% v/v TFET at room temperature overnight. Completion of ligation was confirmed by HPLC and ESI–MS. The ligation product was purified by preparative reversed-phase HPLC and treated with 2-bromoethylammonium bromide to convert the non-native cysteine at position 14 into a lysine analogue following established protocols^75^. Final products were purified by semi-preparative HPLC and validated by HPLC and ESI–MS.

#### Histone octamer refolding

Histone octamers were assembled using established protocols.^1^ Individual histones were dissolved in unfolding buffer (6 M guanidinium chloride, 20 mM Tris-HCl pH 7.9, 1 mM DTT), quantified by absorbance at 280 nm, and combined at a molar ratio of H3:H4:H2A:H2B = 1:1:1.1:1.1 to a final concentration of 1 mg mL^−1^. The mixture was dialyzed stepwise into refolding buffer (2 M NaCl, 10 mM Tris-HCl pH7.9, 1 mM EDTA, 1 mM DTT) at 4 °C. Refolded octamers were purified by size-exclusion chromatography using a Superdex S200 Increase column. Pure fractions were pooled, adjusted to 50% glycerol, and stored at −20 °C.

#### DNA preparation

Biotinylated Widom 601 DNA containing 15-bp overhangs was prepared for mononucleosome assembly. The target fragment was amplified by PCR with Pfu DNA polymerase. A 5′-biotinylated forward primer (5’-Biotin-TACTACGCGGCCGCCCTGG-3’) and an unmodified reverse primer (5’-CTGGATCTTACATGCACAGGATGTATATATCTGACACGTGC-3’) were synthesized and HPLC-purified commercially (IDT). The PCR product was purified by ethanol precipitation. Briefly, sodium acetate (0.1 volumes of 3 M solution, pH 5.3) and ethanol (2.5 volumes) were added to the reaction mixture, and the DNA product was precipitated overnight at −20 °C. Precipitated DNA was collected by centrifugation and resuspended in a minimal volume of nuclease-free water. The sample was further purified using PCR purification silica columns according to the manufacturer’s instructions and eluted in nuclease-free water. DNA concentration was determined by UV absorbance using a NanoDrop spectrophotometer.

#### Nucleosome reconstitution

Nucleosomes were assembled by salt-gradient dialysis. Histone octamers were mixed with 601 DNA in octamer refolding buffer (10 mM Tris-HCl, 2 M NaCl, 0.5 mM EDTA, 1 mM DTT, pH 7.8) and dialyzed stepwise at 4 °C to decreasing the salt concentrations (from 2 M NaCl to 1.4 M KCl, and then to 10 mM KCl overnight via a peristaltic pump) using a Slide-A-Lyzer MINI dialysis device (3.5-kDa cutoff, Thermo Scientific). Following overnight dialysis, the samples were incubated at 37°C for 30 minutes to promote nucleosome repositioning and subjected to two final rounds of dialysis in nucleosome assembly end buffer. Samples were clarified by centrifugation, and nucleosome concentrations were determined by absorbance at 260 nm. Nucleosome quality was assessed by native polyacrylamide gel electrophoresis and ethidium bromide staining.

### *in vitro* pulldown assays

*in vitro* pulldown assays were performed using either biotinylated mononucleosomes or biotinylated histone peptides as binding substrates. For each assay, 1 μg of biotinylated substrate was preincubated with 10 μL of streptavidin Dynabeads^™^ M-280 at 4 °C to allow immobilization. Substrate–bead complexes were then incubated with 1 μg of purified recombinant proteins in 200 μL of binding buffer containing: 150 mM NaCl, 20 mM Tris-HCl (pH 7.5), and 0.1% NP-40 for 1 h at 4 °C with rotation. Following incubation, beads were washed three times with wash buffer containing: 350 mM NaCl, 20 mM Tris-HCl (pH 7.5), and 1% NP-40. Bound proteins were eluted by boiling beads in 30 μL of denaturing sample buffer and analyzed by western blotting.

### Flag–HA–QR2 immunoprecipitation and mass spectrometry

Flag–HA mock control, Flag–HA–tagged wildtype QR2, or Flag–HA–tagged QR2 I129EI195R mutant HeLa cell lines were cultured in DMEM to confluence. Dialyzed nuclear extracts were prepared as described above.

Flag M2 agarose beads were prewashed with 100 mM glycine (pH 2.7) and equilibrated in binding buffer (pH 7.5). For each immunoprecipitation, 50 μL of Flag M2 agarose beads were incubated with nuclear extract for 4 h at 4 °C with rotation. Beads were washed four times with dialysis buffer followed by four washes with detergent-free dialysis buffer. Bound proteins were processed for mass spectrometry using the same protocol described above.

### BNAH fluorescence-based enzymatic activity assay

QR2 enzymatic activity was measured using a fluorescence-based BNAH oxidation assay. Oxidation of the reduced co-substrate 1-benzyl-1,4-dihydronicotinamide (BNAH) (Santa Cruz, sc-208609) by QR2 results in a time-dependent decrease in intrinsic BNAH fluorescence. Reactions were performed in assay buffer containing: 50 mM Tris-HCl (pH8.0) and 0.1% Triton X-100, supplemented with 100 μM Menadione (K3, Sigma, 47775) and BNAH. Reactions were initiated by addition of recombinant QR2 (100 ng per reaction). Control reactions lacking enzyme were included to account for non-enzymatic BNAH decay. Fluorescence was monitored every 2 min using a SpectraMax iD5 plate reader with excitation at 340–355 nm and emission at 440–460 nm. Enzymatic activity was calculated from the linear decrease in fluorescence intensity over time.

### Lentivirus packaging

HEK293T cells were maintained in DMEM supplemented with 10% fetal bovine serum (FBS), 100 U mL^−1^ penicillin, and 100 μg mL^−1^ streptomycin. One day prior to transfection, 10-cm culture dishes were coated with 10 mL of poly-L-ornithine, incubated at 37 °C for 1 h, and washed twice with DPBS. HEK293T cells were dissociated using 0.25% trypsin–EDTA and seeded at a density of 7 × 10^6^ cells per dish in 6 mL of DMEM. After 24 h, the medium was replaced with fresh DMEM and cells were transfected using a polyethylenimine (PEI)-based method. For each dish, a total of 20 μg plasmid DNA (10 μg lentiviral transfer plasmid, 5 μg pMDLg/pRRE, 2.5 μg RSV-Rev, and 2.5 μg pCMV-VSV-G) was diluted in 500 μL DMEM. In parallel, 60 μL of PEI (1 mg mL^−1^) was diluted in 500 μL DMEM. DNA and PEI solutions were combined, incubated for 20 min at room temperature to allow complex formation, and added dropwise to the cultures. Six hours after transfection, cells were washed once with DPBS and fresh, prewarmed DMEM was added. Viral supernatants were collected at 30 and 46 h post-transfection, filtered through a 0.45-μm membrane, and concentrated by ultracentrifugation at 21,000 rpm for 2 h at 4 °C. Viral pellets were resuspended overnight at 4 °C in DMEM/F12 supplemented with HEPES at a volume 100-fold smaller than the original supernatant. An equal volume of 1 M sucrose in DMEM/F12 was then added, and viral stocks were aliquoted and stored at −80 °C.

### Knockout and rescue of QR2 expression in HeLa cells

*NQO2* knockout HeLa cell lines were generated using CRISPR–Cas9–mediated genome editing. Alt-R^™^ S.p. HiFi Cas9 Nuclease V3 (IDT, 1081061) was complexed with an Alt-R CRISPR–Cas9 single-guide RNA (sgRNA) targeting human *NQO2* (5′-CCCACGAAGCCTACAAGCAA-3′; IDT) and delivered into HeLa cells using the SE Cell Line 4D-Nucleofector^™^ X Kit S (Lonza, V4XC-1032) according to the manufacturer’s instructions. Following nucleofection, single-cell clones were isolated and expanded, and *NQO2* knockout was confirmed by western blot analysis. For rescue experiments, the lentiviral backbone pLV-EF1α-IRES-Puro (Addgene #85132) was used to generate expression constructs encoding either Flag–HA–tagged wild-type *NQO2* or the I129EI195R *NQO2* mutant under the control of the endogenous *NQO2* promoter. Lentiviral particles were produced as described above and used to transduce *NQO2*-knockout HeLa cells in DMEM containing 8 μg mL^−1^ polybrene. Cells were incubated at 37 °C with 5% CO_2_ for 24 h, then passaged and selected in DMEM containing 2.0 μg mL^−1^ puromycin. Selection medium was replaced every 3–4 days until resistant colonies were established. Re-expression of wildtype or mutant QR2 was verified by western blot analysis.

### Mice

CD1 mice (Charles River 022) were housed in specific-pathogen-free (SPF) conditions and environmentally controlled animal facilities with a 12-hour light-dark cycle and were given unrestricted access to food and water. All animal protocols and experiments were approved by the Institutional Animal Care and Use Committee (IACUC) of ISMMS. All experiments were performed following strict IACUC guidelines.

### Cerebellar granule neuron culture and depolarization

Cerebellar granule neurons were prepared from postnatal day 7 (P7) CD-1 mouse pups as previously described^76^. Briefly, neurons were cultured *in vitro* in high-potassium medium during days in vitro (DIV) 1-2 consisting of Basal Medium Eagle supplemented with 5% HyClone bovine growth serum, 1 × penicillin–streptomycin, 1 × GlutaMAX and 25 mM KCl. On DIV 3, the medium was replaced with low-potassium medium containing Basal Medium Eagle supplemented with 5% HyClone bovine growth serum, 1 × penicillin–streptomycin, 1 × GlutaMAX, and 5 mM KCl. On DIV 5, neurons were pretreated with 1 μM TTX and 100 μM DL-AP5 for 12 h to suppress spontaneous neuronal activity. For depolarization, cultures were switched to high-potassium medium (25 mM KCl) for 1 h, after which cells were collected for downstream analyses.

### Mouse glial cell preparation

Primary mouse glial cultures were prepared from CD-1 pups (postnatal days P1–P3) in accordance with institutional animal care and use guidelines. Pups were anesthetized by hypothermia and humanely euthanized on ice. Cerebral cortices were dissected in ice-cold HBSS supplemented with 10 mM HEPES, and meninges were carefully removed. Cortical tissue was enzymatically dissociated by incubation with papain (80 μL), activated with 0.5 μM CaCl_2_ and 1 μM EDTA, at 37 °C for 15 min. Following digestion, tissue was gently triturated and passed through a 70 μm cell strainer. Cells were pelleted by centrifugation at 500g for 5 min and resuspended in mouse embryonic fibroblast (MEF) medium consisting of 435 mL DMEM, 50 mL HyClone^™^ Cosmic Calf Serum, 5 mL penicillin–streptomycin, 5 mL sodium pyruvate, 5 mL non-essential amino acids (NEAA), and 4 μL 2-mercaptoethanol. Cells were plated onto 10 cm tissue culture dishes, and the medium was replaced after 24 h. After 5–6 days in culture, glial cells were detached, resuspended in Bambanker freezing medium, and cryopreserved in liquid nitrogen until use.

### Human iPSC maintenance

The WTC11 human pluripotent stem cell (hPSC) line (hPSCreg UCSFi001-A)^46^ was obtained from Coriell Institute for Medical Research, GM25256. Cells were maintained on six-well tissue culture plates coated with Geltrex diluted in DMEM/F-12 and cultured in StemFlex medium. Cultures were maintained at 37 °C in a humidified incubator with 5% CO_2_, with medium changes performed every other day. Cells were passaged at approximately 80% confluency using 0.5 mM EDTA in DPBS at room temperature. Detached cells were resuspended in StemFlex medium supplemented with 5 nM Chromanl and reseeded at split ratios adjusted according to cell density. The following day, the medium was replaced with fresh StemFlex medium lacking Chroman1, and cultures were subsequently maintained using this routine passaging protocol.

### Generation of human *NQO2*, *H3F3B*, and *TGM2* knock-in iPSC lines

Homozygous single- and double-knock-in human iPSC lines were generated in the WTC11 background using CRISPR–Cas9–mediated homology-directed repair (HDR). Briefly, 1 × 10^6^ WTC11 iPSCs were electroporated using a 4D-Nucleofector system (Lonza; program V4XP-3024) with preassembled ribonucleoprotein complexes containing 10 μg S. pyogenes Cas9 nuclease (IDT, 1081058), 8 μg synthetic single-guide RNA (sgRNA; Synthego, CRISPRevolution sgRNA EZ Kit), and 100 pmol single-stranded oligodeoxynucleotide (ssODN) donor template (Life Technologies). Following electroporation, cells were replated on Geltrex-coated plates and cultured in mTeSR Plus medium. Five days after electroporation, cells were dissociated with Accutase and single cells were sorted into 96-well plates using a WOLF benchtop microfluidic cell sorter (Nanocellect) in mTeSR Plus supplemented with CloneR. After 13 days of clonal expansion, genomic DNA was extracted from individual clones using QuickExtract DNA Extraction Solution (Lucigen, QE090500). Correct HDR events were identified by locus-specific PCR using GoTaq DNA Polymerase (Promega) followed by Sanger sequencing.

For *NQO2*, HDR was first used to introduce the I195R mutation in exon 7. Clone MSE2335A was identified as a correctly edited homozygous knock-in clone, whereas clone MSE2335B served as an isogenic wild-type control. Clone MSE2335A was subsequently subjected to a second round of CRISPR–Cas9 editing to introduce the I129E mutation in exon 5, generating a homozygous double-mutant line. For *H3F3B*, HDR was used to introduce the Q5A mutation in exon 2, yielding clone MSE2421A. A separate HDR strategy targeting the same exon was used to generate the Q5N mutation, and clone MSE2422A was used for downstream experiments. For *TGM2*, HDR was used to introduce the C277A mutation in exon 6, and clone MSE2340A was identified as a correctly edited homozygous knock-in line and used for subsequent analyses. Genomic integrity and normal karyotype were confirmed for all edited clones by SNP array analysis. Sequences of sgRNAs, ssODN donor templates, and PCR primers used for CRISPR–Cas9–mediated HDR are provided in Supplementary Data 18.

### Genomic integrity of human iPSC lines

The WTC-11 donor human iPSC line was tested for genomic integrity at passage 36 using SNP-array technology (Global Diversity Array v1.0 BeadChip, Illumina). All the edited clones were also tested at passage 4 after the genome editing. No detection of CNV larger than 1.5 Mb or AOH larger than 3 Mb were detected on somatic chromosomes. The typical WTC-11 deletion of 2.9 Mb on Yp11.2 was detected in all clones. This deletion is known to be present in the donor, from whom the cell line was derived^46^.

### Generation of PiggyBac-integrated hPSC lines

For stable transgene integration, WTC11 hPSCs were seeded at a density of 3–4 × 10^5^ cells per well in six-well tissue culture plates in StemFlex medium supplemented with Chroman1 and cultured overnight. On the following day, cells were transfected with a PiggyBac transgene plasmid encoding doxycycline-inducible human NGN2 (PB-TO-hNGN2; Addgene #172115) together with Super PiggyBac transposase (System Biosciences, PB210PA-1) using Lipofectamine Stem Transfection Reagent. Transfection complexes were prepared by separately diluting 8 μL of Lipofectamine Stem Transfection Reagent in 125 μL Opti-MEM I reduced-serum medium and mixing 500 ng of transposase plasmid and 1 μg of PB-TO-hNGN2 plasmid in 125 μL Opti-MEM I. The two solutions were then combined and incubated for 10 min at room temperature to allow complex formation. A total of 250 μL of the transfection mixture was added dropwise to cells pre-equilibrated in 2 mL of warm Opti-MEM I. Following a 4 h incubation at 37 °C, 2 mL of warm StemFlex medium was added, and cells were cultured overnight. On the subsequent day, the medium was replaced with 1.5 mL of fresh StemFlex medium. Selection was initiated on day 3 by switching to StemFlex medium supplemented with 2 μg mL^−1^ puromycin and maintained for 4–6 days, with medium changes every other day. To ensure uniform selection and eliminate false-positive cells, cultures were passaged once in puromycin-containing StemFlex medium prior to further expansion.

### One-step neuronal induction via forced NGN2 expression

Glutamatergic neurons were generated by doxycycline-inducible overexpression of the transcription factor NGN2, as previously described^77^. PiggyBac–NGN2–integrated WTC11 hPSCs were dissociated using Accutase for 5 min at 37 °C and resuspended in N3 induction medium supplemented with Chroman1 and doxycycline (Dox). N3 induction medium was prepared by combining 485 mL DMEM/F-12, 5 mL N2 supplement, 5 mL non-essential amino acids, 5 mL penicillin–streptomycin, and 10 mg insulin. Cells were counted and seeded at a density of 3 × 10^6^ cells per Geltrex-coated 10-cm dish. Approximately 16–18 h after plating (day 1), the medium was replaced with fresh N3 medium containing Dox. On day 5, the medium was replaced with N3 medium supplemented with Dox and 4 μM cytosine β-D-arabinofuranoside (Ara-C). On day 7, cells were dissociated by incubation with Accutase for 20 min at 37 °C and resuspended in Neurobasal-zero medium supplemented with Chroman1. Neurobasal-zero medium was prepared by combining 483 mL Neurobasal medium, 5 mL GlutaMAX, 10 mL B27 supplement, and 2.5 mL penicillin–streptomycin. Cells were then co-cultured with mouse glial cells in NBP medium and plated onto pre-coated six-well plates. NBP medium consisted of Neurobasal-zero supplemented with 5% fetal bovine serum (FBS), Chroman1, and Dox. On day 8, the medium was replaced with Neurobasal-zero containing 2% FBS (NB-2%). On day 14, cultures were transitioned to Neurobasal-A-zero medium containing 1% FBS (NBA-1%). Neurobasal-A-zero medium was prepared by combining 483 mL Neurobasal-A, 5 mL GlutaMAX, 10 mL B27 supplement, and 2.5 mL penicillin–streptomycin. From day 14 onward, 30% of the medium was replaced twice weekly until day 35, at which time neurons were used for functional and molecular analyses.

### Time-course of neuronal differentiation

For time-course analyses of neuronal differentiation, samples were collected at six developmental stages: undifferentiated hPSCs (day 0), and induced neurons at days 6, 12, 20, 27, and 35 for RNA sequencing. CUT&RUN profiling was performed on undifferentiated hPSCs (day 0) and mature induced neurons at day 35.

### Depolarization treatment

For membrane depolarization experiments, neurons were pretreated with 1 μM TTX and 100 μM DL-AP5 for 12 h to suppress spontaneous neuronal activity. Depolarization was then induced by the addition of a high-potassium buffer (170 mM KCl, 2 mM CaCl_2_, 1 mM MgCl_2_, and 10 mM HEPES) to achieve a final extracellular KCl concentration of 55 mM, as previously described^78^. Untreated control neurons and neurons subjected to 1 h of depolarization were harvested for RNA sequencing.

### Human Subjects

Postmortem human brain tissue samples were obtained from patients who had enrolled in and provided consent for the brain donation program through the Neuropathology Brain Bank & Research CoRE at Mount Sinai, RRID: SCR_027565. These samples were collected according to ethical guidelines and institutional review board (IRB) approval, ensuring the privacy and dignity of the donors while supporting ongoing neuropathological research. All specimens obtained were de-identified. Brain specimens were dissected by expert neuroanatomists into defined blocks. One hemisphere was flash-frozen and stored at −80 °C until processing. Nuclei isolation and peptide pulldown assays were conducted, as previously described.

### Bulk RNA-seq and analysis

#### RNA isolation, library preparation, and sequencing

RNA-seq was performed on HeLa cells and on human iPSC-derived neurons co-cultured with mouse glia across multiple experimental conditions, including genetic perturbations, differentiation stages, and depolarization treatments. All datasets were processed using the same experimental and computational pipelines unless otherwise noted. Cells were lysed in TRIzol reagent (Invitrogen, 15596026), and total RNA was extracted according as follows. Chloroform was added in a 5:1 ratio, and the aqueous phase was isolated. An equal volume of 70% ethanol was added, and the mixture was applied to RNeasy Micro spin columns (Qiagen, 74004). RNA purification was completed following the Qiagen RNeasy Micro Kit protocol, including all optional steps and on-column DNase treatment. RNA was eluted in 15 μL and quantified using a NanoDrop spectrophotometer. Only samples with A260/280 and A260/230 ratios greater than 1.8 were used for downstream applications. For library preparation**,** 100 ng of total RNA was used as input for the Illumina Stranded mRNA Prep, Ligation kit (Illumina, 20040534). Libraries were amplified with 14 cycles of PCR, and amplified fragments were purified using AMPure XP beads (Beckman Coulter, A63881). Library concentration and fragment size distribution were assessed using a Qubit dsDNA HS assay (Invitrogen, Q33231) and a High Sensitivity D5000 TapeStation assay (Agilent Technologies, 5067-5592, 5067-5593). Libraries were pooled at equimolar concentrations and sequenced on an Illumina NovaSeq X+ platform at the NYU Genome Technology Center. HeLa cell libraries were sequenced to a depth of 50–60 million paired-end reads per sample**,** whereas libraries generated from human iPSC-derived neurons co-cultured with mouse glia were sequenced to a depth of approximately 100 million paired-end reads (150 bp) per sample to enable robust species-specific read assignment.

#### Differential expression analysis

For HeLa cell RNA-seq datasets, raw FASTQ files were pseudoaligned and quantified using kallisto (v0.46.1)^79^ against a GRCh38 (hg38) human transcriptome reference derived from Ensembl release 86. For human iPSC-derived neurons co-cultured with mouse glia, reads were pseudoaligned against a combined human–mouse transcriptome reference generated by concatenating Ensembl human (GRCh38, release 86) and mouse (GRCm38/mm10, release 79) transcriptomes**,** and human-mapped transcripts were retained for downstream analyses. Reads corresponding to the human transcriptome were subsequently extracted for downstream analyses. To reduce noise from lowly expressed transcripts, genes with a total read count of fewer than 15 reads across all samples were excluded. Differential gene expression analysis was performed using DESeq2 (v1.38.3)^80^. Gene-level count matrices were normalized using the DESeq2 median-of-ratios method, and differential expression was assessed using the Wald test. *P* values were adjusted for multiple testing using the Benjamini–Hochberg procedure, with an adjusted *P* value < 0.05 considered statistically significant.

#### Gene expression overlap analyses

Transcriptome-wide, threshold-free gene expression overlap was visualized using Rank-Rank Hypergeometric Overlap (RRHO) heatmaps generated with the RRHO2 package (v1.0)^81^. Gene lists were ranked by signed p-values, calculated as the log10-transformed nominal p-value multiplied by the sign of the fold change, without applying differential expression thresholds.

### Chromatin immunoprecipitation

Untreated and depolarization mouse cultured granule neuron cells (1 × 10^7^ cells per sample) were crosslinked with 1% formaldehyde for 10 min at room temperature and quenched with 125 mM glycine for 5 min. Cells were washed thoroughly prior to lysis and chromatin shearing by sonication, as previously described^1^. Sonicated chromatin was incubated overnight at 4 °C with rotation with antibody-conjugated Dynabeads^™^ M-280 (7.5 μg antibody per sample). Antibodies used included anti-H3K4me3Q5ser, anti-H3Q5ser, anti-NQO2, and anti–RNA polymerase II. The following day, immunocomplexes were washed, eluted, and reverse-crosslinked, as previously described^1^. After RNase A and proteinase K digestion, DNA fragments were purified using the QIAquick PCR Purification Kit (QIAGEN, 28104).

### ChIP-seq library preparation and analysis

Purified ChIP DNA was used to prepare sequencing libraries using the NEBNext^®^ Ultra^™^ II DNA Library Prep Kit for Illumina (NEB, E7645L) following the manufacturer’s protocol. Libraries were sequenced as paired-end reads (50 bp) on an Illumina NovaSeq X+ platform to a depth of approximately 30 million reads per sample. ChIP-seq peak calling and differential analyses were performed as previously described, with minor modifications^1^. Raw sequencing reads were aligned to the mouse reference genome (mm10) using the NGS Data Charmer pipeline with default settings (HISAT v0.1.6b)^82^. Peak calling was performed using MACS2 (v2.1.1)^83^, normalized to the corresponding input controls, with a false discovery rate (FDR) threshold of < 0.01. Differential peak analysis was conducted using pairwise comparisons in the DiffBind package (v3.8.4)^84^ to identify depolarization-induced changes in H3Q5ser, H3K4me3Q5ser, NQO2, and RNA polymerase II (Pol II) occupancy. Identified peaks and differentially enriched regions were annotated to nearby genes or intergenic regions using the ChIPseeker^85^ package in R. Visualization of differential binding profiles was performed using internal functions of DiffBind or deepTools^86^.

### CUT&RUN

#### Cells

Hela cells or human iPSC-derived neurons co-cultured with mouse glia cells were resuspended in 1 mL of nuclear extract (NE) buffer (20 mM HEPES-KOH, pH7.9, 10 mM KCl, 0.5 mM spermidine, 0.1% Triton X-100, 20% glycerol and freshly added protease inhibitors (Roche, 11836170001) and passed through a 21-gauge needle 20 times to lyse the cells. Nuclei were pelleted at 1,100*g* for 5 min at 4 °C and the supernatant was discarded. Nuclei were washed again in 1 mL NE buffer and counted. In total, 100,000 nuclei were used per biological replicate.

#### CUT&RUN

CUT&RUN was performed as previously described, with minor modifications^42,87,88^. BioMag Plus Concanavalin A beads (Polysciences, 860573) were prepared by washing 15 μL bead slurry per reaction three times with binding buffer (20 mM HEPES-KOH, pH 7.9, 10 mM KCl, 1 mM CaCl_2_, 1 mM MnCl_2_) and resuspending the beads in the original volume. Beads (15 μL) were aliquoted into DNA low-bind tubes, and 500 μL nuclear extraction buffer was added. A total of 100,000 nuclei were added to each tube and incubated with beads by end-over-end rotation at room temperature for 10 min. Bead-bound nuclei were washed three times with 1 mL wash buffer (WB; 20 mM HEPES, pH 7.5, 150 mM NaCl, 0.1% Triton X-100, 0.1% Tween-20, 0.5 mM spermidine, 0.1% BSA, freshly supplemented with protease inhibitors). Nuclei were then resuspended in 100 μL antibody buffer (wash buffer supplemented with 2 mM EDTA). Primary antibodies (2 μL per reaction; 1:50 dilution) against H3Q5ser, H3K4me3Q5ser, RNA polymerase II, NQO2, POU2F1, or IgG control were added, and bead-bound nuclei were incubated overnight at 4 °C with gentle horizontal rotation with tubes at a 45 degree angle. The following day, nuclei were washed twice with 1 mL cold wash buffer and resuspended in 50 μL cold wash buffer. Protein A/G-MNase (pAG-MNase; Epicypher, 15-1016; 2.5 μL per reaction) was added, and samples were incubated for 1h at 4 °C with the same rotation. Nuclei were subsequently washed three times with 1 mL ice-cold wash buffer, followed by one wash with 1 mL low-salt rinse buffer (20 mM HEPES, pH7.5, 0.5 mM spermidine, 0.1% Tween-20, 0.1% Triton X-100). Bead-bound nuclei were resuspended in ice-cold calcium incubation buffer (3.5 mM HEPES, pH 7.5, 10 mM CaCl_2_, 0.1% Tween-20, 0.1% Triton X-100) and incubated on ice (0 °C) for 30 min for MNase digestion. Reactions were quenched by addition of 100 μL of 2× stop buffer (340 mM NaCl, 20 mM EDTA, 5 mM EGTA, 0.1% Tween-20, 0.1% Triton X-100, 25 μgmL^−1^ RNase A (Thermo Scientific, EN0531), and 0.05 ng per 100 μL *E. coli* spike-in DNA (Epicypher, 18-1401)). Samples were incubated at 37 °C for 30 min without shaking to allow chromatin release and RNA digestion. Beads were captured using a magnetic rack, and the supernatant (200 μL) containing released DNA fragments was collected. DNA was purified using the Zymo ChIP DNA Clean & Concentrator kit (Zymo Research, D5205), eluted in 30 μL, and stored at −20 °C for library preparation.

#### CUT&RUN-seq library preparation and sequencing

Library preparation was performed using the NEBnext Ultra II DNA library kit (E7645L) with multiplexed adapters with minor modifications. CUT&RUN DNA underwent end repair and adapter ligation according to the manufacturer’s protocol (1:15 adapter dilution). DNA was amplified using 16 PCR cycles with an amended 10 s of extension time per cycle to enrich for short fragments. DNA was enriched for 150–350 bp fragments for transcription factor binding events and 150–800 bp for histone markers using Ampure XP beads cleanup. Libraries were quantified with Qubit dsDNA HS assay (Invitrogen, Q33231), and the size distribution was determined by High Sensitivity D5000 TapeStation assay (Agilent Technologies, 5067-5592, 5067-5593). Libraries were pooled at an equimolar concentration and sequenced on the Illumina NovaSeq X+ sequencer by the NYU Genome Technology Center. HeLa cell libraries were sequenced to a depth of 20–30 million paired-end reads per sample**,** whereas libraries generated from human iPSC-derived neurons co-cultured with mouse glia were sequenced to a depth of approximately 50 million paired-end reads (150 bp) per sample to enable robust species-specific read assignment.

#### CUT&RUN–seq data analysis

CUT&RUN data were analyzed as previously described, with minor modifications^88^. For human iPSC-derived neurons co-cultured with mouse glia, reads were aligned using Bowtie2 (v2.5.0)^89^ to a concatenated reference genome comprising the human genome (GRCh38/hg38), the mouse genome (GRCm38/mm10), and the Escherichia coli genome used as a spike-in control. Paired-end reads were aligned using Bowtie2 (v2.5.0) with the --very-sensitive preset and Phred+33 encoding. Insert size parameters were set to 10–2000 bp (-I 10 -X 700), and dovetailing read pairs were permitted (--dovetail). For HeLa cell samples, reads were aligned to a concatenated reference consisting of GRCh38/hg38 and the *E. coli* genome. Aligned reads were filtered using SAMtools (v1.9)^90^ to retain only uniquely mapped reads with a minimum mapping quality (MAPQ) score of 30. Reads were subsequently separated by species (human, mouse, and *E. coli*) for downstream analyses. For normalization and quantitative comparisons across samples, *E. coli* spike-in DNA was used to calculate a scaling factor for each sample, defined as the median number of uniquely mapped *E. coli* reads across all samples divided by the number of *E. coli* reads in the given sample. This scaling factor was applied to normalize human-aligned read counts and genome coverage tracks. Genome coverage tracks were generated as bigWig files using the deepTools (v3.5.5)^91^ bamCoverage function, and regions with consistently high background signal were excluded using the ENCODE hg38 blacklist (v2). Heatmaps were generated using deepTools (v3.5.5) computeMatrix and plotHeatmap functions in reference-point mode, centered on peak centers. Peak calling was performed using MACS2^83^ with a false discovery rate (FDR) threshold of < 0.01. Differential peak analysis was conducted using DiffBind^84^ by quantifying read counts across consensus peak regions and statistically testing for condition-dependent differences in chromatin occupancy. Identified peaks and differentially enriched regions were annotated, and motif enrichment analyses were performed using the ChIPseeker^85^ package in R.

### ATAC–seq

#### Nuclei preparation

Nuclei from induced neurons (iNs) were prepared using the same nuclei isolation procedure as described for CUT&RUN. For each biological replicate, 100,000 nuclei were used as input for ATAC–seq library preparation.

#### ATAC-seq library preparation

ATAC-seq libraries were generated using the ATAC-Seq Express Kit (Active Motif, 53157) according to the manufacturer’s instructions. Libraries were size-selected using SPRIselect Bead-Based Reagent (Beckman, B23318) to enrich for fragments ranging from 200 to 800 bp. Library quality and fragment size distribution were assessed using a High Sensitivity D5000 TapeStation assay (Agilent Technologies; 5067-5592, 5067-5593). Libraries were pooled at equimolar concentrations and sequenced as 150-bp paired-end reads on an Illumina NovaSeq X+ platform at the NYU Genome Technology Center, yielding approximately 60 million reads per sample.

#### ATAC-seq data processing and analysis

Primary ATAC-seq data processing included adapter trimming, alignment to a concatenated human–mouse reference genome comprising the human genome (GRCh38/hg38) and the mouse genome (GRCm38/mm10) using Bowtie2^89^, and removal of mitochondrial reads and PCR duplicates. Peak calling was performed independently for each of the three biological replicates using MACS2^83^, followed by assessment of reproducibility across replicates. Normalization and differential chromatin accessibility analyses were conducted using DiffBind^84^. Transcription factor footprinting analysis was performed using TOBIAS^92^, which corrects ATAC–seq signal for Tn5 transposase sequence bias and local chromatin accessibility prior to footprint inference. Differential transcription factor binding activity was quantified using the BINDetect module by comparing footprint depth and flanking accessibility at motif instances, enabling conditionspecific comparison of POU2F1 binding scores between wild-type and mutant samples. To assess whether loss of chromatin accessibility in mutant neurons was associated with loss of POU2F1 (OCT1) binding motifs, we performed motif scanning of ATAC-seq peaks using POU2F1 consensus motifs from the JASPAR database (MA0785.1 and MA0785.2). Genomic coordinates of ATAC-seq peaks were scanned for motif occurrences to classify loci as OCT1 motif–positive (OCT1+) or OCT1 motif–negative (OCT1-). Differential accessibility analysis was used to identify ATAC peaks showing reduced accessibility in mutant compared with wild-type hiPSC-derived neurons.

### Odds ratio analysis integrating RNA-seq and CUT&RUN

To quantify the association between transcriptional changes and chromatin occupancy, an odds ratio (OR) framework was used to integrate RNA-seq and CUT&RUN datasets. Differential gene expression was assessed for each mutant relative to wild-type (WT) using DESeq2^80^, and differentially expressed genes (DEGs) were defined as genes with an adjusted P value < 0.05 and an absolute log_2_ fold-change ≥0.5.

CUT&RUN peaks were called per biological replicate using MACS2^83^, and differential binding between WT and mutant conditions was assessed using DiffBind^84^. For each CUT&RUN dataset (NQO2, H3K4me3Q5ser, or RNA polymerase II), WT-enriched CUT&RUN peaks were annotated to genes using ChIPseeker^85^ by assigning peaks to the nearest transcription start site (TSS), with promoter regions defined as ±3 kb around the TSS (tssRegion = −3,000 to +3,000). Annotations were performed using TxDb.Hsapiens.UCSC.hg38.knownGene together with the org.Hs.eg.db database. Genes associated with at least one WT-enriched CUT&RUN peak within this window were classified as “bound.” For each mutant and each CUT&RUN dataset, enrichment was tested using a 2*2 contingency table constructed across the expressed gene universe (genes passing RNA-seq filtering), stratified by (i) DEG status (DEG vs non-DEG) and (ii) CUT&RUN association (bound vs unbound). Odds ratios, 95% confidence intervals, and two-sided P values were computed using Fisher’s exact test^93^. Odds ratios greater than 1 indicate enrichment of DEGs among genes associated with WT-enriched CUT&RUN signal. Where applicable, P values were adjusted for multiple testing as indicated.

### Analysis of dendritic arborizations

Immunofluorescence imaging was performed on mature induced neurons (iNs) following sparse labeling with an hSYN-EGFP expression plasmid introduced by calcium phosphate–mediated transfection. Images were acquired using an EVOS^™^ M5000 Imaging System equipped with a 20× objective. For each condition, 20 EGFP-positive neurons per biological replicate were analyzed using FIJI. Quantitative morphological analyses included total neurite outgrowth, defined as the summed length of all neurites per neuron; the number of processes, defined as primary neurites emerging from the soma; neurite branch points; and cell body area.

### Immunofluorescence staining

Cells were washed with DPBS and fixed with 4% paraformaldehyde (PFA) for 5 min at room temperature. Following three washes with DPBS, cells were permeabilized with 0.2% Triton X-100 for 5–10 min at room temperature. Non-specific binding was blocked for 2 h at room temperature in blocking buffer consisting of DPBS supplemented with 4% bovine serum albumin (BSA; Sigma, A9647), 1% fetal bovine serum (FBS), and 0.02% sodium azide (Sigma, 71289). For human iPSC-derived neurons, cells were incubated overnight at 4 °C with primary antibodies diluted in blocking buffer, including anti-MAP2 (1:20,000), anti-Synapsin I/II (1:500), and anti-PSD95 (1:500). For cultured mouse cerebellar granule neurons, cells were incubated overnight at 4 °C with anti-NQO2 (1:1,000) and anti-H3K4me3Q5ser (1:1,000). For mouse brain slices, tissues were incubated with anti-NQO2 under the same conditions. After three washes with DPBS, cells or tissue sections were incubated for 1 h at room temperature in the dark with species-specific secondary antibodies diluted in blocking buffer: Alexa Fluor 488–conjugated anti-chicken IgY (H+L), Alexa Fluor 555–conjugated anti-rabbit IgG (H+L), and Alexa Fluor 647–conjugated anti–guinea pig IgG (H+L) (all at 1:1,000). Samples were washed three times with DPBS, and nuclei were counterstained with DAPI (0.2 ng mL^−1^; Thermo Fisher Scientific, 62248) during the final wash. Coverslips were mounted using ProLong Gold Antifade Mountant with DAPI (Invitrogen, P36941).

### Synaptic puncta density analysis

Immunofluorescence analyses were performed on mature induced neurons stained for microtubule-associated protein 2 (MAP2) to label dendrites and either Synapsin I/II (SYN1) or PSD95 to label pre- or post-synaptic puncta, respectively. Confocal images were acquired using a Leica SP8 inverted confocal microscope equipped with a 40× oil-immersion objective. Z-stack images were collected from ten randomly selected fields per condition, converted to maximum-intensity projections, and analyzed using FIJI software. Cell bodies were masked prior to analysis. Synaptic puncta number was normalized to the total MAP2-positive area to calculate puncta density, or to the mean MAP2 fluorescence intensity to calculate puncta intensity. In addition, puncta size and fluorescence intensity were quantified.

### Colocalization analysis

Synaptic colocalization was assessed between presynaptic SYN1 and postsynaptic density protein 95 (PSD95). To minimize background and non-synaptic signal, particle size thresholds were applied in FIJI prior to analysis. SYN1-positive puncta were segmented using a size range of 0.05–3 μm^2^, whereas PSD95-positive puncta were segmented using a size range of 0.05–1 μm^2^. Following thresholding and size filtering, binary masks were generated for each channel, and colocalized puncta were defined as pixels positive for both SYN1 and PSD95. The degree of synaptic colocalization was quantified using Manders’ overlap coefficient, calculated as the fraction of double-positive signal. Identical acquisition settings, thresholding parameters, and size filters were applied across all experimental conditions.

### Multielectrode array (MEA) recordings

Neuronal network activity was recorded using the Maestro Pro multielectrode array system (Axion Biosystems). Recordings were performed for 10 min per well under controlled conditions (37 °C, 5% CO_2_). Signals were acquired at a sampling rate of 12.5 kHz per channel and filtered using a Butterworth band-pass filter (200–3,000 Hz). Both positive- and negative-deflecting spikes were detected using an adaptive threshold set at six times the standard deviation of the baseline noise. Stimulation and pharmacological assays were primarily conducted at day 35 of neuronal differentiation. For inhibitor experiments, baseline activity was recorded prior to compound addition. Inhibitors were prepared as 10× stock solutions and rapidly added to the existing culture medium at 1/9 of the total volume to achieve the desired final concentration (10 μM). Stock solutions were prepared in DMSO, with the final DMSO concentration maintained below 0.1%. Following compound addition, neuronal activity was recorded for an additional 10 min. MEA data analysis was performed using the Neural Metrics Tool software package (Axion Biosystems).

## Extended Data

**Extended Data Table 1:** ITC statistics

NO.	Proteins	Ligands	deltaH (cal/mol)	deltaS (cal/mol/deg)	Ka (M-1)	N value	Relevant Figure Panel
1	QR2	H3(1-10)	/	/	/	/	Figure 1h
2	QR2	H3(1-10)K4me3	/	/	/	/	Figure 1h
3	QR2	H3(1-10)Q5ser	−7307±47.26	0.726	3.25E5±1.60E4	0.840±0.00403	Figure 1h
4	QR2	H3(1-10)K4me3Q5ser	−5831±81.06	5.71	3.35E5±2.65E4	1.00±0.0074	Figure 1h
5	QR2	H3(1-15)Q5his	/	/	/	/	Figure 1i
6	QR2	H3(1-15)Q5dop	/	/	/	/	Figure 1i
7	QR2	H3(1-10)Q5ser	−6.65 ± 0.06	4.18	1.63 ± 0.16	1.04 ± 0.01	Extended Data Figure 2c
8	QR2 (apo)	H3(1-10)Q5ser	/	/	/	/	Extended Data Figure 2c
9	QR2-Q122A	H3(1-10)Q5ser	−4.31 ± 0.08	6.07	32.7 ± 1.4	0.93 ± 0.01	Extended Data Figure 4a
10	QR2-F126A	H3(1-10)Q5ser	−2.51 ± 0.11	10.4	79.4 ± 9.2	1.00 ± 0.00	Extended Data Figure 4a
11	QR2-F178A	H3(1-10)Q5ser	−3.21 ± 0.05	9.27	41.8 ± 1.5	1.03 ± 0.01	Extended Data Figure 4a
12	QR2-Q122A/F126A/F178A	H3(1-10)Q5ser	/	/	/	/	Extended Data Figure 4a
13	QR2 I129EI195R	H3(1-10)Q5ser	−1.116E4±72.20	−8.41	2.21E6±1.03E5	0.626±0.00143	Extended Data Figure 5a
14	QR2 I129EI195R	5-HT	−1.118E4±236.9	−13.5	1.73E5±8.78E3	0.233±0.00378	Extended Data Figure 5b
15	QR2 I129EI195R	H3(1-10)Q5ser	−1.216E7±1.188E10	−4.07E+04	7.93E3±2.85E3	5.73E-4±0.560	Extended Data Figure 5a
16	QR2 I129EI195R	5-HT	−1.153E4±272.5	−14.4	1.97E5±1.71E4	0.362±0.00635	Extended Data Figure 5b

**Extended Data Table 2:** X-ray crystallography data collection and refinement statistics

Crystal	QR2-H3Q5ser	QR2-H3K4me3Q5ser
**Data collection**		
Wavelength(Å)	0.9792	0.9792
Space group	P4_3_2_1_2	P4_3_2_1_2
Cell dimensions		
a, b, c (Å)	119.0, 119.0, 149.8	119.9, 119.9, 150.2
α, β, γ (°)	90, 90, 90	90, 90, 90
Resolution (Å)	50 - 2.40 (2.44 - 2.40)	50 - 2.59 (2.63 - 2.59)
No. of reflections	42961 (2094)	34824 (1749)
R_merge_ (%)	20.0 (98.3)	21.1 (120.6)
R_pim_ (%)	4.0 (20.0)	7.1 (42.7)
I/σ (I)	20.0 (3.0)	14.3 (2.6)
CC1/2 (%)	99.5 (89.4)	99.1 (77.5)
Completeness (%)	100.0 (100.0)	99.6 (100.0)
Redundancy	25.8 (24.8)	9.6 (8.4)
**Refinement (F>0)**		
R_work_/R_free_ (%)	16.3/21.2	15.5/20.2
No. of non-H atoms		
Protein	7290	7285
Peptide	300	300
Water	472	315
Average B-factor (Å^2^)		
Protein	40.1	36.5
Peptide	38.9	33.7
Water	38.7	32.8

## Supplementary Material

Supplement 1 **Supplementary Data 1:** LC-MS/MS analysis from HeLa cell NEs.

Supplement 2 **Supplementary Data 2:** LC-MS/MS analysis from SK-N-SH cell lysates.

Supplement 3 **Supplementary Data 3:** CUT&RUN-seq from wildtype HeLa cells (H3K4me3Q5ser, QR2, RNA Pol II)

Supplement 4

Supplement 5 **Supplementary Data 4:** CUT&RUN-seq from wildtype *vs.* complementation HeLa cells (H3K4me3Q5ser, QR2, RNA Pol II)

Supplement 6 **Supplementary Data 5:** RNA-seq from wildtype *vs.* complementation HeLa cells

Supplement 7 **Supplementary Data 6:** ChIP-seq from mouse cGNs −/+ KCl (H3K4me3Q5ser, H3Q5ser, QR2, RNA PolII)

Supplement 8 **Supplementary Data 7:** RNA-seq from hiPSC neurons (time course)

Supplement 9 **Supplementary Data 8:** CUT&RUN-seq from hiPSC neurons (DIV0/DIV35; H3K4me3Q5ser, QR2)

Supplement 10 **Supplementary Data 9:** Gene ontologies related to Extended Data Figure 8h

Supplement 11 **Supplementary Data 10:** CUT&RUN-seq from wildtype *vs.* mutant hiPSC neurons (H3K4me3Q5ser, QR2, RNA Pol II)

Supplement 12 **Supplementary Data 11:** RNA-seq from wildtype *vs.* mutant hiPSC neurons

Supplement 13 **Supplementary Data 12:** 1,193 Overlapping genes displaying regulation in mutant *vs.* wildtype hiPSC neurons

Supplement 14 **Supplementary Data 13:** Gene ontologies related to Fig. 2n

Supplement 15 **Supplementary Data 14:** LC-MS/MS analysis from HeLa NEs (FLAG-HA-tagged QR2 wildtype *vs.* Flag-HA mock; FLAG-HA-tagged QR2 I129EI195R *vs.* FLAG-HA-tagged QR2 wildtype)

Supplement 16 **Supplementary Data 15:** CUT&RUN-seq from wildtype *vs.* mutant hiPSC neurons (POU2F1)

Supplement 17 **Supplementary Data 16:** ATAC-seq from wildtype *vs.* mutant hiPSC neurons

Supplement 18

Supplement 19

Supplement 20

Supplement 21 **Supplementary Data 17:** Gene ontologies related to Fig. 4j

Supplement 22 **Supplementary Data 18:** Sequences of sgRNAs, ssODN donor templates, and PCR primers used for CRISPR–Cas9–mediated HDR

23

## Data Availability

The genomics data generated in this study have been deposited in the National Center for Biotechnology Information Gene Expression Omnibus (GEO) database under the SuperSeries GSE324882. All mass spectrometry-based proteomics data have been deposited to the ProteomeXchange Consortium via the PRIDE partner repository with the datasets identified as PXD075254 and PXD075547. The atomic coordinates and structure factors have been deposited in the Protein Data Bank (PDB) under PDB ID codes 24PZ and 24RO. We declare that the data supporting findings for this study are available within the article and Supplementary Information. Related data including raw microscopy images are available from the corresponding author upon reasonable request. No restrictions on data availability apply.

## CODE AVAILABILITY

Related code is available from the corresponding author on reasonable request.

## References

[1] Farrelly L. A. Histone serotonylation is a permissive modification that enhances TFIID binding to H3K4me3. Nature 567, 535–539 (2019). 10.1038/s41586-019-1024-7 30867594 PMC6557285

[2] Al-Kachak A. & Maze I. Post-translational modifications of histone proteins by monoamine neurotransmitters. Current Opinion in Chemical Biology 74, 102302 (2023). 10.1016/j.cbpa.2023.102302 37054563 PMC10225327

[3] Vinson D. A. & Maze I. Reimagining biogenic amine signaling in the brain and beyond. Trends Neurosci 49, 35–48 (2026). 10.1016/j.tins.2025.11.003 41387034 PMC12704813

[4] Weekley B. H., Ahmed N. I. & Maze I. Elucidating neuroepigenetic mechanisms to inform targeted therapeutics for brain disorders. iScience 28, 112092 (2025). 10.1016/j.isci.2025.112092 40160416 PMC11951040

[5] Chan J. C. & Maze I. Nothing Is Yet Set in (Hi)stone: Novel Post-Translational Modifications Regulating Chromatin Function. Trends Biochem Sci 45, 829–844 (2020). 10.1016/j.tibs.2020.05.009 32498971 PMC7502514

[6] Sardar D. Induction of astrocytic Slc22a3 regulates sensory processing through histone serotonylation. Science 380, eade0027 (2023). 10.1126/science.ade0027 37319217 PMC10874521

[7] Zheng Q. Bidirectional histone monoaminylation dynamics regulate neural rhythmicity. Nature 637, 974–982 (2025). 10.1038/s41586-024-08371-3 39779849 PMC11754111

[8] Al-Kachak A. Histone serotonylation in dorsal raphe nucleus contributes to stress- and antidepressant-mediated gene expression and behavior. Nat Commun 15, 5042 (2024). 10.1038/s41467-024-49336-4 38871707 PMC11176395

[9] Chan J. C. Serotonin Transporter-dependent Histone Serotonylation in Placenta Contributes to the Neurodevelopmental Transcriptome. JMol Biol 436, 168454 (2024). 10.1016/j.jmb.2024.168454 38266980 PMC10957302

[10] Chen H. C. Histone serotonylation regulates ependymoma tumorigenesis. Nature 632, 903–910 (2024). 10.1038/s41586-024-07751-z 39085609 PMC11951423

[11] Zhang N. Bioorthogonal Labeling and Enrichment of Histone Monoaminylation Reveal Its Accumulation and Regulatory Function in Cancer Cell Chromatin. J Am Chem Soc (2024). 10.1021/jacs.4c04249 PMC13077737 38848464

[12] Ling T. Serotonylation in tumor-associated fibroblasts contributes to the tumor-promoting roles of serotonin in colorectal cancer. Cancer Lett 600, 217150 (2024). 10.1016/j.canlet.2024.217150 39097134

[13] Zhang N. pH-Controlled Chemoselective Rapid Azo-Coupling Reaction (CRACR) Enables Global Profiling of Serotonylation Proteome in Cancer Cells. J Proteome Res 23, 4457–4466 (2024). 10.1021/acs.jproteome.4c00409 39208062 PMC13112263

[14] Dong R. TGM2-mediated histone serotonylation promotes HCC progression via MYC signalling pathway. J Hepatol 83, 105–118 (2025). 10.1016/j.jhep.2024.12.038 39788430

[15] Liu K. 5-HT orchestrates histone serotonylation and citrullination to drive neutrophil extracellular traps and liver metastasis. J Clin Invest 135 (2025). 10.1172/JCI183544 PMC11996869 39903533

[16] Navarro-Corcuera A. & Martinez-Chantar M. L. Histone serotonylation in HCC: Decoding the impact of “happy” histones on liver cancer progression. J Hepatol 83, 18–20 (2025). 10.1016/j.jhep.2025.02.020 40023196

[17] Lin S. Histone serotonylation promotes pancreatic cancer development via lipid metabolism remodeling. Nat Commun 16, 5947 (2025). 10.1038/s41467-025-61197-z 40593695 PMC12217961

[18] Ji Y. Serotonin modulates lineage plasticity in neuroendocrine prostate cancer via epigenetic reprogramming. Cancer Discov (2025). 10.1158/2159-8290.CD-25-0974 PMC13040212 41416996

[19] Li A. 5-HT reuptake blockade induces pyroptosis in BRAF(V600E)-mutated melanomas via remodeling histone serotonylation. Cell Rep Med 7, 102537 (2026). 10.1016/j.xcrm.2025.102537 41494533 PMC12866116

[20] Pulido-Cortes L. Molecular determinants for recognition of serotonylated chromatin. Nucleic Acids Res 53 (2025). 10.1093/nar/gkaf612 PMC12238850 40637225

[21] Zhao S. Histone H3Q5 serotonylation stabilizes H3K4 methylation and potentiates its readout. Proc Natl Acad Sci U S A 118 (2021). 10.1073/pnas.2016742118 PMC8017887 33526675

[22] Dincer E., Oguz M. & Baris Y. Biochemical and pharmacological properties of biogenic amines. Biogenic Amines (2018). 10.5772/intechopen.81569

[23] Fukumoto T., Kema I. P. & Levin M. Serotonin signaling is a very early step in patterning of the left-right axis in chick and frog embryos. Current Biology 15, 794–803 (2005). 10.1016/j.cub.2005.03.044 15886096

[24] Neumann J., Hofmann B., Dhein S. & Gergs U. Cardiac roles of serotonin (5-HT) and 5-HT-receptors in health and disease. Int J Mol Sci 24 (2023). 10.3390/ijms24054765 PMC10003731 36902195

[25] Cirillo C., Vanden Berghe P. & Tack J. Role of serotonin in gastrointestinal physiology and pathology. Minerva Endocrinol 36, 311–324 (2011). 22322654

[26] Teleanu R. I. Neurotransmitters-key factors in neurological and neurodegenerative disorders of the central nervous system. Int J Mol Sci 23 (2022). 10.3390/ijms23115954 PMC9180936 35682631

[27] Yang X. Pathophysiologic role of neurotransmitters in digestive diseases. Front Physiol 12, 567650 (2021). 10.3389/fphys.2021.567650 34194334 PMC8236819

[28] Walther D. J., Stahlberg S. & Vowinckel J. Novel roles for biogenic monoamines: from monoamines in transglutaminase-mediated post-translational protein modification to monoaminylation deregulation diseases. FEBS J 278, 4740–4755 (2011). 10.1111/j.1742-4658.2011.08347.x 21923757

[29] Walther D. J. Serotonylation of small GTPases is a signal transduction pathway that triggers platelet alpha-granule release. Cell 115, 851–862 (2003). 10.1016/s0092-8674(03)01014-6 14697203

[30] Lepack A. E. Dopaminylation of histone H3 in ventral tegmental area regulates cocaine seeking. Science 368, 197–201 (2020). 10.1126/science.aaw8806 32273471 PMC7228137

[31] Stewart A. F., Lepack A. E., Fulton S. L., Safovich P. & Maze I. Histone H3 dopaminylation in nucleus accumbens, but not medial prefrontal cortex, contributes to cocaine-seeking following prolonged abstinence. Mol Cell Neurosci 125, 103824 (2023). 10.1016/j.mcn.2023.103824 36842545 PMC10247417

[32] Fulton S. L. Histone H3 dopaminylation in ventral tegmental area underlies heroin-induced transcriptional and behavioral plasticity in male rats. Neuropsychopharmacology 47, 1776–1783 (2022). 10.1038/s41386-022-01279-4 35094023 PMC9372029

[33] Lukasak B. J. TGM2-mediated histone transglutamination is dictated by steric accessibility. Proc Natl Acad Sci USA 119, e2208672119 (2022). 10.1073/pnas.2208672119 36256821 PMC9618071

[34] Zhao J. Structural insights into the recognition of histone H3Q5 serotonylation by WDR5. Sci Adv 7 (2021). 10.1126/sciadv.abf4291 PMC8213231 34144982

[35] Vella F., Ferry G., Delagrange P. & Boutin J. A. NRH:quinone reductase 2: an enzyme of surprises and mysteries. Biochem Pharmacol 71, 1–12 (2005). 10.1016/i.bcp.2005.09.019 16253210

[36] Ferry G. & Boutin J. A. Measurement of NQO2 Catalytic Activity and of Its Inhibition by Melatonin. Methods Mol Biol 2550, 315–321 (2022). 10.1007/978-1-0716-2593-4_33 36180702

[37] Calamini B., Santarsiero B. D., Boutin J. A. & Mesecar A. D. Kinetic, thermodynamic and X-ray structural insights into the interaction of melatonin and analogues with quinone reductase 2. Biochem J 413, 81–91 (2008). 10.1042/BJ20071373 18254726 PMC3265362

[38] Calamini B., Ferry G. & Boutin J. A. Cloning, Expression, Purification, Crystallization, and X-Ray Structural Determination of the Human NQO2 in Complex with Melatonin. Methods Mol Biol 2550, 291–304 (2022). 10.1007/978-1-0716-2593-4_31 36180700

[39] Janda E., Boutin J. A., De Lorenzo C. & Arbitrio M. Polymorphisms and Pharmacogenomics of NQO2: The Past and the Future. Genes (Basel) 15 (2024). 10.3390/genes15010087 PMC10815803 38254976

[40] Benoit C. E. Loss of quinone reductase 2 function selectively facilitates learning behaviors. J Neurosci 30, 12690–12700 (2010). 10.1523/JNEUROSCI2808-10.2010 20861374 PMC6633588

[41] Lin J., Bao X. & Li X. D. A tri-functional amino acid enables mapping of binding sites for posttranslational-modification-mediated protein-protein interactions. Mol Cell 81, 2669–2681 e2669 (2021). 10.1016/i.molcel.2021.04.001 33894155

[42] Skene P. J. & Henikoff S. An efficient targeted nuclease strategy for high-resolution mapping of DNA binding sites. Elife 6 (2017). 10.7554/eLife.21856 PMC5310842 28079019

[43] Maze I. Critical Role of Histone Turnover in Neuronal Transcription and Plasticity. Neuron 87, 77–94 (2015). 10.1016/i.neuron.2015.06.014 26139371 PMC4491146

[44] Gould N. L. Specific quinone reductase 2 inhibitors reduce metabolic burden and reverse Alzheimer’s disease phenotype in mice. J Clin Invest 133 (2023). 10.1172/ici162120 PMC10541198 37561584

[45] Zhang Y. Rapid single-step induction of functional neurons from human pluripotent stem cells. Neuron 78, 785–798 (2013). 10.1016/i.neuron.2013.05.029 23764284 PMC3751803

[46] Kreitzer F. R. A robust method to derive functional neural crest cells from human pluripotent stem cells. Am J Stem Cells 2, 119–131 (2013). 23862100 PMC3708511

[47] Funk O. H., Qalieh Y., Doyle D. Z., Lam M. M. & Kwan K. Y. Postmitotic accumulation of histone variant H3.3 in new cortical neurons establishes neuronal chromatin, transcriptome, and identity. Proc Natl Acad Sci US A 119, e2116956119 (2022). 10.1073/pnas.2116956119 PMC9371731 35930666

[48] Domcke S. A human cell atlas of fetal chromatin accessibility. Science 370 (2020). 10.1126/science.aba7612 PMC7785298 33184180

[49] Kiyota T., Kato A., Altmann C. R. & Kato Y. The POU homeobox protein Oct-1 regulates radial glia formation downstream of Notch signaling. Dev Biol 315, 579–592 (2008). 10.1016/j.ydbio.2007.12.013 18241856

[50] Rada B. & Leto T. L. Oxidative innate immune defenses by Nox/Duox family NADPH oxidases. Contrib Microbiol 15, 164–187 (2008). 10.1159/000136357 18511861 PMC2776633

[51] Rowe L. A., Degtyareva N. & Doetsch P. W. DNA damage-induced reactive oxygen species (ROS) stress response in Saccharomyces cerevisiae. Free Radic Biol Med 45, 1167–1177 (2008). 10.1016/j.freeradbiomed.2008.07.018 18708137 PMC2643028

[52] Shields H. J., Traa A. & Van Raamsdonk J. M. Beneficial and Detrimental Effects of Reactive Oxygen Species on Lifespan: A Comprehensive Review of Comparative and Experimental Studies. Front Cell Dev Biol 9, 628157 (2021). 10.3389/fcell.2021.628157 33644065 PMC7905231

[53] Deller S., Macheroux P. & Sollner S. Flavin-dependent quinone reductases. Cell Mol Life Sci 65, 141–160 (2008). 10.1007/s00018-007-7300-y 17938860 PMC11131675

[54] Islam F., Leung K. K., Walker M. D., Al Massri S. & Shilton B. H. The Unusual Cosubstrate Specificity of NQO2: Conservation Throughout the Amniotes and Implications for Cellular Function. Front Pharmacol 13, 838500 (2022). 10.3389/fphar.2022.838500 35517822 PMC9065289

[55] Gould N. L. Specific quinone reductase 2 inhibitors reduce metabolic burden and reverse Alzheimer’s disease phenotype in mice. J Clin Invest 133 (2023). 10.1172/JCI162120 PMC10541198 37561584

[56] Islam F., Basilone N., Yoo V., Ball E. & Shilton B. Evolutionary analysis of Quinone Reductases 1 and 2 suggests that NQO2 evolved to function as a pseudoenzyme. Protein Sci 33, e5234 (2024). 10.1002/pro.5234 39584664 PMC11586865

[57] Brouillette J. & Quirion R. Transthyretin: a key gene involved in the maintenance of memory capacities during aging. Neurobiol Aging 29, 1721–1732 (2008). 10.1016/j.neurobiolaging.2007.04.007 17512093

[58] Gould N. L., Elkobi A., Edry E., Daume J. & Rosenblum K. Muscarinic-Dependent miR-182 and QR2 Expression Regulation in the Anterior Insula Enables Novel Taste Learning. eNeuro 7 (2020). 10.1523/ENEURQ.0067-20.2020 PMC7266141 32217627

[59] Hashimoto T. & Nakai M. Increased hippocampal quinone reductase 2 in Alzheimer’s disease. Neurosci Lett 502, 10–12 (2011). 10.1016/j.neulet.2011.07.008 21803122

[60] Rappaport A. N. Expression of Quinone Reductase-2 in the Cortex Is a Muscarinic Acetylcholine Receptor-Dependent Memory Consolidation Constraint. J Neurosci 35, 15568–15581 (2015). 10.1523/JNEURQSCI1170-15.2015 26609153 PMC6705469

[61] Takeuchi T. Locus coeruleus and dopaminergic consolidation of everyday memory. Nature 537, 357–362 (2016). 10.1038/nature19325 27602521 PMC5161591

[62] Cai D. & Liu T. Inflammatory cause of metabolic syndrome via brain stress and NF-kappaB. Aging (Albany NY) 4, 98–115 (2012). 10.18632/aging.100431 22328600 PMC3314172

[63] Muddapu V. R., Dharshini S. A. P., Chakravarthy V. S. & Gromiha M. M. Neurodegenerative Diseases - Is Metabolic Deficiency the Root Cause? Front Neurosci 14, 213 (2020). 10.3389/fnins.2020.00213 32296300 PMC7137637

[64] Vogel S. & Schwabe L. Learning and memory under stress: implications for the classroom. NPJ Sci Learn 1, 16011 (2016). 10.1038/npjscilearn.2016.11 30792896 PMC6380371

[65] Gould N. L. Dopamine-Dependent QR2 Pathway Activation in CA1 Interneurons Enhances Novel Memory Formation. J Neurosci 40, 8698–8714 (2020). 10.1523/JNEUR0SCI.1243-20.2020 33046554 PMC7643296

[66] Gould N. L., Kolatt Chandran S., Kayyal H., Edry E. & Rosenblum K. Somatostatin Interneurons of the Insula Mediate QR2-Dependent Novel Taste Memory Enhancement. eNeuro 8 (2021). 10.1523/ENEUR0.0152-21.2021 PMC8482851 34518366

[67] Iskander K. & Jaiswal A. K. Quinone oxidoreductases in protection against myelogenous hyperplasia and benzene toxicity. Chem Biol Interact 153-154, 147–157 (2005). 10.1016/j.cbi.2005.03.019 15935811

[68] Long D. J. 2nd Disruption of dihydronicotinamide riboside:quinone oxidoreductase 2 (NQO2) leads to myeloid hyperplasia of bone marrow and decreased sensitivity to menadione toxicity. J Biol Chem 277, 46131–46139 (2002). 10.1074/jbc.M208675200 12351651

[69] Celli C. M., Tran N., Knox R. & Jaiswal A. K. NRH:quinone oxidoreductase 2 (NQO2) catalyzes metabolic activation of quinones and anti-tumor drugs. Biochem Pharmacol 72, 366–376 (2006). 10.1016/j.bcp.2006.04.029 16765324

[70] Tyanova S., Temu T. & Cox J. The MaxQuant computational platform for mass spectrometry-based shotgun proteomics. Nat Protoc 11, 2301–2319 (2016). 10.1038/nprot.2016.136 27809316

[71] Vagin A. & Teplyakov A. Molecular replacement with MOLREP. Acta Crystallogr D Biol Crystallogr 66, 22–25 (2010). 10.1107/S0907444909042589 20057045

[72] Emsley P. & Cowtan K. Coot: model-building tools for molecular graphics. Acta Crystallogr D Biol Crystallogr 60, 2126–2132 (2004). 10.1107/S0907444904019158 15572765

[73] Adams P. D. PHENIX: a comprehensive Python-based system for macromolecular structure solution. Acta Crystallogr D Biol Crystallogr 66, 213–221 (2010). 10.1107/S0907444909052925 20124702 PMC2815670

[74] Flood D. T. Leveraging the Knorr Pyrazole Synthesis for the Facile Generation of Thioester Surrogates for use in Native Chemical Ligation. Angew Chem Int Ed Engl 57, 11634–11639 (2018). 10.1002/anie.201805191 29908104 PMC6126375

[75] Simon M. D. The site-specific installation of methyl-lysine analogs into recombinant histones. Cell 128, 1003–1012 (2007). 10.1016/j.cell.2006.12.041 17350582 PMC2932701

[76] Bilimoria P. M. & Bonni A. Cultures of cerebellar granule neurons. CSH Protoc 2008, pdb.prot5107 (2008). 10.1101/pdb.prot5107 PMC2996826 21356753

[77] Zhang Y. Rapid single-step induction of functional neurons from human pluripotent stem cells. Neuron 78, 785–798 (2013). 10.1016/j.neuron.2013.05.029 23764284 PMC3751803

[78] Malik A. N. Genome-wide identification and characterization of functional neuronal activity-dependent enhancers. Nat Neurosci 17, 1330–1339 (2014). 10.1038/nn.3808 25195102 PMC4297619

[79] Bray N. L., Pimentel H., Melsted P. & Pachter L. Near-optimal probabilistic RNA-seq quantification. Nature Biotechnology 34, 525–527 (2016). 10.1038/nbt.3519 27043002

[80] Love M. I., Huber W. & Anders S. Moderated estimation of fold change and dispersion for RNA-seq data with DESeq2. Genome Biol 15, 550 (2014). 10.1186/s13059-014-0550-8 25516281 PMC4302049

[81] Cahill K. M., Huo Z., Tseng G. C., Logan R. W. & Seney M. L. Improved identification of concordant and discordant gene expression signatures using an updated rank-rank hypergeometric overlap approach. Scientific Reports 8, 9588 (2018). 10.1038/s41598-018-27903-2 29942049 PMC6018631

[82] Kim D., Langmead B. & Salzberg S. L. HISAT: a fast spliced aligner with low memory requirements. Nature Methods 12, 357–360 (2015). 10.1038/nmeth.3317 25751142 PMC4655817

[83] Zhang Y. Model-based Analysis of ChIP-Seq (MACS). Genome Biology 9, R137 (2008). 10.1186/gb-2008-9-9-r137 18798982 PMC2592715

[84] Stark R. & Brown G. DiffBind: differential binding analysis of ChIP-Seq peak data. R package version 100, 2–21 (2011).

[85] Yu G., Wang L.-G. & He Q.-Y. ChIPseeker: an R/Bioconductor package for ChIP peak annotation, comparison and visualization. Bioinformatics 31, 2382–2383 (2015). 10.1093/bioinformatics/btv145 25765347

[86] Ramírez F., Dündar F., Diehl S., Grüning B. A. & Manke T. deepTools: a flexible platform for exploring deep-sequencing data. Nucleic acids research 42, W187–W191 (2014). 24799436 10.1093/nar/gku365 PMC4086134

[87] Skene P. J., Henikoff J. G. & Henikoff S. Targeted in situ genome-wide profiling with high efficiency for low cell numbers. Nat Protoc 13, 1006–1019 (2018). 10.1038/nprot.2018.015 29651053

[88] Meers M. P., Bryson T. D., Henikoff J. G. & Henikoff S. Improved CUT&RUN chromatin profiling tools. Elife 8 (2019). 10.7554/eLife.46314 PMC6598765 31232687

[89] Langmead B. & Salzberg S. L. Fast gapped-read alignment with Bowtie 2. Nat Methods 9, 357–359 (2012). 10.1038/nmeth.1923 22388286 PMC3322381

[90] Li H. The Sequence Alignment/Map format and SAMtools. Bioinformatics 25, 2078–2079 (2009). 10.1093/bioinformatics/btp352 19505943 PMC2723002

[91] Ramírez F., Dündar F., Diehl S., Grüning B. A. & Manke T. deepTools: a flexible platform for exploring deep-sequencing data. Nucleic Acids Res 42, W187–191 (2014). 10.1093/nar/gku365 24799436 PMC4086134

[92] Bentsen M. ATAC-seq footprinting unravels kinetics of transcription factor binding during zygotic genome activation. Nature Communications 11, 4267 (2020). 10.1038/s41467-020-18035-1 PMC7449963 32848148

[93] Fisher R. A. On the Interpretation of χ^2^ from Contingency Tables, and the Calculation of P. Journal of the Royal Statistical Society 85, 87–94 (1922). 10.2307/2340521

