# Supplementary material for: Quinone reductase 2 reads H3 serotonylation to support neuronal maturation
Source: bioRxiv. 2026 Mar 19:2026.03.17.712426. Preprint. [Version 2] doi: 10.64898/2026.03.17.712426 (PMC13015520; doi:10.64898/2026.03.17.712426)
Supplement: 23 [file NIHPP2026.03.17.712426v2-supplement-23.pdf]

bioRxiv preprint doi: https://doi.org/10.64898/2026.03.17.712426; this version posted March 19, 2026. The copyright holder for this preprint (which was not certified by peer review) is the author/funder, who has granted bioRxiv a license to display the preprint in perpetuity. It is made available under aCC-BY 4.0 International license.

SUPPLEMENTARY MATERIALS

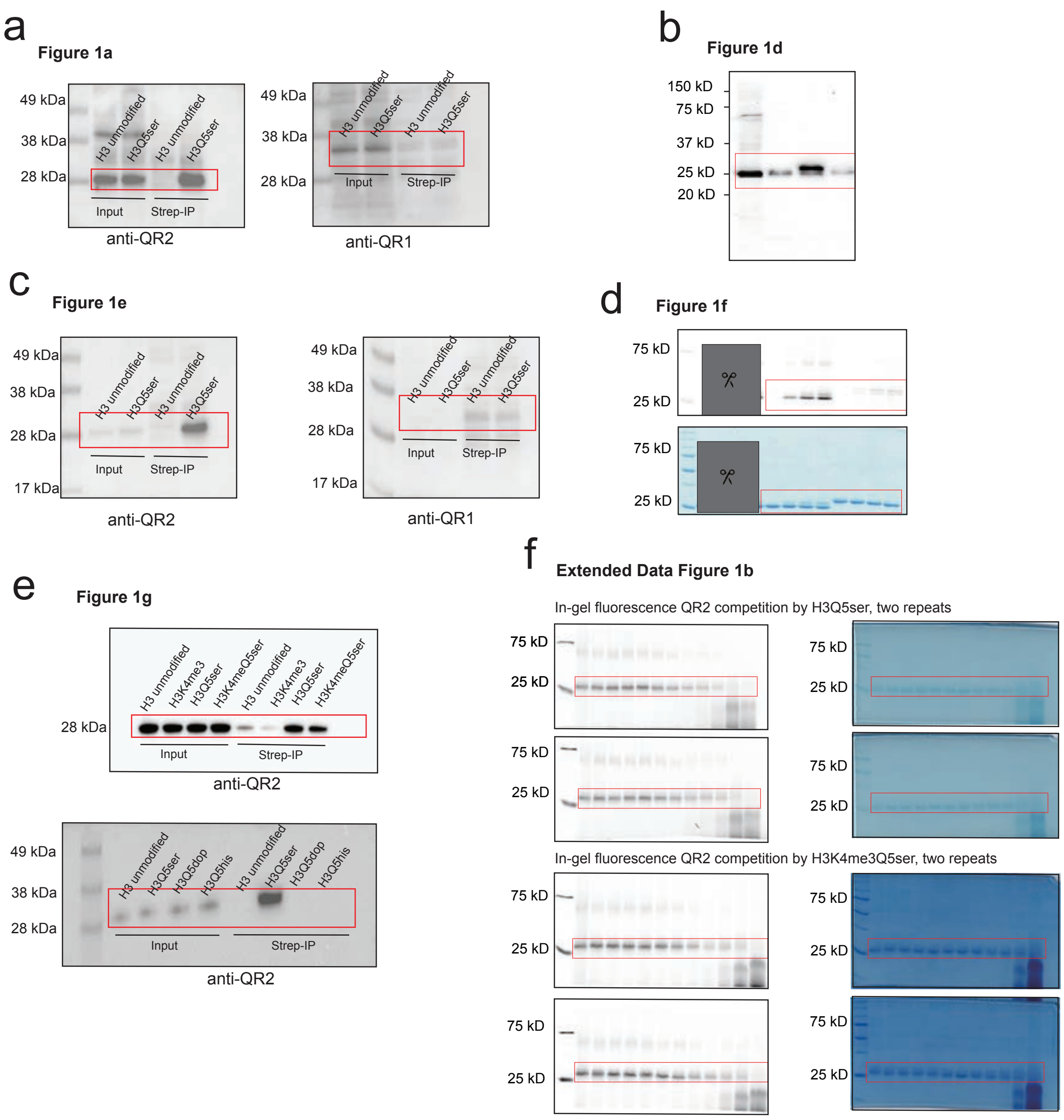

bioRxiv preprint doi: https://doi.org/10.64898/2026.03.17.712426; this version posted March 19, 2026. The copyright holder for this preprint (which was not certified by peer review) is the author/funder, who has granted bioRxiv a license to display the preprint in perpetuity. It is made available under aCC-BY 4.0 International license.

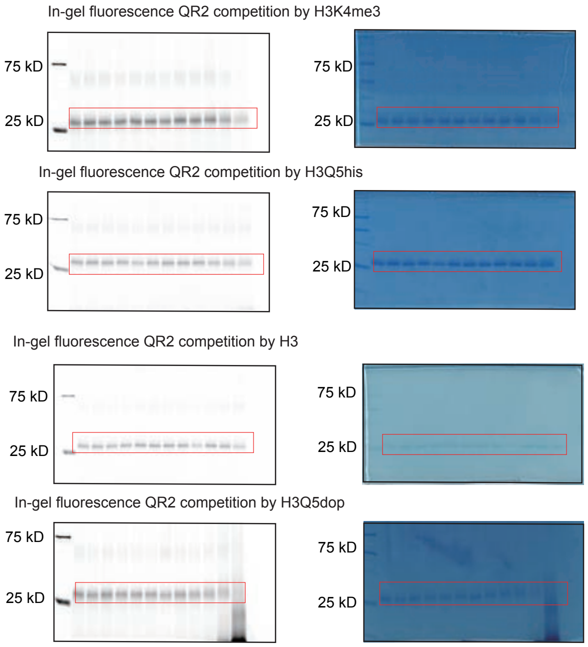

g

Figure 2g

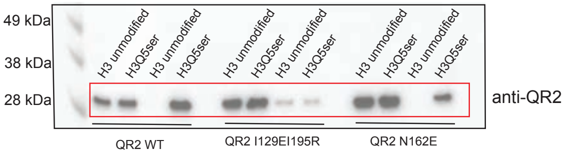

h

Extended Data Figure 5c

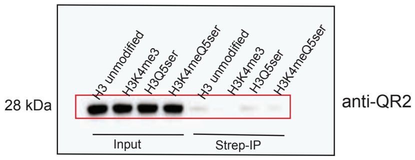

i

Figure 2h

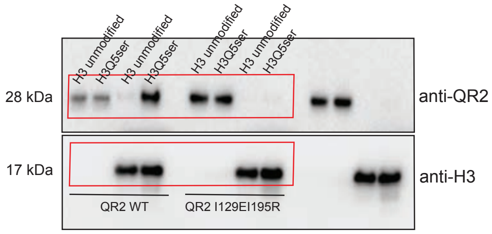

j

Extended Data Figure 5d

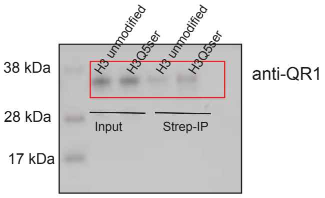

bioRxiv preprint doi: https://doi.org/10.64898/2026.03.17.712426; this version posted March 19, 2026. The copyright holder for this preprint (which was not certified by peer review) is the author/funder, who has granted bioRxiv a license to display the preprint in perpetuity. It is made available under aCC-BY 4.0 International license.

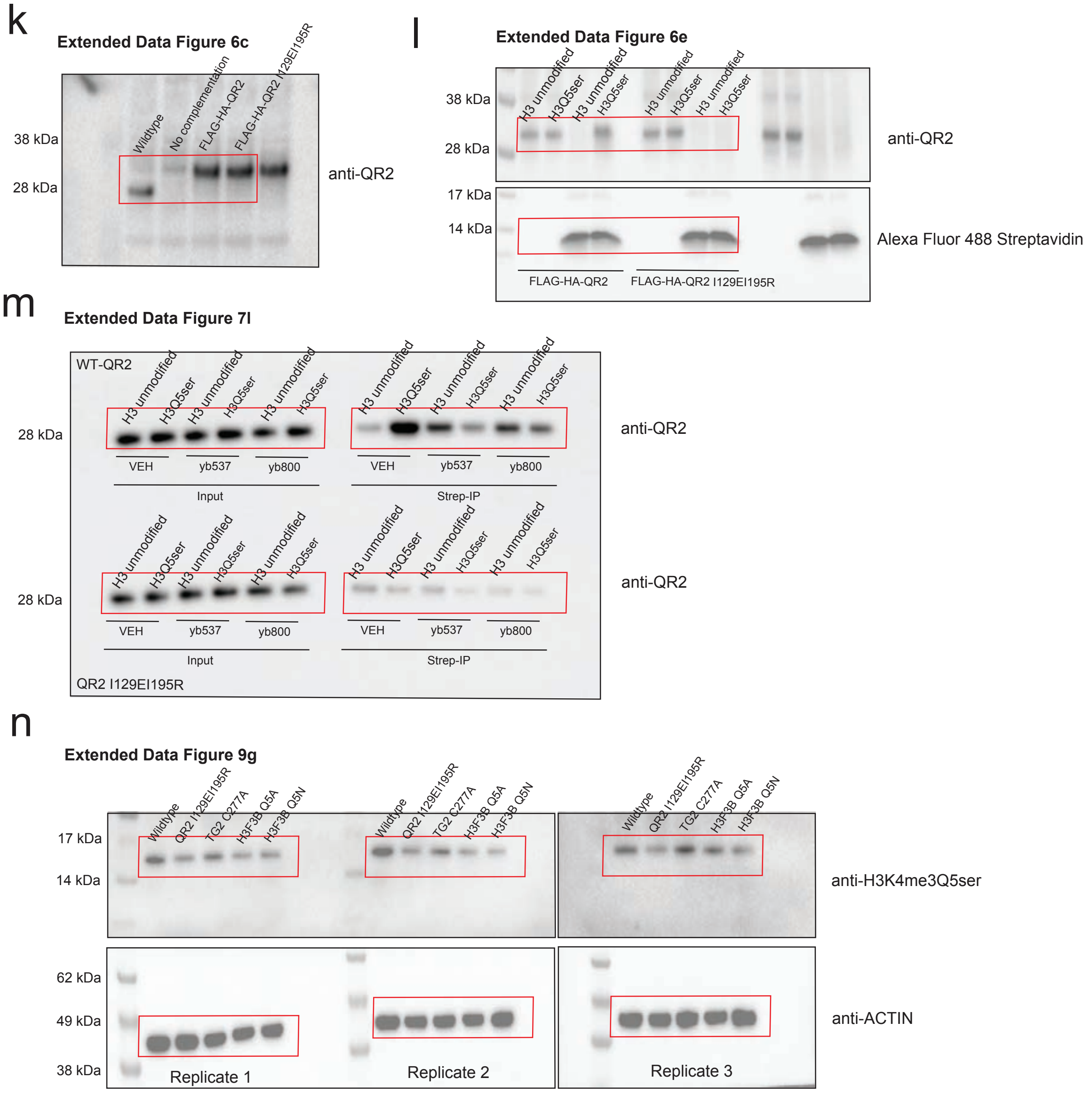

bioRxiv preprint doi: https://doi.org/10.64898/2026.03.17.712426; this version posted March 19, 2026. The copyright holder for this preprint (which was not certified by peer review) is the author/funder, who has granted bioRxiv a license to display the preprint in perpetuity. It is made available under aCC-BY 4.0 International license.

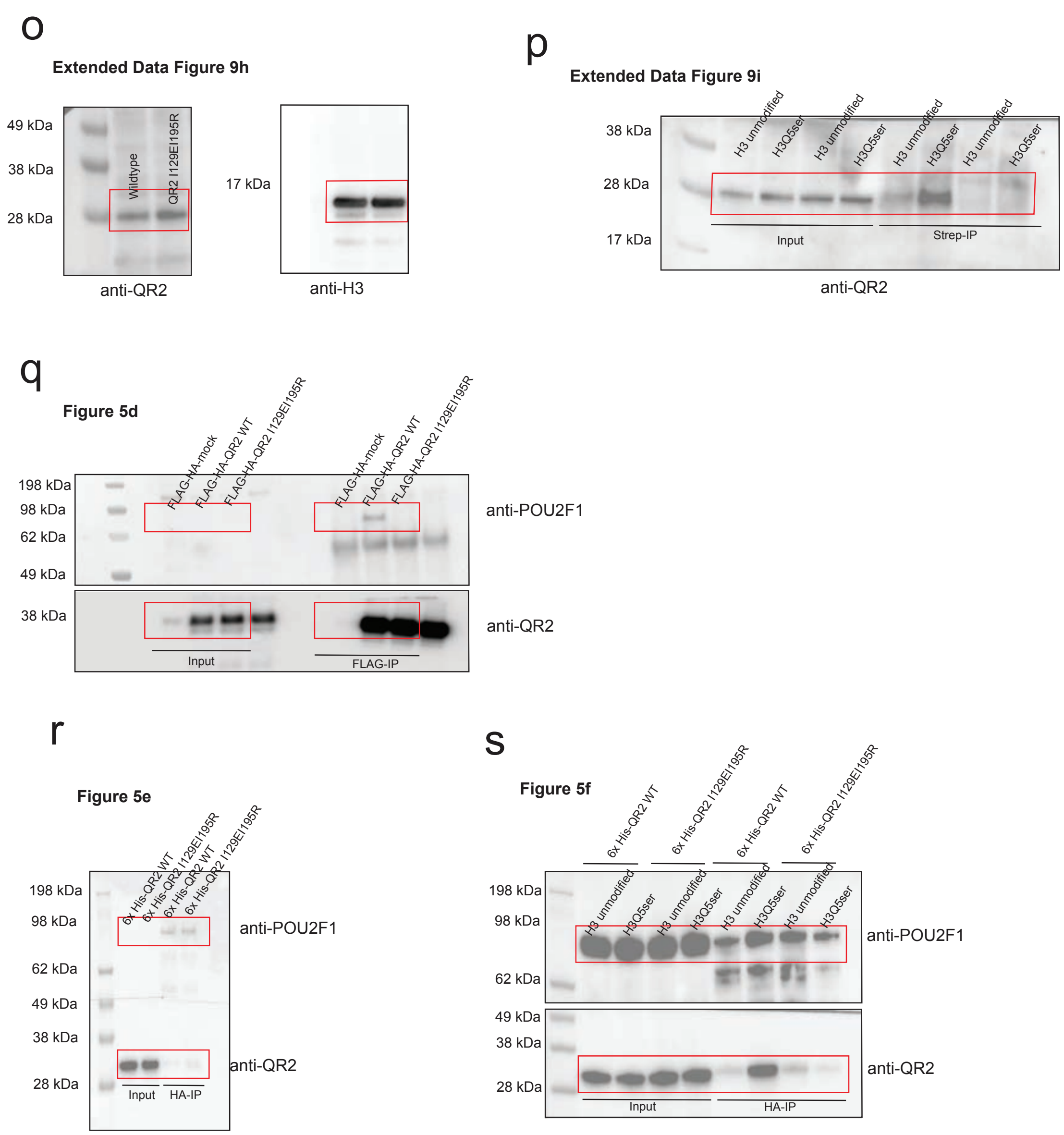

Supplementary Figure 1: Uncropped immunoblots

Uncropped immunoblots related to (**a**) Fig. 1a, (**b**) Fig. 1d, (**c**) Fig. 1e, (**d**) Fig. 1f, (**e**) Fig. 1g, (**f**) Extended Data Fig. 1b, (**g**) Fig. 2g, (**h**) Extended Data Fig. 5c, (**i**) Fig. 2h, (**j**) Extended Data Fig. 5d, (**k**) Extended Data Fig. 6c, (**l**) Extended Data Fig. 6e, (**m**) Extended Data Fig. 7l, (**n**) Extended Data Fig. 9g, (**o**) Extended Data Fig. 9h, (**p**) Extended Data Fig. 9i, (**q**) Fig. 5d, (**r**) Fig. 5e, and (**s**) Fig. 5f.
